# First international external quality assessment scheme of nucleic acid amplification tests for the detection of *Schistosoma* and soil-transmitted helminths, including *Strongyloides*: A pilot study

**DOI:** 10.1371/journal.pntd.0008231

**Published:** 2020-06-16

**Authors:** Piet Cools, Lisette van Lieshout, Rob Koelewijn, David Addiss, Sitara S. R. Ajjampur, Mio Ayana, Richard S. Bradbury, Jason L. Cantera, Daniel Dana, Kerstin Fischer, Rubina Imtiaz, Joyce Kabagenyi, James Lok, James McCarthy, Rojelio Mejia, Zeleke Mekonnen, Sammy M. Njenga, Nurulhasanah Othman, Hongguang Shao, Rebecca Traub, Marjan Van Esbroeck, Jozef Vercruysse, Johnny Vlaminck, Steven A. Williams, Jaco J. Verweij, Jaap J. van Hellemond, Bruno Levecke

**Affiliations:** 1 Laboratory of Parasitology, Ghent University, Merelbeke, Belgium; 2 Leiden University Medical Center, Leiden, The Netherlands; 3 Dutch Foundation for Quality Assessment in Medical Laboratories, Nijmegen, The Netherlands; 4 Erasmus University Medical Center, Rotterdam, The Netherlands; 5 The Task Force for Global Health, Decatur, Georgia, United States of America; 6 Christian Medical College, Vellore, India; 7 Jimma University, Jimma, Ethiopia; 8 Centers for Disease Control and Prevention, Atlanta, Georgia, United States of America; 9 PATH, Seattle, Washington, United States of America; 10 Washington University in St. Louis, St. Louis, Missouri, United States of America; 11 Children Without Worms, Decatur, Georgia, United States of America; 12 Medical Research Council/Uganda Virus Research Institute and London School of Hygiene & Tropical Medicine Uganda Research Unit, Entebbe, Uganda; 13 University of Pennsylvania, Philadelphia, Pennsylvania, United States of America; 14 QIMR Berghofer Medical Research Institute, Herston, QLD, Australia; 15 Baylor College of Medicine, Houston, Texas, United States of America; 16 Kenya Medical Research Institute, Nairobi, Kenya; 17 Universiti Sains Malaysia, Penang, Malaysia; 18 The University of Melbourne, Melbourne, Australia; 19 Institute of Tropical Medicine, Antwerp, Belgium; 20 Smith College, Northampton, Massachusetts, United States of America; 21 University of Massachusetts, Amherst, Massachusetts, United States of America; 22 Elisabeth-TweeSteden Hospital, Tilburg, The Netherlands; Consejo Nacional de Investigaciones Cientificas y Tecnicas, Fundación Mundo Sano, ARGENTINA

## Abstract

**Background:**

Nucleic acid amplification tests (NAATs) are increasingly being used as diagnostic tools for soil-transmitted helminths (STHs; *Ascaris lumbricoides*, *Trichuris trichiura*, *Necator americanus*, *Ancylostoma duodenale* and *A*. *ceylanicum*), *Strongyloides stercoralis* and *Schistosoma* in human stool. Currently, there is a large diversity of NAATs being applied, but an external quality assessment scheme (EQAS) for these diagnostics is lacking. An EQAS involves a blinded process where test results reported by a laboratory are compared to those reported by reference or expert laboratories, allowing for an objective assessment of the diagnostic performance of a laboratory. In the current study, we piloted an international EQAS for these helminths (i) to investigate the feasibility of designing and delivering an EQAS; (ii) to assess the diagnostic performance of laboratories; and (iii) to gain insights into the different NAAT protocols used.

**Methods and principal findings:**

A panel of twelve stool samples and eight DNA samples was validated by six expert laboratories for the presence of six helminths (*Ascaris*, *Trichuris*, *N*. *americanus*, *Ancylostoma*, *Strongyloides* and *Schistosoma*). Subsequently this panel was sent to 15 globally dispersed laboratories. We found a high degree of diversity among the different DNA extraction and NAAT protocols. Although most laboratories performed well, we could clearly identify the laboratories that were poorly performing.

**Conclusions/Significance:**

We showed the technical feasibility of an international EQAS for the NAAT of STHs, *Strongyloides* and *Schistosoma*. In addition, we documented that there are clear benefits for participating laboratories, as they can confirm and/or improve the diagnostic performance of their NAATs. Further research should aim to identify factors that explain poor performance of NAATs.

## Author summary

Tests that detect parasite DNA in human stool are increasingly being used for the diagnosis of infections with intestinal worms, including schistosomiasis. To ensure the quality in diagnostic testing of these parasitic worms, it is important that laboratories evaluate the diagnostic performance of their DNA-based tests. This can best be achieved by participating in an external quality assessment scheme (EQAS). An EQAS involves a blinded process where test results reported by a laboratory are compared to those reported by reference or expert laboratories, allowing for an objective assessment of the diagnostic performance of a laboratory. Currently, such an EQAS for parasitic worms is lacking. We therefore piloted an international EQAS for the diagnosis of parasitic worms involving 15 laboratories in Africa, Asia, Australia, Europe, and North America. Although most laboratories performed well, we could clearly identify those laboratories that may need to improve their test protocol. We found that laboratories were using many different test protocols, and further research should aim to verify whether this has an impact on the performance of the diagnostic outcomes.

## Introduction

Soil-transmitted helminths (STHs, i.e. *Ascaris lumbricoides*, *Trichuris trichiura* and the hookworms *Ancylostoma duodenale*, *Ancylostoma ceylanicum* and *Necator americanus*), *Strongyloides stercoralis* and *Schistosoma* (*S*. *mansoni*, *S*. *haematobium* and *S*. *japonicum*) are parasitic worms that still impose a burden on global health. The STHs affect one-fifth of the world’s population and cause 1.9 million disability adjusted life-years (DALYs) [1]. Schistosomiasis affects 230 million people [2] and has been estimated to cause 1.4 million DALYs [3]. *S*. *stercoralis* has an estimated global prevalence of 370 million [4], but the global burden is still to be determined. Although these infections mainly affect the poorest populations in low- and middle-income countries, hookworms and *S*. *stercoralis* are also found in low-income regions of the United States [5], marginalized communities in Europe [6] and in Indigenous populations in Australia [7,8]. In addition, strongyloidiasis and schistosomiasis are important travel-related diseases, causing substantial morbidity, both in travelers and migrants moving from endemic to non-endemic countries [9,10].

Diagnosis of these parasites is still largely based on the detection of worm eggs or larvae in stool or, in the case of *S*. *haematobium*, eggs in urine samples. However, nucleic acid amplification tests (NAATs) are increasingly being used as diagnostic tools for soil-transmitted helminthiasis, strongyloidiasis and schistosomiasis [11]. For example, NAATs are already considered the standard diagnostics in some clinical laboratories in high-income countries [12,13]. In the public health field, NAATs are being explored as diagnostic tools to assess the distribution of prevalence and infection intensity at the population level in endemic areas [14]. These parasitological indicators are used to guide both the implementation and evaluation of large-scale deworming programs that aim to control the morbidity attributable to soil-transmitted helminthiasis and schistosomiasis [15].

Currently, a large diversity of available NAATs are being applied, but there is no external quality assessment scheme (EQAS) to support the use of these diagnostics [16,17]. However, such EQAS are essential to attain the highest achievable diagnostic quality and to improve health outcomes in terms of care and treatment at the individual level, as well as monitoring of disease burden and response to control at the population level. An EQAS involves a blinded process in which test results reported by a laboratory are compared to those reported by reference or expert laboratories testing the same panel of samples, allowing for an objective assessment of the diagnostic performance of a laboratory [18]. EQAS have already proven to be essential to highlight weaknesses in NAATs used for the diagnosis of other parasitic and tropical diseases such as diarrhea-causing protozoa [19], malaria [20], yellow fever [21] and Zika [22].

In the current study, we piloted an EQAS for the DNA-based detection of *Schistosoma* and STHs, including *Strongyloides*, the so-called Helminth External Molecular Quality Assessment Scheme (HEMQAS). The objectives of this HEMQAS pilot were (i) to investigate the feasibility of designing and delivering a technically and logistically challenging EQAS for the detection of *Schistosoma* and STHs, including *Strongyloides* in both preserved stool and DNA samples, (ii) to evaluate and compare the diagnostic performance of laboratories, and (iii) to gain insights in the diversity of the different NAAT protocols used.

## Methodology

### Ethics

This study was part of work package 1 of the Starworms study (www.starworms.org). The Starworms study was registered (Belgian registration number B670201627755) and approved by the Ethics Committee of the Faculty of Medicine and Health Sciences of Ghent University and the Ghent University Hospital, Belgium (reference number 2016/0266). Local approval was obtained at the Institutional Review Board of Jimma University, Ethiopia (reference number RPGC/547/2016). The school director, teachers, parents or legal guardians, and children were informed about the objectives and procedures of the study. Written informed consent was obtained from the parents or legal guardians of all participating children. Participation was voluntary. In addition to samples obtained from these children, two stool samples were collected from a person living in a non-endemic region, after obtaining a written consent. *S*. *stercoralis* infective third-stage larvae used in this study were from a canine strain maintained in purpose-bred laboratory dogs housed a vivarium at the University of Pennsylvania (Philadelphia, USA). This vivarium is accredited by the International Association for Assessment and Accreditation of Laboratory Animal Care. Dogs were infected with *S*. *stercoralis* and maintained thereafter in accordance with protocols 804798 and 804893 approved by the University of Pennsylvania Institutional Animal Care and Use Committee. All protocols, as well as routine husbandry care of the animals, were carried out in strict accordance with the Guide for the Care and Use of Laboratory Animals of the US National Institutes of Health.

### Nomenclature

In the context of collecting samples for the HEMQAS study, we use the species names of the helminths where appropriate. In the context of the HEMQAS panel, given that not all experts laboratories used species specific assays (see further), we refer to the respective HEMQAS samples as being validated for *Ascaris*, *Ancylostoma*, *Schistosoma*, *Strongyloides* and *Trichuris* (even though microscopic screening and/or epidemiology allowed speciation). We used the term *N*. *americanus* throughout the manuscript, given that all expert laboratories used species specific assays for *N*. *americanus*.

### Designing and delivering HEMQAS

**Fig 1:**
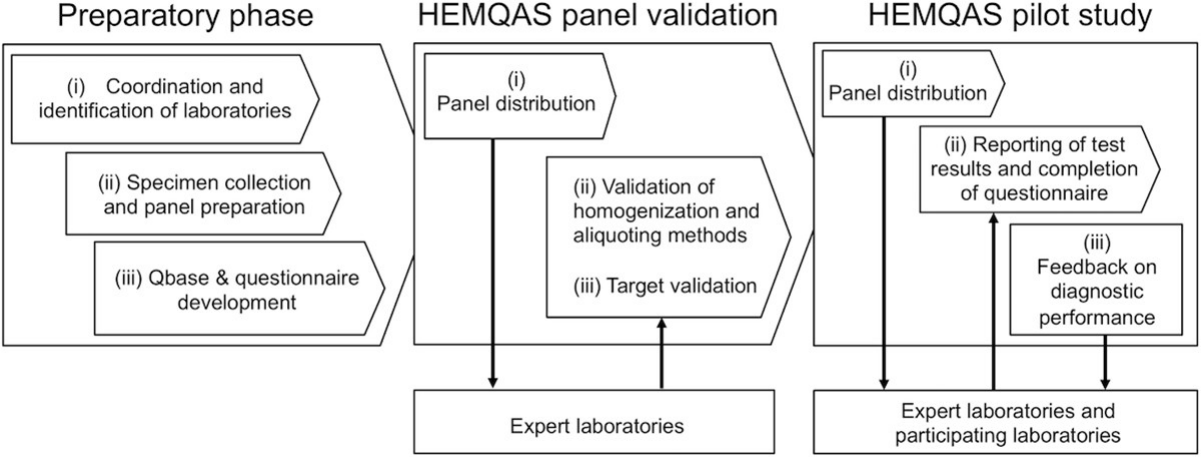
Overview of the different steps, the expert and participating laboratories of the HEMQAS pilot study.

We designed and delivered HEMQAS through three consecutive steps (**Fig 1**). In the preparatory phase, we identified the expert and participating laboratories, collected the samples and prepared the panel. Expert laboratories were identified and defined based on their scientific track record on molecular diagnostics for STHs and *Schistosoma* and authority in this field. In addition, we developed a questionnaire to gain insights into the diversity in NAAT protocols. Next, the HEMQAS panel was distributed to the expert laboratories, which validated the procedures of homogenizing and aliquoting samples, and the targets in the panel. In a final step, the HEMQAS panel was distributed to the participating laboratories, which reported their findings and received feedback on their diagnostic performance. In the following paragraphs we will discuss each of the three steps in more detail.

### Preparatory phase

#### (i) Coordination and identification of laboratories

The study was coordinated by the Laboratory of Parasitology of Ghent University and the Department of Medical Microbiology and Infectious diseases (Erasmus University Medical Center), which is the coordinating center for parasitological EQAS of the Dutch Foundation for Quality Assessment in Medical Laboratories (SKML; www.skml.nl). The SKML has long-term experience in setting up EQAS and has already established an international EQAS for the DNA-based diagnosis of intestinal protozoa [19].

We aimed to include expert laboratories for the validation of the HEMQAS panel in such way that each target (*Ascaris*, *Trichuris*, *Ancylostoma*, *N*. *americanus*, *Strongyloides* and *Schistosoma*) was validated by a minimum of five expert laboratories geographically dispersed around the world. Expert laboratories were considered if their NAAT protocols had been published or when their expertise on NAATs was internationally acknowledged. Based on these criteria, two clinical and four research laboratories across Australia (n = 1), Europe (n = 3) and the USA (n = 2) were identified as expert laboratories (**Table 1**). Each of these laboratories committed to examine the HEMQAS panel in a blinded way and to discuss the overall obtained results applying a priori defined criteria explained below.

**Table 1:** The expert and participating laboratories of the HEMQAS pilot study. The order of the list does not correspond with the order of the laboratories in **Table 5**.

Country	Institution/Organization	Laboratory/Department	Laboratory type
**Expert laboratories**			
Australia	QIMR Berghofer Medical Research Institute	The Clinical Tropical Medicine Laboratory	Research laboratory
The Netherlands	Erasmus Medical Center	Department of Medical Microbiology and Infectious Diseases	Clinical laboratory
The Netherlands	Elisabeth TweeSteden Hospital	Laboratory of Medical Microbiology and Immunology	Clinical laboratory
The Netherlands	Leiden University Medical Center	Department of Parasitology	Research laboratory
USA	Baylor College of Medicine	Laboratory of Clinical Parasitology and Diagnostics	Research laboratory
USA	Smith College and the University of Massachusetts	Department of Biological Sciences and Biochemistry; Program in Molecular and Cellular Biology	Research laboratory
**Participating laboratories**			
Australia	University Melbourne	Melbourne Veterinary School	Research laboratory
Belgium	Institute of Tropical Medicine	Central Laboratory for Clinical Biology	Clinical laboratory
India	Christian Medical College, Vellore	The Wellcome Trust Research Laboratory	Research laboratory
Kenya	Kenya Medical Research Institute	Eastern and Southern Africa Centre of International Parasite Control	Research laboratory
Malaysia	Universiti Sains Malaysia	Institute for Research in Molecular Medicine	Research laboratory
Uganda	Medical Research Council/Uganda Virus Research Unit & London School of Hygiene and Tropical Medicine Uganda Research Unit	Immunity and Vaccines Programme	Research laboratory
USA	Centers for Disease Control and Prevention	Parasitic Diseases Branch	Research laboratory
USA	PATH	Diagnostics Program	Research laboratory
USA	Washington University in St. Louis	Infectious Diseases Division	Research laboratory

For the actual HEMQAS pilot study, nine laboratories were identified within the network of the HEMQAS coordinators (**Table 1**). Here, we only considered laboratories for participation if they had an operational NAAT for at least two of the six targets. We also aimed to include participants from both endemic and non-endemic countries. Furthermore, all six expert laboratories that were involved in the validation of the HEMQAS samples were also included as participating laboratories, resulting in a total of 15 laboratories. Eleven laboratories were research laboratories and four of the 15 laboratories were clinical laboratories. Two laboratories were from Africa, two from Asia, two laboratories from Australia, four from Europe, and five from the USA. Participating in the study was free of charge.

#### (ii) Collection of specimens and preparation of the panel

The HEMQAS panel included both a stool panel and a DNA panel. For the stool panel, a total of 12 stool samples were collected. Ten of these clinical stool samples were collected in Jimma (Ethiopia) as part of the Starworms study [23]. Stool samples from school-aged children in which one of the STH species or *S*. *mansoni* had been detected using the Kato-Katz technique [23] were preserved in ethanol by filling half of a 50 ml Falcon tube with stool and suspending this in 25 ml of 96% ethanol. These ten stool samples were selected, as much as possible, to include mixed infections in order to maximize the total number of targets while minimizing the number of samples. The samples were kept refrigerated until shipment at ambient temperature to the Laboratory of Parasitology (Ghent University, Belgium). Two stool samples were collected from one person in Belgium. One of these stool samples was spiked with *S*. *stercoralis* third-stage larvae at a concentration of 200 larvae per gram (**Table 2**) before being further diluted circa 40 times, the other stool sample was used as a negative control. All stool samples were homogenized, further diluted as needed, and aliquoted according to the in-house method of SKML applied for its protozoa EQAS panel [19].

**Table 2:** Origin and type of worm material used for the HEMQAS panel.

Species	Origin	Host	Type of material
*Ascaris lumbricoides*	Jimma, Ethiopia	Human	Adult worm
*Necator americanus*	Leiden, The Netherlands	Human	Third-stage larvae
*Schistosoma mansoni*	Rotterdam, The Netherlands	Hamster	Adult worms
*Strongyloides stercoralis*	Philadelphia, United States	Dog	Third-stage larvae

For the HEMQAS DNA panel, worm materials were obtained from different partners (**Table 2**). DNA from adult *A*. *lumbricoides* worms (only the heads were used), *N*. *americanus* third-stage larvae, *S*. *stercoralis* infective third-stage larvae and *S*. *mansoni* adult worms was extracted by one expert laboratory (Elisabeth-TweeSteden Hospital, Tilburg, The Netherlands). For this, the QIAamp DNA Mini Kit (Qiagen) was used according to the manufacturer’s guidelines. The quantity of the DNA was measured using the Qubit fluorometer. From each DNA extract, a tenfold dilution was made and included in the DNA panel, resulting in a total of eight DNA extracts.

#### (iii) Development of QBase and questionnaire

In order to gain insights into the different NAAT protocols used by the participants, we developed a questionnaire that was incorporated into QBase. QBase is an online results submission tool developed and used by SKML for other EQAS. It was adapted to fit the specific needs of this pilot study. A summary manual and an extended manual were developed to introduce all participants to this submission tool (**S1 File**).

### Validation of the HEMQAS panel

Following the distribution of the panel to the expert laboratories, the panel was validated. The validation of the panel was two-fold and was based on the procedures previously described by Schuurs and coworkers [19]. First, we validated the procedures for homogenizing and aliquoting stool samples. Second, we validated the targets in the panel. In the following paragraphs we will discuss each of the steps in more detail.

#### (i) Distribution of the panel

The HEMQAS panel for validation consisted of twenty samples (twelve ethanol-preserved stool samples and eight DNA samples) and was sent out to the six expert laboratories who received the samples blinded. For validation, the panels were organized in such a way that each expert laboratory received three or four samples as quintuplicates (i.e., five aliquots) and the remaining 16 to 17 samples as single aliquots, and that the different quintuplicates were equally dispersed over the expert laboratories. Given that there were six laboratories and a panel of twenty samples, some expert laboratories received three samples as quintuplicates and 17 samples as single aliquots, and the other expert laboratories received four samples as quintuplicates and 16 samples as single aliquots. Expert laboratories were asked to remove the ethanol from the ethanol-preserved stool samples according to a standard SKML protocol that was sent along with the samples (see **S2 File**). Further, expert laboratories were asked to process and analyze all samples according to their in-house DNA extraction and NAAT protocol.

#### (ii) Validation of stool homogenization and aliquoting methods

Eggs of STHs and *Schistosoma* and larvae of *S*. *stercoralis* can be unequally dispersed in a stool sample. In order to validate the homogenization and aliquoting procedures, quintuplicates of each sample were analyzed by a single laboratory. We concluded that homogenization and aliquoting procedures were appropriate when the standard deviation of the Cq-values of the targets with the lowest Cq-values (i.e., the highest DNA concentration) across the quintuplicates did not exceed two. We did not consider this criterion for targets for which the Cq-values were high (i.e., the lowest DNA concentration), as a large variation in Cq-values is already expected (the variation in Cq-values between the aliquots of the same samples increases as the DNA concentration drops [24]).

#### (iii) Validation of the targets

At least five expert laboratories assessed all samples for all targets, except for *Schistosoma*, which was assessed by four expert laboratories. All laboratories were blinded to the microscopy data and the outcome of the other laboratories. Expert laboratories were asked to report their results to SKML within one month using a standard Excel file. Reports were compiled by SKML and predefined criteria were applied to validate samples as positive, negative or educational for a specific target (i.e., *Ascaris*, *Trichuris*, *N*. *americanus*, *Ancylostoma*, *Strongyloides* and *Schistosoma*). A sample was validated as positive for a particular target when the following three criteria were fulfilled: (i) all quintuplicates analyzed by one of the expert laboratories was positive for that target, (ii) the standard deviation of the Cq-values of the quintuplicates was less than two cycles, and (iii) all other expert laboratories confirmed the presence of that particular target. These criteria guarantee that DNA of the target can be detected by state-of-the-art NAAT variants in all aliquots of the sample, and therefore, a positive result by the participating laboratories can be demanded. A sample was classified as negative for particular target when the two following criteria were fulfilled: (i) all quintuplicates analyzed by the expert laboratory were negative for that particular target and (ii) all other expert laboratories confirmed the absence of that particular target. Samples that were not validated as positive or negative for a specific target were considered as educational for this particular target, meaning that the samples contained such a low parasite load that it was not consistently detected by all the expert laboratories. Therefore, a positive result cannot be demanded from each participating laboratory. These criteria are commonly applied by variety of EQAS organizations, including but not limited to SKML. The results of this validation were compiled, shared between expert laboratories (see **S3 File**) and discussed during a teleconference call. Based on the results, it was decided to keep all 12 stool and 8 DNA samples in the final HEMQAS panel. The composition of the HEMQAS stool and DNA panel is summarized in **Table 3**. The 12 stool and 8 DNA samples included targets for five helminth targets (*Ascaris*, *Trichuris*, *N*. *americanus*, *Strongyloides* and *Schistosoma*). Six of the 20 samples were validated as positive for *Ascaris*, two as positive for *Trichuris*, seven as positive for *N*. *americanus*, five as positive for *Schistosoma* and one as positive for *Strongyloides*. Ten of the 20 samples were validated negative for *Ascaris*, twelve for *Trichuris*, sixteen for *Strongyloides* and fifteen for *Schistosoma*. The panel did not include *Ancylostoma*, and all samples were validated as negative for this genus. The educational samples included four for *Ascaris*, six for *Trichiura*, two for *N*. *americanus* and three for *Strongyloides*. For *Schistosoma*, there were no educational samples.

**Table 3:** Composition of the HEMQAS stool (ST) and the DNA panel. Red cells represent samples that are classified as negative for that particular target; green cells represent samples that are classified as positive for the target; orange cells represent samples that are classified as educational for the target.

Sample ID	*Ascaris*	*Trichuris*	*Necator* *americanus*	*Ancylostoma*	*Strongyloides*	*Schistosoma*
ST1						
ST2						
ST3						
ST4						
ST5						
ST6						
ST7						
ST8						
ST9						
ST10						
ST11						
ST12						
DNA1						
DNA2						
DNA3						
DNA4						
DNA5						
DNA6						
DNA7						
DNA8						

### HEMQAS pilot study

#### (i) Distribution of the panel

The HEMQAS panels were sent to all participating laboratories at ambient temperature using an express courier. For each laboratory, the stool and DNA panel samples were placed in protective sealed envelopes. Per stool and DNA sample, we only sent one aliquot of 250 μl and 50 μl, respectively. No quintuplicates were sent in the pilot study. Each package also contained instructions on how to remove the ethanol from stool, prior to commencing DNA extraction procedures (see **S2 File**). No information on the outcome of the validation was shared to the participating laboratories.

#### (ii) Reporting of the test results and completion of the questionnaire

All participating laboratories were asked to process the panel according to their in-house DNA extraction and NAAT protocols, and to report the results using the QBase submission tool. They were also questioned about the main characteristics of both their DNA extraction procedures and NAAT protocols by means of a questionnaire. Participants were allowed two months to complete the analysis, submit their results and complete the questionnaire. This is longer than what is normally giving during an EQAS by SKML, but the extra time was given to anticipate logistical issues such as packages delayed at customs, and the need of laboratories to become acquainted with the online QBase submission software.

#### (iii) Feedback on the diagnostic performance

After receiving the results from all participants, results were compiled and reported back to all participants by SKML. In these reports, all participants received a complete anonymized list of all submitted results with only their own laboratory recognizable. An example of such a report is provided in **S4 File**. For the current study, the raw data were analyzed as described below.

### Statistical analysis

We analyzed the diagnostic performance of the different NAATs across laboratories and targets by assessing the number of false positive (FP) and false negative (FN) test results. Educational samples were not considered in these analyses because interpretation of these results requires more observations. We assessed the variation in DNA extraction and NAAT protocols across the different participants. We did not explore associations between DNA extraction or NAAT protocols and diagnostic performance. This is because the pilot study was not designed nor powered to identify possible factors that may explain the variation in diagnostic performance across laboratories. To gain insights into the variation in quantitative test results, we assessed the variation in the reported Cq-values across the different targets and individual samples. The stability of the DNA and stool panel was analyzed by comparing the Cq-values obtained by the expert laboratories during the panel validation with those reported approximately six months later in the HEMQAS pilot study.

## Results

### Description of the DNA extraction and NAAT protocols

All participating laboratories completed the online questionnaire. A summary of the different DNA extraction and NAAT protocols used by the 15 laboratories is provided in **Table 4**. All laboratories used a commercial DNA extraction kit. Twelve laboratories applied a procedure in which the helminth eggs were mechanically broken by bead beating, five included a freeze-thaw cycle in the DNA extraction protocol, and two included a protease treatment, all in order to increase DNA yield from the helminth eggs. There was a great deal of variation in the type of beads (n = 11), bead beaters (n = 10) and DNA extraction kits (n = 10) used. Both the volume of stool suspensions used for DNA extraction and the elution volume differed substantially between laboratories, ranging from 50 to 500 μl and from 50 to 200 μl, respectively. All laboratories used quantitative PCR (qPCR) as NAAT, but in different formats. Six laboratories used a multiplex qPCR and nine laboratories used different singleplex qPCRs. All qPCR assays used hydrolysis probes to detect amplicons. There was a large diversity in master mixes used, with almost every laboratory using a different master mix. Five of the fifteen laboratories analyzed the panels for the presence of all targets. Five laboratories had NAATs for all targets except *Schistosoma*, two laboratories had all targets except *Schistosoma* and *Strongyloides*, two laboratories only had NAAT available for *Strongyloides* and *Schistosoma*, and one laboratory had qPCR protocols for *Schistosoma*, *Strongyloides* and *N*. *americanus*. The DNA template volume used in the NAATs ranged from 1 μl to 10 μl and the corresponding stool suspension used per NAAT reaction ranged from 1.5 μl to 36.4 μl.

**Table 4:** Overview of the different DNA extraction and NAAT protocols used in the HEMQAS pilot study. The number between brackets corresponds to the number of laboratories. ITS-1, intergenic transcribed spacer-1; ITS-2, intergenic transcribed spacer-2; 18S rRNA, 18S ribosomal ribonucleic acid; 28S rRNA, 28S ribosomal ribonucleic acid.

DNA extraction
**Bead beating**: Yes (12), No (3)
**Type of beads**: MagNA Lyser Green Beads (Roche) (1); Garnet beads 0.7 mm (SanBio) (1); Zirconia/silica beads 0.5 mm (Daintree Scientific) (1); Silica beads (1); Included in FastDNA SPIN kit for Soil (MP Biomedicals) (4); Mixture of ceramic and silica particles (1); Included in ISOLATE II Faecal DNA extraction kit (Bioline) (1); Zirconium beads 1.5 mm (Benchmark Scientific) (1); Included in Precellys Lysing Kit (Bertin technologies) (1)
**Bead beater**: TissueLyser LT (Qiagen) (1); FastPrep (MP Biomedicals) (6); Mini-Beadbeater (Biospec Products) (1); Beadbug (Benchmark Scientific) (1); Precellys 24 Tissue Homogenizer (Bertin technologies) (1); Vortex (2)
**Proteinase K pretreatment**: Yes (4), No (11)
**Freeze-thaw cycle**: Yes (5), No (10)
**DNA extraction kit**: MagnaPure (Roche) (1); Isolate II Faecal DNA Kit (Bioline) (1); Maxwell (Promega) (1); Maxwell RSC PureFood GMO and Authentication kit (Promega) (1); FastDNA SPIN Kit for Soil (MP-Biomedicals) (5); QIAamp DNA Mini Kit (Qiagen) (1); DNeasy Blood and Tissue Kit (Qiagen) (2); QIAamp Stool Kit (Qiagen) (1); DNeasy PowerSoil Kit (formerly Powersoil kit sold by MO BIO) (3); QIA Symphony (Qiagen) (1)
**NAAT**
**NAAT technology**: qPCR (15)
**qPCR format**: Singleplex (9), Multiplex (6)
**Detection**: Hydrolysis probes (15)
**Master mix**: Qiagen (1); Quantitect (Qiagen) (2); HotStarTaq MasterMix (Qiagen) (2); Applied Biosystems (4); VWR (1); Roche (1); Promega (1); Platinum qPCR Supermix-UDG with ROX (Thermofisher) (1); TaqPath ProAmp Master (Thermofisher) (1); Technologies Brilliant II QPCR Master Mix (Agilent) (1)
**Stool volume used for DNA extraction (mean, range):** 223 μl, 50–500 μl
**Elution volume (mean, range):** 109 μl, 50–200 μl
**DNA used for NAAT (mean, range):** 3.2 μl, 1–10 μl
**Stool volume used per NAAT reaction (mean, range):** 8.6 μl, 1.5–36.4 μl
***Ascaris* primer and probe target:** ITS-1 (10), non-coding repetitive DNA sequence (2)
***Trichuris* primer and probe target:** 18S rRNA (4), 28S rRNA (1), ITS-1 (1), non-coding repetitive DNA sequence (6)
***Necator* primer and probe target:** ITS-1 (1), ITS-2 (6), non-coding repetitive sequence (5)
***Schistosoma* primer and probe target:** 28S rRNA (1), cytochrome c oxidase (1), non-coding repetitive DNA sequence (1), ITS-2 (4)
***Strongyloides* primer and probe target:** 18S rRNA (8), non-coding repetitive sequence (5)

### Stability of the targets over time

The mean difference of the Cq-values (± standard deviation) for all stool samples reported as positive by the expert laboratories during the validation of the panel and those reported approximately six months later during the actual HEMQAS pilot study by the same laboratories was 1.8 ± 0.4 for *Ascaris*, -1.6 ± 1.7 for *N*. *americanus*, 0.8 ± 0.9 for *Schistosoma* and 0.4 ± 0.6 for *Trichuris*. However, one stool sample that was validated as positive for *Trichuris* by the five expert laboratories was found negative by three of these laboratories during the pilot study. For the DNA samples, the mean difference (± standard deviation) was 0.6 ± 0.1 for *Ascaris*, 0.5 ± 0.0 for *N*. *americanus*, 0.1 ± 0.1 for *Schistosoma* and 0.0 ± 0.6 for *Strongyloides*. Given the minor variation over time of all targets, except for one *Trichuris-*positive stool sample, we concluded that the targets in both the stool and DNA panel were stable over time.

### Diagnostic performance

The diagnostic performance assessed by the number of false positives (FPs) and false negatives (FNs) across targets and/or laboratories is shown in **Table 5**. Of the total of 975 negative targets that were analyzed by the different laboratories, thirteen were FP test results. All of the FP test results were reported by only two of the laboratories (laboratory B and N). Eight of these were FP *Strongyloides* results reported by laboratory B and three were FP *Schistosoma* results reported by laboratory N. Laboratory N also reported a FP *N*. *americanus* result. Across the 240 positive targets, there were a total of 30 FN test results. Of these, 23 FN test results were reported by three laboratories, including laboratory M (n = 16), laboratory F (n = 4) and laboratory H (n = 3). The other seven FNs were reported by seven different laboratories. Three laboratories reported no FP and no FN test results. The number of educational samples found positive for each target and laboratory are shown in **Table 5**.

**Table 5:** The performance of 15 laboratories for the detection of six helminths in stool and DNA. The numbers in the second row represent the total number of true negatives (TN), true positives (TP) and educational samples (Ed) per target. The laboratories are shown in the left column. The body of the table shows the number of false-positive (FP), false-negative (FN) and educational (Ed) test results per laboratory and per target. Blue cells indicate FP results, red cells indicate FN results, green cells indicate the absence of FP and FN results, black cells indicate that a NAAT has not been performed by a laboratory. The numbers in the blue, red and white cells indicate the number of FP, FN or Ed results, respectively. The ‘All targets’ column, represents per laboratory across all targets the ratio of the totals of FP, FN and educational positives (EP) over the total number of TN, TP and Ed.

	*Ascaris*			*Trichuris*			*N*. *americanus*			*Ancylostoma*			*Strongyloides*			*Schistosoma*			All targets		
	TN	TP	Ed	TN	TP	Ed	TN	TP	Ed	TN	TP	Ed	TN	TP	Ed	TN	TP	Ed			
	10	6	4	12	2	6	11	7	2	20	0	0	16	1	3	15	5	0			
Lab	FP	FN	EP	FP	FN	EP	FP	FN	EP	FP	FN	EP	FP	FN	EP	FP	FN	EP	**FP/TN**	**FN/TP**	**EP/Ed**
A			3			6			1						3		1		**0/84**	**1/21**	**13/15**
B													8		3				**8/31**	**0/6**	**3/3**
C															2				**0/31**	**0/6**	**2/3**
D			3			6			0						2				**0/84**	**0/21**	**11/15**
E			3		1	0			1						2				**0/69**	**1/16**	**6/15**
F			2		1	3		3	0						2				**0/84**	**4/21**	**7/15**
G			3		1	0			0						2				**0/84**	**1/21**	**5/15**
H		2	2		1	0			1						2				**0/69**	**3/16**	**5/15**
I			3			0			1	1									**1/53**	**0/15**	**4/12**
J			3		1	0			1						2				**0/69**	**1/16**	**6/15**
K			2		1	4			0										**0/53**	**1/15**	**6/12**
L			2		1	0			0						2				**0/69**	**1/16**	**4/15**
M		6	0		2	0		7	0					1	0				**0/69**	**16/16**	**0/15**
N							1	1	0						2	3			**4/42**	**1/13**	**2/5**
O			2			0			0						1				**0/84**	**0/21**	**3/15**
**Total**	**0**	**8**	**28**	**0**	**9**	**19**	**1**	**11**	**5**	**1**	**0**		**8**	**1**	**25**	**3**	**1**		**13/975**	**30/240**	**77/185**

### Quantification

**Fig 2:**
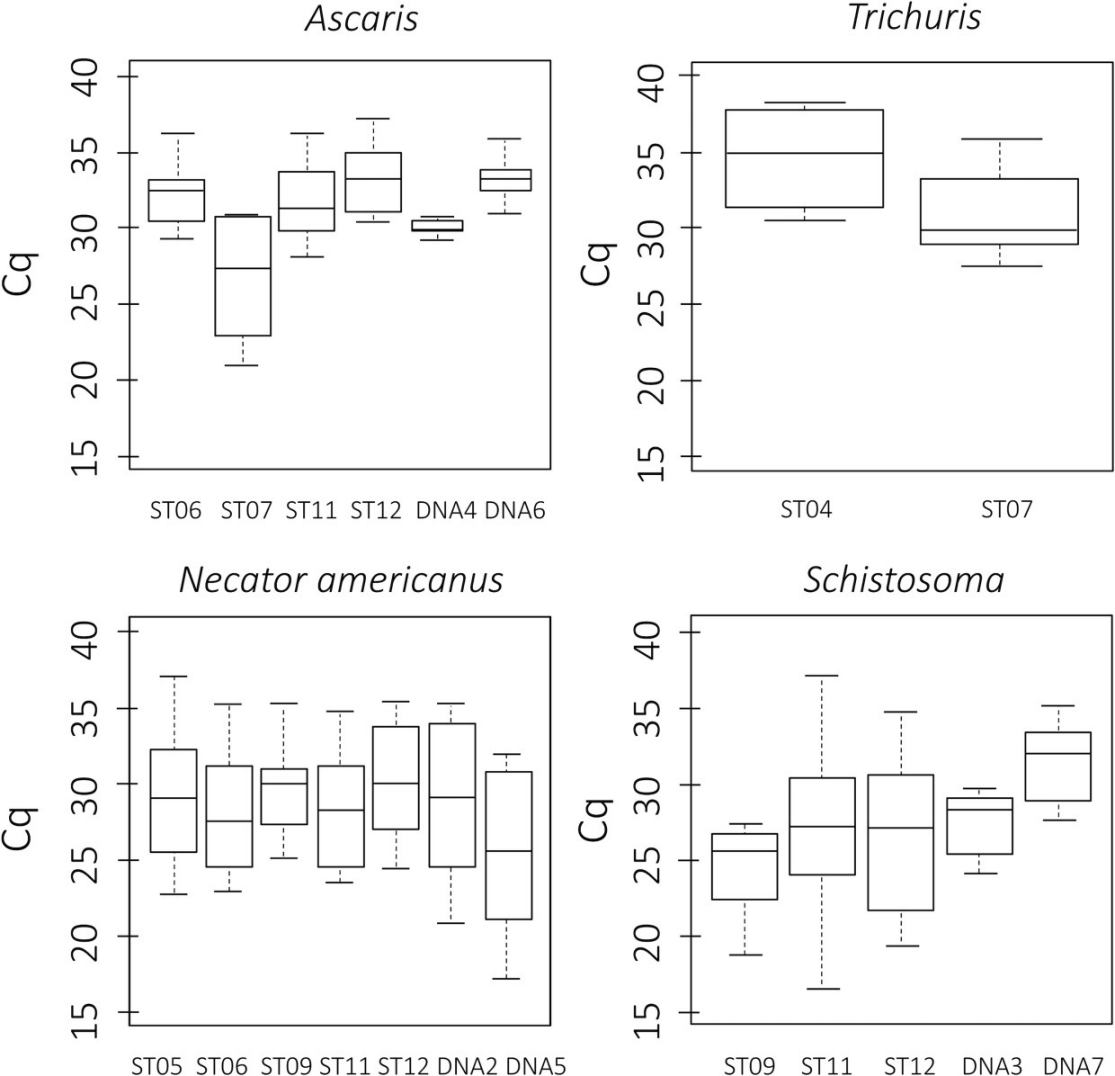
The variation in the reported Cq-values across the different targets and samples. The Cq-values reported by the different participants for the different targets is shown on the Y-axis. The boxes represent the interquartile range; the lines in the boxes represent the median values; the upper and lower whiskers represent the 95^th^ and the 5^th^ percentile, respectively; and the dots represent outliers. The X-axis represents the different samples of the HEMQAS panel (see **Table 3**).

**Fig 3:**
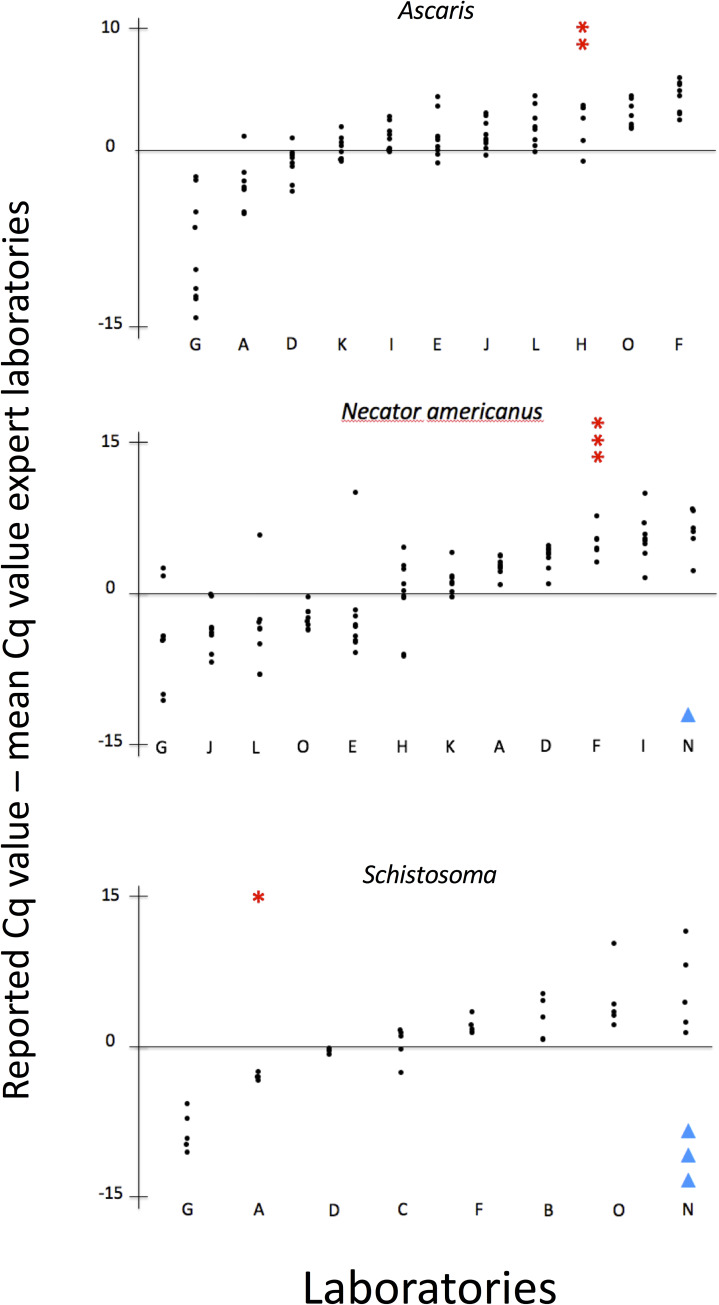
The variation in the reported Cq-values across the different laboratories and targets. Each dot represents the difference in Cq-value for one sample reported by one laboratory in comparison with the median Cq-values reported by the expert laboratories for that sample. These differences are shown on the Y-axis, with negative values meaning lower Cq-values compared to the median Cq-values reported across the experts, and positive values meaning higher Cq-values. The laboratories, represented by letters, are shown on the X-axis. The number of false-negative and false-positive results reported by each laboratory are shown by means of red stars and blue triangles, respectively. Laboratory M was omitted from this analysis because no positive results were reported by this laboratory.

All laboratories reported results in relative quantification units, i.e. using a unit that reports the cycle at which the fluorescence from the qPCR reaction exceeds the background fluorescence. None of the laboratories reported in absolute quantification units. **Fig 2** illustrates the variation in Cq-values reported across the different laboratories for each target and each sample separately. Overall, there was a large variation in reported Cq values. For example, across laboratories, the variation for each of the seven *N*. *americanus* positive samples exceeded ten Cq-values. The number of FN and FP results per laboratory and target as a function of the reported Cq values are shown in **Fig 3**. Overall, there was no clear link between FP or FN and the Cq-values. Because of the limited number of positive targets for *Trichuris* and *Strongyloides*, plots are not shown for these helminth species.

## Discussion

NAATs are increasingly used for the detection and quantification of *Schistosoma* and STHs, including *Strongyloides*, but an EQAS, a key component for an overall quality management system, is lacking. To our knowledge, we piloted the first international EQAS for the DNA-based detection of these parasitic worms. In the current study, a total of 15 clinical and research laboratories, representing both non-endemic and endemic countries, participated in this EQAS for NAATs targeting *Schistosoma* and STH, including *Strongyloides*.

### The HEMQAS panel was stable over the course of the study

For both the DNA and the stool panels, there were only small differences in quantitative results reported among the expert laboratories during the validation of the panel and the pilot study, which was completed approximately 6 months later. These findings demonstrate that both panels were stable over the course of the study, and that discrepancies were not due to degradation of DNA during long-term storage. These results correspond with previous reports that showed that eggs from STHs in stool could be well preserved in ethanol over a one-year period for subsequent examination by NAATs [25]. However, for one stool sample, there were discrepant results between the validation round and the pilot study. This sample was validated as a *Trichuris* positive sample, i.e., found positive by all expert laboratories during the validation of the panel, but found negative by three of the expert laboratories in the pilot study. Furthermore, this sample was also found negative by five of the other participating laboratories. Retrospectively, the amount of *Trichuris* DNA in this stool sample was rather low (median reported Cq-value of 34.8), comparable to that of the educational *Trichuris* samples (range median reported Cq-values 30.8 to 35.4) and probably close to the limit of detection (LOD) of most NAATs. In general, the probability of reporting a FN increases when the target DNA concentration approaches the LOD, which probably explains these FN results, although the LODs of the different NAATs are unknown. In cases where fewer than 50% of the participating laboratories report the expected positive result, it is common practice in EQAS organized by SKML to change the status of that target from positive into educational. However, this was not done during current pilot study. Knowledge of the absolute target concentrations of each sample and the LODs of each NAAT would allow for better interpretation of such cases (see also below).

### Participation in HEMQAS enables identification of flaws in diagnostic performance

Overall, the diagnostic performance of most laboratories was good. However, a substantial number of FPs and FNs were reported. The majority of reported FPs in our study involved *Strongyloides* reported by a single laboratory. Remarkably, all FP *Strongyloides* results reported by this single laboratory were *Ascaris* positive stool and DNA samples. Hence, one of the possible explanations is that there was a cross-reaction between *Strongyloides* and *Ascaris* in the qPCR protocols of this laboratory, e.g. due to unspecific primers and/or fluorescence cross-over between channels. Another possibility is that *Strongyloides* negative samples were contaminated during laboratory manipulations and/or non-specific curves were considered as specific DNA amplification, e.g. because of fluorescent crosstalk from other channels. The fact that *Strongyloides* FP test results were reported both in the stool and the DNA panel emphasizes the need for including both stool and DNA samples in an EQAS for helminths. Indeed, without including DNA samples, the laboratory reporting these FP results could have counter-argued that their FP test results in the stool panel were actually true positive samples that were missed by the expert laboratories due to a difference in DNA extraction protocols. By including a panel of DNA samples derived from parasites, this possible counterargument can therefore be rejected. Three FP *Schistosoma* test results were reported by a single laboratory and included both stool and DNA samples (the FP reported DNA samples were a true positive *N*. *americanus* and *Strongyloides* sample). These were likely due to contamination during laboratory manipulations or cross-reactivity with other targets.

Over half of the FN test results were reported by a single laboratory (laboratory M), which did not report a single positive result for any of the targets in the pilot EQAS. The reasons for this remain unclear, as we validated the stability of the panel and this laboratory confirmed the use of positive and negative controls.

Although educational samples are more challenging to interpret, additional information on diagnostic performance can be obtained from them. For example, the second educational *Strongyloides* DNA sample (DNA2, see **Table 3**) which was a hundred-fold dilution of the positive *Strongyloides* DNA sample (DNA1, see **Table 3**) was validated as educational because it tested positive in only 4 of 5 expert laboratories. Nevertheless, in the pilot study, it was tested positive in the majority of participating laboratories with a median Cq-value of 34.6, indicating that this was probably close to the LOD of most assays used. One of the educational *Strongyloides* stool samples tested positive by the majority of laboratories as well. The inclusion of more of these challenging samples and more participating laboratories in future HEMQAS will reveal further details about the association between used test protocols and test outcome.

### The lack of information on both the absolute quantity of targets in the HEMQAS panel and LOD of NAATs leads to ambiguous interpretations

Besides above-mentioned FP and FN test results, FN test results were reported for *Schistosoma* (n = 1), *Ascaris* (n = 2) and *N*. *americanus* (n = 3). It remains for the laboratories to interpret these results, but this currently remains a challenge. These FNs might be explained by less efficient DNA extraction procedures and/or a poorly performing NAAT. Knowledge of both the absolute target concentrations in the panel and of the LOD of the NAAT would allow for better interpretation of such cases, as well as the interpretation of the educational samples. In other words, it would allow to decide that a FN is missed because of the LOD of a NAAT or because of another error. However, this strategy is currently complicated by the absence of a unit that expresses qPCR results as absolute DNA concentration of the targets and that allows these absolute concentrations to be compared if results are obtained from different assays/laboratories [16,17]. Such a unit is required to compare LODs and quantitative HEMQAS values among laboratories [17].

### The high diversity in DNA extraction and NAAT protocols calls for standardization

The current study highlights the diversity of both DNA extraction and the NAAT protocols used for the detection and quantification of helminths in stool. Due to the design of the pilot study we did not explore differences in protocols that may explain the variation in diagnostic performance across laboratories. For this, a larger number of participating laboratories and samples will be needed to address these issues. In addition, given the plethora of protocols, more standardization in both DNA extraction and NAAT protocols should be encouraged. However, it is unlikely that laboratories will completely relinquish their in-house protocols, which required important investments to develop. A level of standardization that is feasible and increases overall diagnostic performance will include (i) methodological steps that have been proven to improve the performance of NAATs, e.g. by improving DNA extraction efficiency through mechanical lysis of samples (bead beating) [25,26], (ii) the use of positive, negative and inhibition controls, (iii) regular participation in an EQAS, (iv) assessment of the LOD of the NAATs and (v) reporting the quantity of DNA in absolute units.

### Challenges for the use of NAAT in a programmatic setting

In clinical laboratories, qualitative NAAT test results for helminth infections, which indicate presence or absence of infection in an individual patient, are sufficient to make decisions on the initiation or success of therapy. However, there are still important obstacles that impede implementation of NAATs in large-scale deworming programs [16,17]. In these programs, both prevalence and intensity of infections at the population level are important indicators that guide decision making [27]. This information is currently provided by assessing the fecal egg counts by means of microscopic methods, of which the Kato-Katz is the most widely used. The intensity of infection is often further classified as low, moderate or heavy [28]. NAATs have been considered and suggested as potential alternatives to these microscopic tools, due to their ability to detect very low levels of egg excretion [17]. However, the public health community is currently not ready to use NAATs in control programs. The obstacles that are currently hampering the use of qPCR are low throughput, high cost, lack of standardization and the absence of a quality assurance scheme. Furthermore, qPCR outcomes will need to be readily translated to program decision algorithms.

### Limitations and recommendations for future HEMQAS

Although they also cause significant health burdens in some areas, we did not include targets such as *Ancylostoma* (including *A*. *duodenale* and the zoonotic *A*. *ceylanicum*) and other *Schistosoma* species such as *S*. *haematobium* and *S*. *japonicum*, although these helminths also cause significant health burdens in some areas [2,8]. Future HEMQAS rounds should include these species. Moreover, an important improvement would be the inclusion of *Trichuris* DNA in the DNA panel to better understand the somewhat poor results in our study for this target in stool samples. In the present study, clinical samples were obtained from a single site (Jimma, Ethiopia). In order to capture (possible) more diverse species and strains, samples from geographically more dispersed regions should be included in future HEMQAS. To gain more insights into discrepancies in diagnostic performance across laboratories, it would also be informative to ask participating laboratories to develop a standard curve on a common target DNA material. From 2019 onwards, SKML provides a HEMQAS open to laboratories from all over the world.

### Conclusion

We showed the technical feasibility of preparing homogeneous and stable stool and DNA samples for an international EQAS for the NAAT of *Schistosoma* and STHs, including *Strongyloides*. We documented a clear benefit of participating in a HEMQAS, as laboratories were able to identify weaknesses and/or to confirm the performance of their NAATs. In contrast to other organizations providing panels for the molecular detection of pathogens in an EQAS, which commonly distribute only purified DNA in an artificial matrix, we demonstrated the necessity of sending both stool and DNA samples, enabling laboratories to evaluate the entire laboratory procedure including the important DNA extraction step. Furthermore, we showed a high technological diversity in both DNA extraction and NAATs protocols, which underscores the urgent need for such an EQAS. HEMQAS should be implemented as an integral part of the overall standardization and quality management in the field of molecular detection of helminths. There is a need for a minimal set of recommendations to improve both the diagnostic performance of protocols, including but not limited to the assessment of the LOD, reporting DNA concentration in absolute and universal units, and participating in an EQAS. Further research should aim to identify factors that explain poor performance of NAATs and how the NAATs can best be implemented in large-scale deworming programs.

## Supporting information

S1 File Summary manual and extended manual of the QBase submission tool. (PDF)

S2 File Instructions accompanying the panel on how to remove ethanol from the stool samples. (PDF)

S3 File The target validation of the stool and DNA panel by expert laboratories. (PDF)

S4 File Example of a HEMQAS report. (PDF)
