# Supplementary material for: First international external quality assessment scheme of nucleic acid amplification tests for the detection of Schistosoma and soil-transmitted helminths, including Strongyloides: A pilot study
Source: PLoS Negl Trop Dis. 2020 Jun 16;14(6):e0008231. doi: 10.1371/journal.pntd.0008231 (PMC7319349; doi:10.1371/journal.pntd.0008231)
Supplement: S1 File — (PDF) [file pntd.0008231.s001.pdf]

S1 File. SUMMARY MANUAL AND EXTENDED MANUAL OF THE QBASE SUBMISSION TOOL

QBase manual

1) Download the “QBase Portable software at the SKML website:
   https://skml.nl/en/home?op=setLanguage;language=English
   (via the services / Qbase scroll down menu, marked by the red arrows)

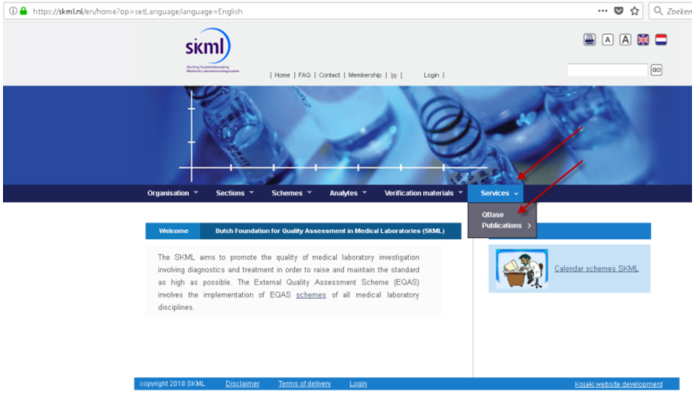

   The download can be started on the next appearing page (see picture below). Leave the Number box open (do not fill in anything), fill in “fruit” in the “Username” box and “mango” in the Password box.

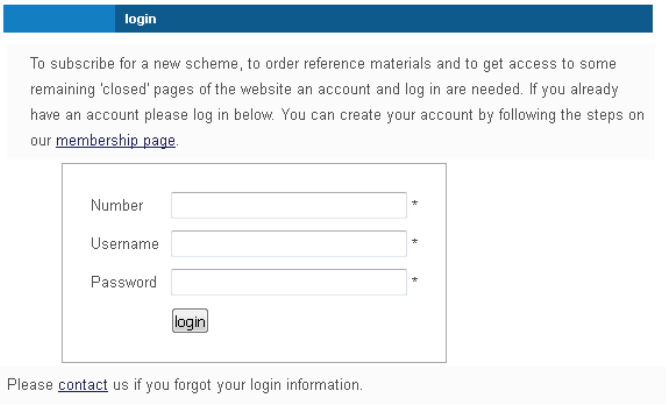

   The Qbase download can now be started by clicking on the “login” button. Qbase can be saved and started from your download directory (or any other directory) without further installation of the programme.

2) After the Qbase programme is started the following login screen will appear:

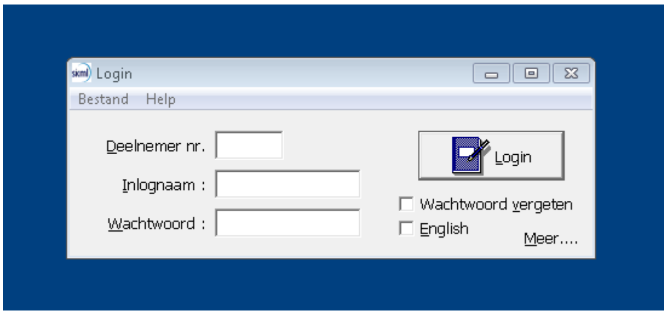

   Check the box “English” and continue in English language

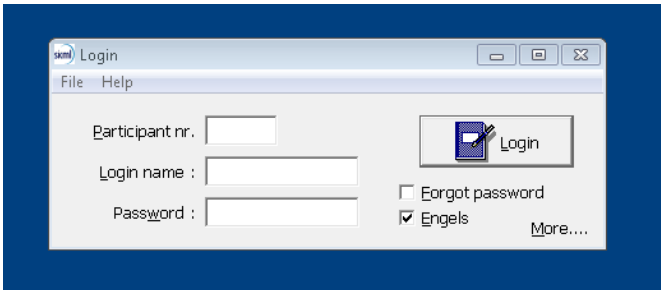

You can now login by using your unique participant number, login name and password that you have received from the SKML office.

3) After login the following Qbase-screen will appear. Please select the Pilot HEMQAS (red arrow) by double clicking on it.

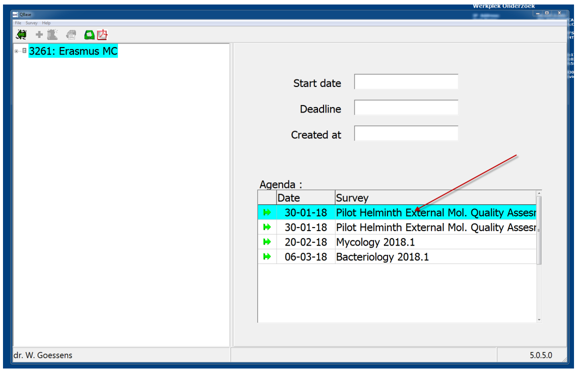

4) Select distribution 2018.1 to report the results for the stool samples (red arrow) or 2018.2 to report the results for the DNA samples.

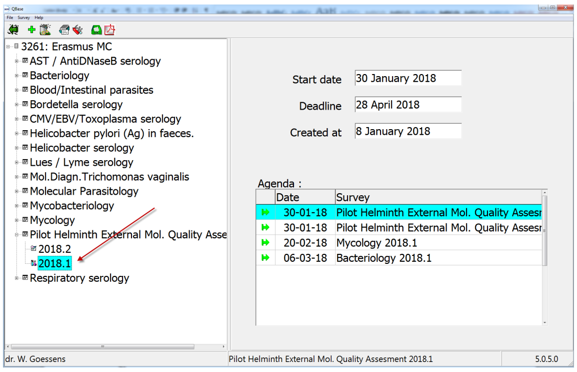

5) After the first login, Qbase will open the “settings” page. Please select the all the PCR assays that are performed in your laboratory (select one by one the PCR assay and press the green “V” at the top of the page; indicated by the red arrows in the picture below).

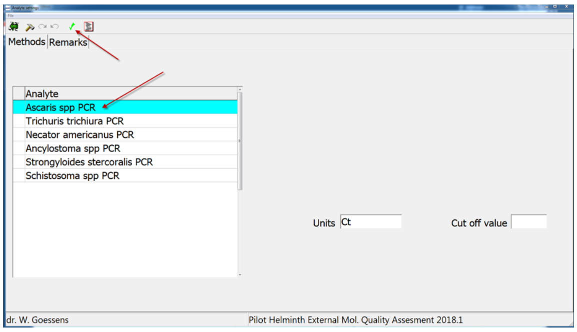

Continue until all PCR assays that are performed in your laboratory are marked by a green "V". In the example below the participant performs 4 of the 6 listed PCR assays. Your selection can be saved by pressing the 'blue curved arrow button' op the top of the page.

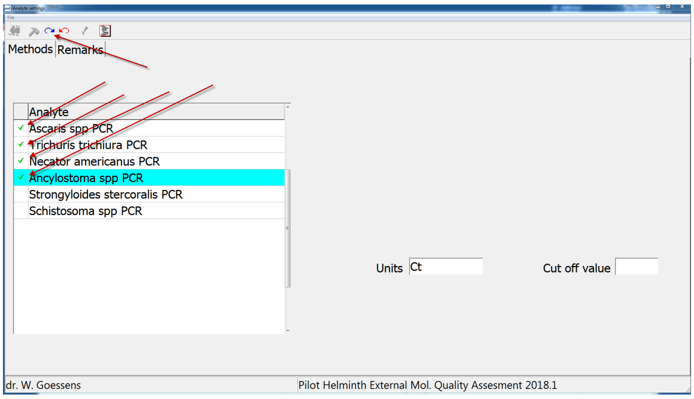

When all PCR assays that are performed have been are properly marked and seved, leve the setting page by pressing the green arrow (top left of page).

6) Qbase will now open the results report pages.

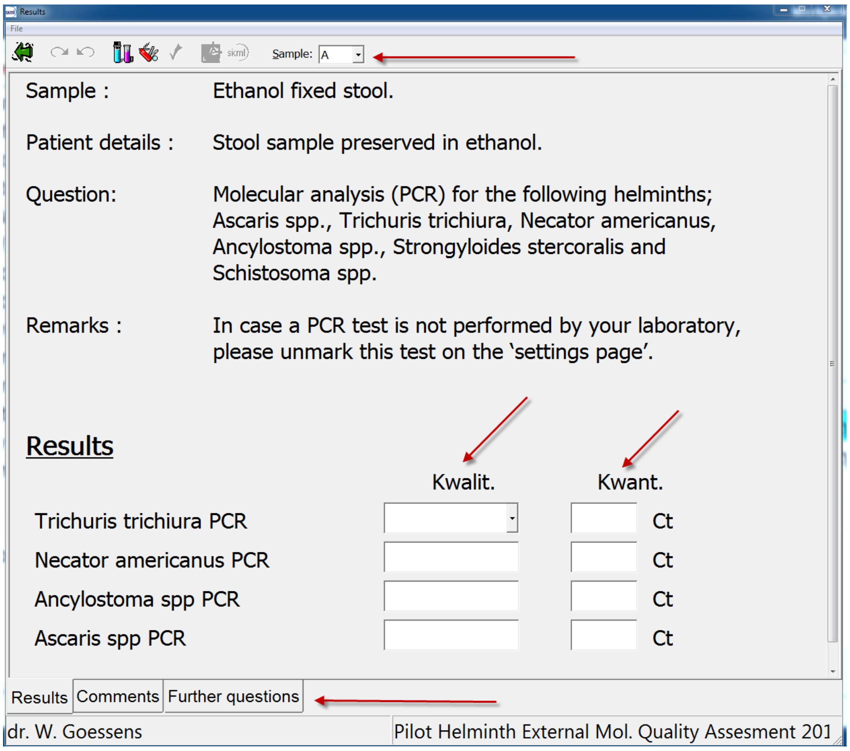

For each sample results should reported on a distinct page (example above is the report page for material A; switch to next sample by drop down menu at the top of the page). Please report for each PCR target in the "Kwalit" box, whether the test result is "Positive", Negative, Inhibited or failed (because of another reason than DNA amplification inhibition). If the test results was positive, please report threshold cycle (Ct) in the "Kwant."box.

7) Finally answer the additional questions by clicking on "Further questions".

Stichting Kwaliteitsbewaking
Medische Laboratoriumdiagnostiek

QBase

Instruction Manual

Version 4.1

v 4.1.0.1. November 2005

Contents

0. Introduction 6
0.1. QBase history 6
0.2. Reading instructions 6
0.3. SKML definitions 7

1. Starting QBase 10
1.1. Starting 10
1.2. Logging in to the central database 10
1.2.1. Logging in; entering the wrong login name or password 11
1.2.2. Logging in options 11
1.2.3. Forgotten password 12
1.3. Altering the login data 12
1.3.1. Altering the login name 12
1.3.2. Altering the login password 13
1.4. Automatic notifications when starting QBase 13
1.5. Connecting to the TEST database 13
1.6. New versions of QBase 14
1.6.1. Downloading automatically 14
1.6.2. Blocking an old version 15

2. The QBase main window 16
2.1. The tool bar 16
2.2. The menu bar 17
2.3. The context-sensitive mouse menu 17
2.4. The status bar 18
2.5. Functions in the main window 18

3. Program settings 20
3.1. The toolbar 20
3.2. The menu bar 20
3.3. Settings 20
3.4. Proxy 21
3.5. Adobe files 21

4. Downloading an agenda 23

5. SKML messages 24
5.1. The tool bar 24
5.2. The menu bar 24
5.3. The context-sensitive mouse menu 24
5.4. The status bar 25
5.5. Writing a message 25
5.6. Reading a message 25

6. Reports 26

7. Data entry form 27

8. Clusters 28

8.1. Creating a (new) cluster 28
8.1.1. There is no history 28
8.1.2. There is a history, but nothing can be selected 28
8.1.3. There is a history from which to make a selection 29
8.1.4. Entering the sample dates 29
8.1.5. When a new cluster has been created 30
8.2. Deleting a cluster 30
8.3. Changing the name of a cluster 31
8.4. The history of a cluster 31
8.5. Moving a cluster 31

9. Cluster settings 32

9.1. The tool bar 32
9.2. The menu bar 32
9.3. The context-sensitive mouse menu 33
9.4. The status bar 33
9.5. Creating cluster settings 33
9.5.1. Altering cluster settings; automatic settings 34
9.6. Cluster settings 34
9.6.1. Methods (External and Combi) 34
9.6.2. Internal control materials (only Combi schemes) 35
9.6.2.1. Selecting an internal control 36
9.6.2.2. The tool bar 36
9.6.2.3. The menu bar 36
9.6.2.4. Selecting an internal control 36
9.6.2.5. Exchanging internal controls 38
9.6.3. Order of internal control materials 39
9.6.3.1. Changing the order of the internal control materials 39
9.6.4. Adjusting user limited values 39
9.7. Summary of the cluster properties 40
9.8. Remarks 40

10. Results entry 41

10.1. The tool bar 41
10.2. The menu bar 41
10.3. The context-sensitive mouse menu 42
10.4. The status bar 42
10.5. Entering results 42
10.5.1. Plausibility check of the entered results 43
10.5.2. Validating the results 44
10.5.3. Remarks 44

10.6. Result entry window, type External ... 45
10.7. Result entry window, type Combi ... 45
10.7.1. Result entry per sample ... 46
10.7.2. Result entry per analyte ... 46
10.7.3. Why is the result field deactivated and entry not possible? ... 47
10.7.4. Entering analysis dates for INTERNAL control material ... 47
10.8. Importing results ... 47
10.8.1. Import function diagnosis ... 49
10.9. Downloading a report ... 50

11. Report management ... 51

11.1. Availablity of reports ... 51
11.2. Getting reports ... 51
11.3. The report management window ... 52
11.3.1. The tool bar ... 52
11.3.2. The menu bar ... 52
11.3.3. The context-sensitive mouse menu ... 53
11.3.4. De status bar ... 53
11.4. Reports; E-mail with hyperlinks ... 53

12. Statistics ... 54

12.1. Examples of summaries ... 55
12.1.1. Method settings ... 55
12.1.2. Results ... 55
12.1.3. Reports ... 56
12.1.4. Interim report ... 57
12.1.5. Data entry form ... 57
12.1.6. Statistics ... 58
12.1.7. Detailed statistics ... 58
12.1.8. Time diagram ... 58
12.1.9. Histograms ... 59
12.1.10. Difference plots ... 60

13. Appendix ... 61

13.1. Structure of the QBase import file ... 61
13.1.1. Title ... 61
13.1.2. Comment line ... 62
13.1.3. Specimen line ... 62
13.1.4. External sample line ... 62
13.1.5. Result line ... 62
13.1.6. Internal sample line ... 63

14. Installation ... 64

14.1. New version of QBase ... 66
14.1.1. Updating automatically ... 66
14.2. Information for system administrators ... 67

15. Reference words 69

0. Introduction

This instruction manual explains how to use **QBase**, version 4.
In this instruction manual, all the QBase functions are explained in detail using two types of scheme:

- **Combi**: this type is characterized by
  - a A separate closing date for each sample.
  - b The possibility to input internal results.
- **External**: all other presentation forms.

The terms “External” and “Combi” are used in this instruction manual to refer to these two types of scheme.
Schemes with more specific operations are all based on the “External” type with the more specific details of the schemes concerned included in them.
In order to avoid issuing all the participants with a very thick instruction manual which, due to its size, is not very inviting to use or read, it has been decided to produce an instruction manual and a number of very brief instructions which fit on one sheet of paper. These documents can be downloaded from the “Downloads” page on the SKML website (http://www.skml.nl).

0.1. QBase history

QBase history:

QBase1	1989 - 1991	(MS-DOS)
QBase2	1991 - 1999	(Windows 3.1)
QBase3	1999 - 2004	(Windows 95)
QBaseWWW	2002 - Present	
QBase4	2004 - Present	(Windows 98 and later releases)

0.2. Reading instructions

The following has been assumed in this instruction manual:

- When referring to QBase, it is assumed that this is version 4.
- QBase only runs on Windows 98 and later releases of Windows. It does not work correctly on some Windows 98 systems.
- Sections of text which are marked by an exclamation mark in the margin deserve extra attention.
- :The default font for **normal text** in this document is Arial, size 11.
- **Comments** will be given in italics, size 10.
  <u>Comment:</u>

The heading and text of a comment.

- QBase has been designed in such a way that the buttons in the tool bar and the menu options are only visible when the function they represent is applicable. There are some functions which are specific to only one type of scheme and these will, therefore, only be visible for that specific type of scheme.
- **References** will be given in bold italics, size 10. ***See chapter…***

- When the instructions refer to text which is shown in the window being described, then this will be indicated in bold, size 10. For example, **Agenda** in the main QBase window or **QBase** in the title bar of the main window.
- :menu options**Menu options** will be preceded by the words “menu option:"
  Many menu options are also available by shortcut keys, such as **CTRL+N** in the File menu. These shortcut keys are self-explanatory and will not be explained in this document.
- buttons**Buttons** are represented by a picture as much as possible in this document (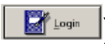). Most of the buttons within QBase include a ”hint”; in other words, when the mouse pointer is placed over the button, a yellow text bar containing an explanation of the button’s function will appear almost immediately. This yellow text bar is called a “ScreenTip” in Windows.
- mouse button**Mouse button**; when a right mouse button or a left mouse button is referred to, this applies to a right-handed mouse. The mouse buttons will be reversed for a left-handed mouse.

0.3. SKML definitions

A number of terms which have a specific meaning within SKML are used in this instruction manual. The most important of these terms are explained in this chapter.

Institute
A participant’s institution or hospital.

Participant
A laboratory (a group of one or more departments or work units which are closely related organizationally, geographically and logistically) which subscribes to and participates in the external quality control of SKML.
A participant can participate in one or more sections of the quality control, in the entire program or only in a part of it, i.e. participate in some schemes of the section program and not in others.
Every participant has its own unique number in the SKML database.

Participation
Subscribing, until further notice, to one or more section programs or schemes (covering all the surveys in a scheme) of SKML. Subscription, therefore, applies to a whole scheme and not to individual surveys.
The subscription can be stopped or altered before 1st December and this will take effect from the start of the next calendar year.

Contact person
A person who has access, via a combination of a login name and a password, to the scheme for which the person concerned is registered as the contact person. In order to have access to a scheme, this person must be known as the contact person for “samples”, “reports” or must be known as the “quality officer”.

Scheme
A number of surveys (one or more) which are technically related and which participants can participate in.

Survey
A simultaneously distributed number of samples which are sent to each participant who participates in a scheme.
It concerns the entire process, which consists of the formulation of a problem and the choice, composition, production and distribution of one or more samples which must be examined by the participant, up to the processing of the results returned by the participants, the discussion, evaluation, etc.

Cluster
The grouping of results within one survey of a scheme with all the accompanying settings (measurement environment, method, analyser).

Cluster chain
Cluster chains are defined within schemes in which unique combinations of surveys (clusters) are grouped.
Comment:
The terms cluster and cluster chain are only important if results are entered for more than one analysis environment within one survey. These terms only serve as an aid for grouping results which belong together with their settings.

Sample
An examination specimen which must be examined by a participant as part of a survey.

Production centre
A laboratory centre which, on behalf of SKML, produces the samples of a survey by order of the selection committee or distributes them to the participants by order of the scheme coordinator or the survey distributor.

Scheme coordinator
A selection committee member (or another technical expert) who has integral responsibility from the beginning to the end for all aspects (composition, logistics, etc.) of a certain scheme from the program of a section.
This person formulates the scheme, checks the quality of the (surveys in the) scheme, coordinates the logistics, answers any technical questions, takes care of the evaluation, etc. This person is supported by the Central Facilities Service.
This person is also the contact person for questions from participants and the Central Facilities Service concerning the scheme. It is up to the scheme coordinator whether he/she wishes to answer the questions him/herself or have them answered by a third party.

Survey distributor
A selection committee member (or another technical expert) who has integral responsibility from the beginning to the end for all aspects (composition, logistics, etc.) of a certain survey of a scheme in a section.
This person formulates the survey, checks the quality of the survey, coordinates the logistics, answers any technical questions, takes care of the evaluation, etc. This person is supported by the Central Facilities Service.
This person is also the contact person for questions from participants and the Central Facilities Service concerning the survey. It is up to the survey distributor whether

he/she wishes to answer the questions him/herself or have them answered by a third party.

Result
The result of the examination of a sample by a participant.
This can consist of:

- The information provided by the participant concerning the way in which the result is achieved and/or the resources used to obtain the result.
- The result of the technical analysis.
- The professional (clinical) interpretation of the result from the technical analysis.
- A combination of the possibilities given above.
- *Provisional result*
  The result as intended by the scheme coordinator. This is often produced by one or more reference laboratories or, in other words, it is “the intended correct answer”.
- *Definite result*
  The result based on the provisional result and the results from the participants. Sometimes, despite all the precautions, the provisional result must be altered based on the results from the participants.
- *Score*
  The quantitative assessment of the result obtained by a participant according to the guidelines determined by a Section.

Clinical interpretation
A technical analysis is always returned by the participants in a scheme. Furthermore, a professional (clinical) interpretation of the results of the participants’ technical analysis, against the background of the special circumstances of the (fictional) patient, is also requested. Sometimes it is requested at the same time as the technical analysis, sometimes only at a later date.

Questionnaire/Detailed questionnaire
Structured questions relating to a scheme:
This can be of either a technical or clinical nature.

Evaluation
The report of a survey. It contains one or more components:

- A representation of the quality controls relating to the samples of a survey, as well as a conclusion concerning the technical quality of the samples which have been sent (satisfactory/unsatisfactory).
- A cumulative representation of the results from the participants.
- An assessment of the results from the participants as a group.
- A full discussion of the results of a survey.
- Technical advice on how the participants can improve their results.
- Etc., etc.

1. Starting QBase

This chapter explains how to start QBase.
A detailed explanation will also be given of special functions which, although they can be performed from the main window of QBase, are of particular importance and so deserve a separate explanation. These functions are:

- Starting QBase.
- Automatic notifications when starting QBase (see ***1.4 Automatic notifications when starting QBase***).

Connecting to the test database (see ***1.5 Connecting to the TEST database***)
Updating to a new version of QBase (see ***1.6 New versions of QBase***)

1.1. Starting

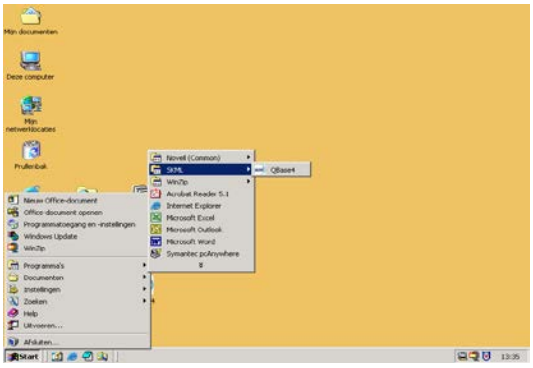

To start QBase, click the start button in the bottom left-hand corner of the Windows® task bar and then click **Programs – SKML – QBase4** .
Depending on the settings, QBase will either display the Login window or log in automatically and display the main window (***see chapter 2 and further***).

1.2. Logging in to the central database

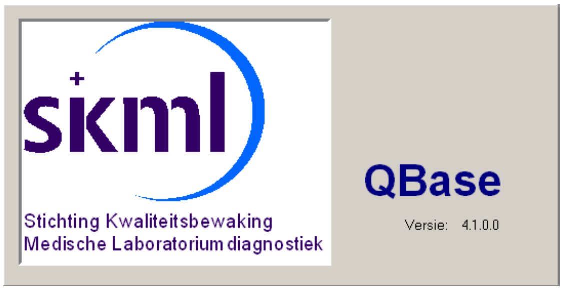

A splash screen will be displayed for a short time when QBase is started. This splash screen displays the SKML logo, the QBase name and the complete version number of the program. This will then be followed by the Login window.

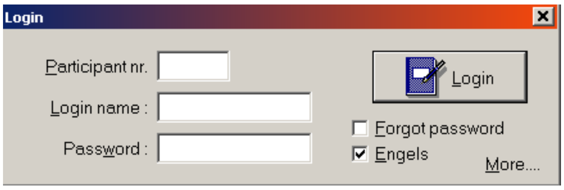

Enter your participant number in the **Participant nr**. field, your login name in the **Login name** field and your password in the **Password** field and then click 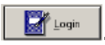.

Comment:
QBase always remembers the last information entered (except the password).

1.2.1. Logging in; entering the wrong login name or password

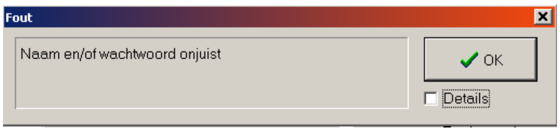

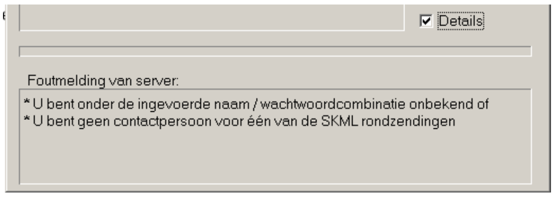

QBase will display an error message if the combination of the information which has been entered into the three fields is incorrect.

Selecting **Details** will give more detailed information, if available (only available in Dutch).

1.2.2. Logging in options

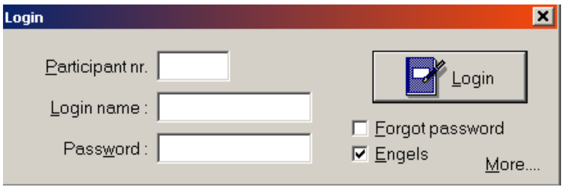

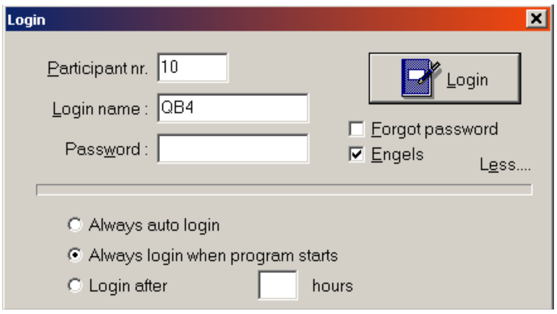

Selecting the **Forgot password** box will open a new window (see chapter ***1.2.3***).

Selecting **English** will change the language of QBase to English.
Deselecting this checkbox will change the language back to Dutch.

Clicking **More** will give access to extra login options.
The choices are self-explanatory.
Clicking **Less** will hide the extra login options.

The following concerns the option **Always login when program starts**:
QBase remembers all the data of the participant who last logged in and will log in automatically as that participant the next time it is started. This can cause problems for participating laboratories where a number of people work with QBase and each person is responsible for a part of the schemes. In that case, not all of the schemes of a certain person will be displayed.
You could set the **Login after … hours** option to, for example, 8 hours. During one working day QBase logs in automatically every time as the same person. After 8 hours, though, it will be necessary to log in again.

1.2.3. Forgotten password

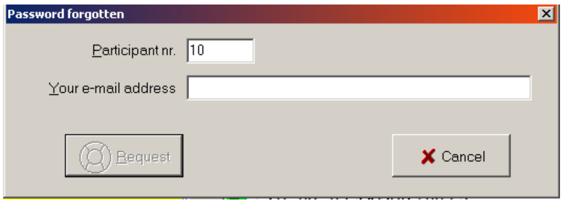

Enter the participant number and e-mail address in this window. The button will become active as soon as a character is entered after the @ sign of the e-mail address. After this button has been clicked, QBase will check whether the e-mail address which has been entered corresponds to the participant number in the database. If it does, then an e-mail which contains the login data will be sent immediately to the e-mail address.

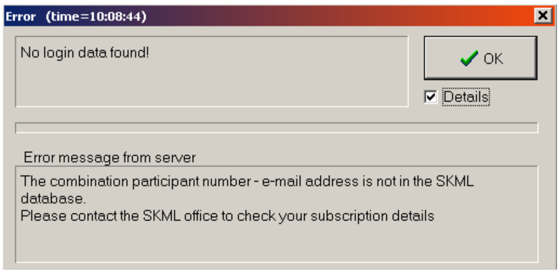

If the e-mail address does not correspond to the participant number in the database, QBase will display the window opposite. Selecting **Details** will display the bottom of the window, which contains an explanation of the error found.

1.3. Altering the login data

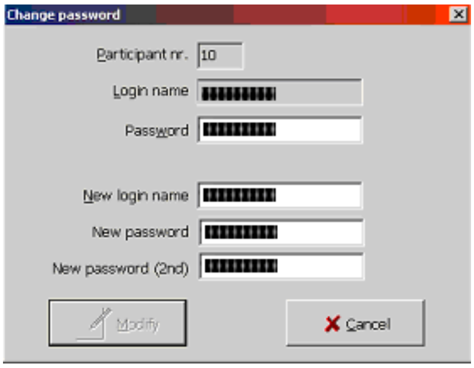

Click the menu option **Change password** in the **File** menu. The window opposite will then be displayed.
The old password must be entered in the **Password** field.

The rules for altering the password are:

- The login name and password must be at least 5 characters long.
- The login name and password must not be the same.
- Both new passwords entered must be identical.

QBase checks the data whilst it is being entered. If invalid information is entered, then this will be indicated by a red after the input field when exiting the field.

1.3.1. Altering the login name

It is sufficient to enter a new name in order to alter the login name. Clicking will then save the new name in the database.

1.3.2. Altering the login password

To alter the password, the new password must be entered twice. This is to check whether the password has been entered correctly, because it is only displayed by asterisks (*).

1.4. Automatic notifications when starting QBase

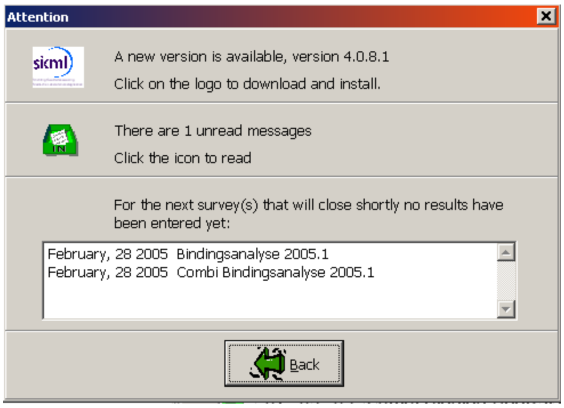

The example shown opposite displays the most comprehensive possibility:

- Notification that a new version of QBase is available.
- Notification that there is one or more unread SKML messages.
- Notification that no results have yet been entered for a survey which is shortly going to close.

The notifications are self-explanatory and will not be explained further.

1.5. Connecting to the TEST database

With QBase, the participants have the possibility to connect to the development and test database (D&T database) of SKML.
This D&T database is regularly updated with data from all the participants from the production database and serves a number of objectives:

- The development of new versions of QBase.
- An environment which participants can log in to for training purposes.

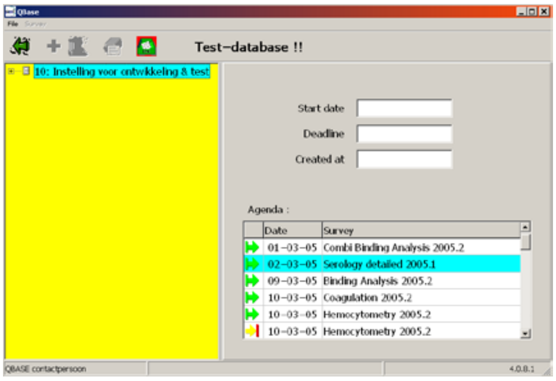

When clicking the menu option **Test database** in the **File** menu, the Login window is displayed again, because it is necessary to log in again for the other database.
The same combination of login name/password can be used as for the production database.
The window opposite will be displayed when the user has logged in successfully.

The left-hand panel of the window has a bright yellow background and **Test-database !!** is prominently displayed at the top of the window.
This is to inform the participant that he or she is not connected to the real database, but to the D&T database.
Comments:

- *Since this environment is still being developed, the availability of this environment is never guaranteed. If the database is not available, then QBase will display the error message "Database not available".*
- *Since, at various times and for various reasons, a fresh copy of the production database is placed on the D&T database, whilst at other times this will not be the case, the participant must be prepared to encounter a situation which is different to what he or she is used to in the production environment and that try-outs can suddenly disappear after a couple of days. Please contact the SKML office if you are planning to set up a training course using this database.*
- *The start option can be used to make QBase log in to a certain environment. For example: "c:\program files\skml\qbase4.exe /p" will log in to the production database "c:\program files\skml\qbase4.exe /t" will log in to the development and test database*

1.6. New versions of QBase

1.6.1. Downloading automatically

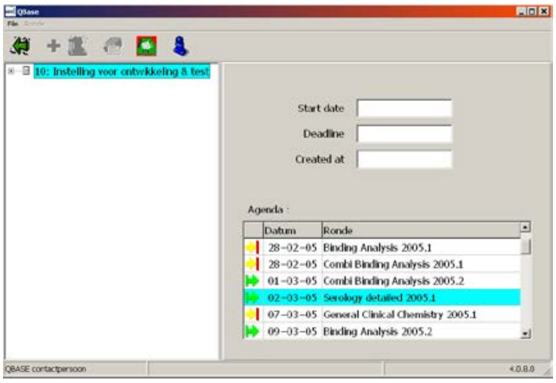

If, after logging in to QBase, the 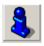 button is visible in the tool bar, a new version of the program is available and can probably be downloaded automatically.

Click the 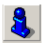 button to start the download and installation of the new version.

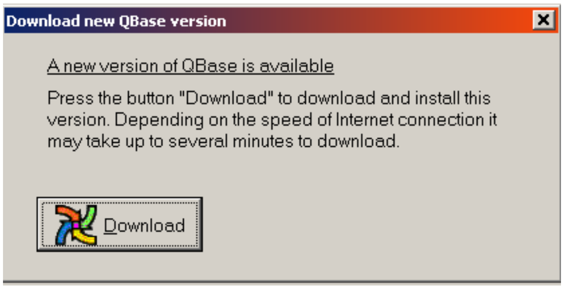

Once the 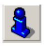 button has been clicked, QBase will first check whether the user of the PC has sufficient privileges on the PC to copy files. If so, the 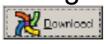 button will be displayed. Clicking this button will start the download.

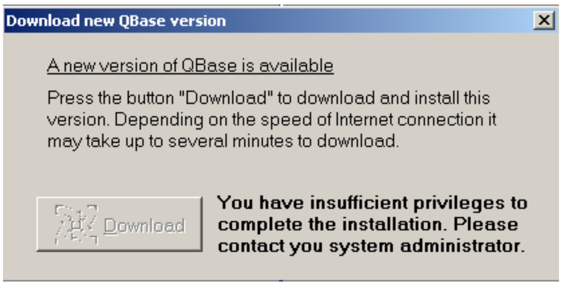

If the system administrator (of the department or hospital) has limited the individual user privileges to such an extent that it is no longer possible to copy files, then it is only possible for QBase to be updated by the system administrator.
If the program has been successfully downloaded and installed, QBase will start again and display the Login window.

<u>Comment:</u>
If QBase has determined that the user can download and install a new version, then this is not a guarantee that the installation will be successful. For example, it must also be possible to start a program via another program on the PC concerned.

QBase is updated as follows:

- *The user sees that a new version is available and clicks the button and then the button.*
- *QBase will download the file for the new version and save it under the name "QBase_new.exe" in the same folder as where the program is currently located.*
- *QBase will start Rename_QB and then close.*
- *Rename_QB will remove QBase.exe.*
- *Rename_QB will rename QBase_new.exe as QBase4.exe.*
- *Rename_QB will start QBase.exe and then close.*

If it now appears that nothing is happening after the button has been clicked, or the update procedure is not successfully completed (the login window should be displayed again), please contact the CFB (Central Facility Bureau) of SKML (☏+31 24 361 66 37).

1.6.2. Blocking an old version

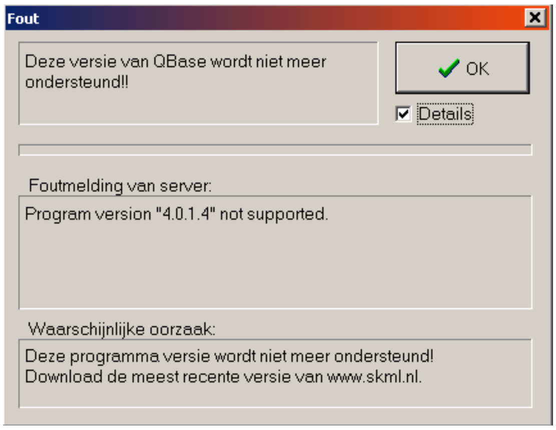

If a new version of QBase becomes available, the old version will (usually) still be supported; users of the old version will only miss the new functionality of the latest version.
Participants are not obliged to install the latest version when it becomes available.

However, it may be necessary for SKML to stop supporting an older version at some time in the future.
If a participant then tries to log in to the database using the old version, it will be blocked and QBase will only give the possibility to install the new version before QBase can be used again.
SKML will send a notification e-mail before an older version is blocked.

2. The QBase main window

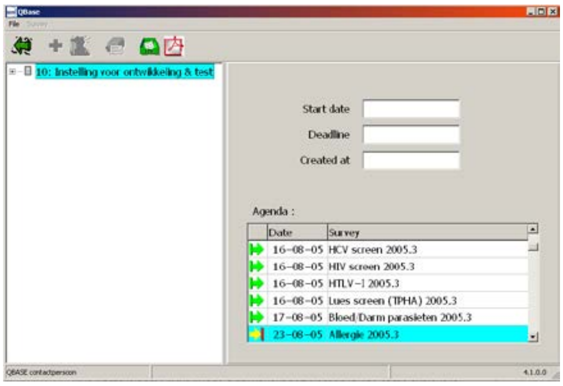

QBase will display the main window once the user has successfully logged in. The name of the institute is displayed on the left-hand side and there is an information panel on the right-hand side.
The **Start date**, **Deadline** and **Created at** fields will only contain information after a scheme has been selected.
A window is displayed below the **Agenda** heading which displays up-to-date, chronological information of the active schemes.

The icon indicates the start date of a certain survey of a scheme and the icon indicates the deadline.
If your participation in one of the surveys is recorded in the central database, a ⊞ will be displayed before the name of the institute to indicate that there is a tree which can be expanded. Clicking this icon will expand the tree. The icon will change to a ⊟, which can then be clicked again to collapse the tree again.

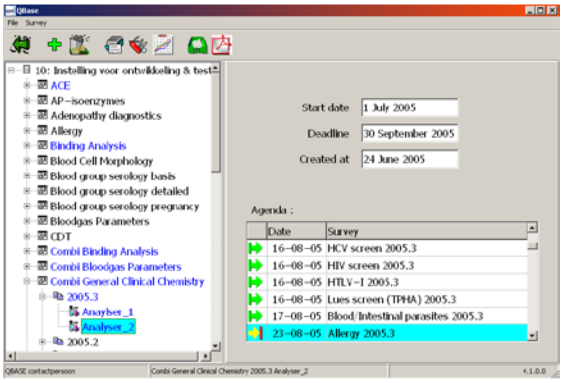

The graphic tree expanded:
In this example, the participant is participating in a number of schemes.

- indicates that the survey concerned is ready to start working with.
- indicates that a cluster has been created in the survey concerned, in which results may already have been entered.
- indicates that a number of clusters have been created in the survey concerned. These clusters will be come visible by expanding the tree. The names will also be displayed to distinguish them.

<u>*Comments:*</u>

- *Schemes where a <u>survey is currently active</u>, in other words the Start date has elapsed and the Deadline has not yet been reached, but for which no results have yet been entered, <u>are displayed in blue</u> to highlight them.*
- *It is currently possible to look back approximately one year at historical data using QBase. It is being looked at to see whether this can be made configurable in a later version of QBase.*

2.1. The tool bar

The tool bar contains a number of buttons which are context sensitive. In other words, the buttons can only be clicked if the functionality which they provide is applicable. The buttons are:

Exit	**Cluster settings (Ctrl+I)** ***(9)***
New cluster (Ctrl+N)	**Statistics (Ctrl+S)** ***(12)***
Delete cluster (Ctrl_del)	**SKML messages**[1)] ***(5)***
**Results** ***(10)***	**Reports** ***(6)***
	New version QBase: 4.1**…**

[1)] *If the background of this button is red, then the user has an unread message from SKML. See chapter* ***5 SKML messages****.*

2.2. The menu bar

Depending on the selection made in the main window, the following functions will be available under the **File** and **Survey** menus. The menus can be opened by clicking on them and then selecting the desired option or by using the shortcuts <alt>+<B> or <alt><R>.

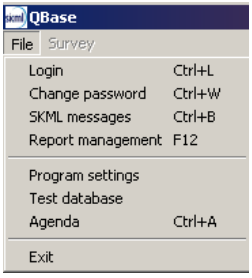

The **File** menu contains the following options:

Login	Ctrl+L	**Log in** ***(1.2)***
Change password		**Change password** ***(1.3.2)***
SKML messages	Ctrl+B	To **SKML messages** ***(5)***
Report management	F12	To **Report management (6)**
Program settings		To **Program settings** ***(3)***
Test database		Connect to the test database
Agenda	Ctrl+A	Go to **Agenda** ***(3)***
Exit		Close the program

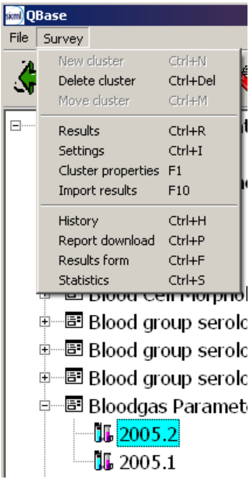

If a survey has been selected, then the **Survey** menu will be available. This menu contains the following functions:

New cluster	Ctrl+N	Create new cluster ***(8.1)***
Delete cluster	Ctrl+Del	Delete cluster ***(8.2)***
Move cluster		Move cluster ***(8.5)***
Results	Ctrl+R	Go to **Results** ***(10)***
Settings	Ctrl+I	Go to **Cluster settings** ***(9)***
Cluster properties	F1	Display cluster properties ***(9.7)***
Import results	F10	To **Import lab system** ***(10.8)***
History	Ctrl+H	Go to **History** ***(8.4)***
Report download	Ctrl+P	Create report
Results form	Ctrl+F	Create form
Statistics	Ctrl+S	Go to **Statistics** ***(12)***

2.3. The context-sensitive mouse menu

Depending on the selection, one of the menus below can be opened by right-clicking the mouse.

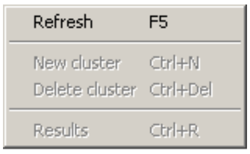

The menu opposite will be displayed if the settings or a scheme has been selected.

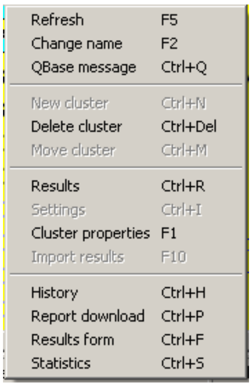

The menu opposite will be displayed if a survey has been selected:

Refresh	F5	Refresh all the data
Change name	F2	Change the name of the cluster
QBase message	Ctrl+Q	Go to **Messages**
New cluster	Ctrl+N	Create new cluster ***(8.1)***
Delete cluster	Ctrl+Del	Delete cluster ***(8.2)***
Move cluster	Ctrl+M	Move cluster ***(8.5)***
Results	Ctrl+R	Go to **Results** ***(10)***
Settings	Ctrl+I	Go to **Cluster settings** ***(9)***
Cluster properties	F1	Display cluster properties ***(9.7)***
Import results	F10	To **Import lab system** ***(10.8)***
History	Ctrl+H	Go to **History** ***(8.4)***
Report download	Ctrl+P	Create report
Results form	Ctrl+F	Create form
Statistics	Ctrl+S	Go to **Statistics** ***(12)***

2.4. The status bar

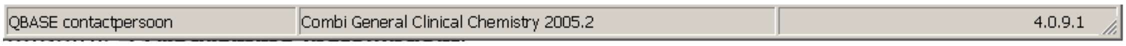

In the main window of QBase, the left-hand box of the status bar displays the name of the participant who is logged in and the right-hand box displays the QBase version number.
If a scheme or a survey has been selected, the survey name and number will be displayed in the middle box of the status bar.

2.5. Functions in the main window

All the main QBase functions can be accessed from the main window.
These will be explained in the following order:

Chapter ***3 Program settings***
- Selecting the decimal separator.
- The limit values when an outlier is observed.
- Proxy server bypass settings.

Chapter ***4 Downloading an agenda***
- Downloading an agenda.

Chapter 5 SKML messages
- Send messages to and receiving messages from SKML.

Chapter 6 Reports
- Downloading and managing reports.

Chapter ***7 Data entry*** form
- Downloading a results form.

Chapter ***8 Clusters***
- Creating and deleting clusters.
- Changing the name of a cluster.
- Viewing the history of a cluster.
- Moving a cluster.

Chapter ***9 Cluster settings***
- Entering/changing cluster settings (unit, method, analysis report, etc.).
- Adding/deleting/exchanging internal control materials.
- Setting limit values for results.
- Viewing a summary of cluster properties.
- Remarks for entering a cluster.

Chapter ***10 Results***
- Entering sample dates.
- Entering results
- Importing results from a lab system.
- A statistical summary per analyte.

Chapter ***12Statistics***
- Downloading the method settings of a cluster.
- Downloading results.
- Downloading a report.
- Downloading an interim report.
- Downloading a time plot.
- Downloading a histogram.
- Downloading difference plots.

3. Program settings

3.1. The toolbar

The tool bar contains a number of buttons which are context sensitive. In other words, the buttons can only be clicked if the functionality which they provide is applicable.
The buttons are:

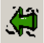 Close window

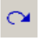 Save changes (Ctrl+S)

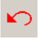 Undo

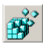 restore default

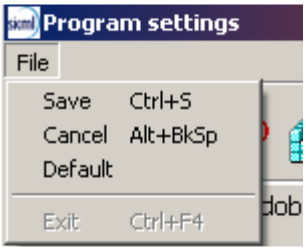

3.2. The menu bar

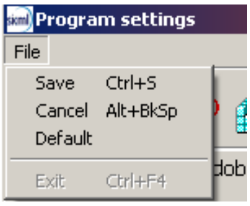

Save changes	Ctrl+S	save changes
Cancel	Alt+BkSp	undo
Default		Restore default settings
Exit	Ctrl+F4	Close windwow

3.3. Settings

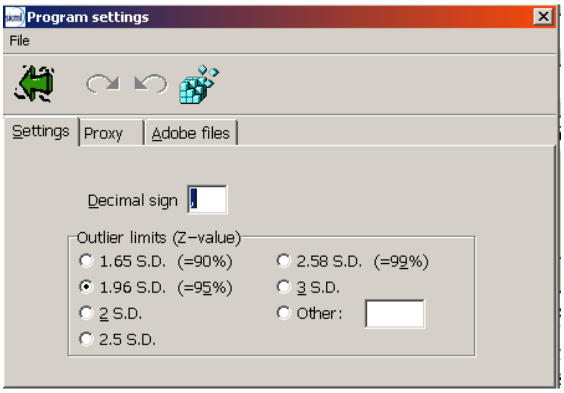

The window opposite is displayed by clicking the menu option: **Program settings**.
This window can be used to select the decimal sign and the limit value used for internal results when an outlier is observed.
By clicking the 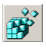 button, the default separator of the language and country settings of the PC on which QBase runs will be selected and the SD value will be set to the SKML default value of **2.58S.D**.
The 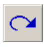 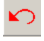 buttons will be highlighted after a change has been made in this window.

3.4. Proxy

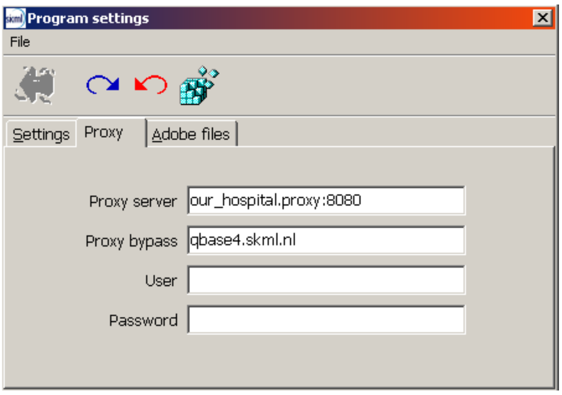

If the Internet connection is made via a proxy server, QBase can obtain the default values for this from the PC registry settings.
Since some proxy servers interfere too much with the data traffic, it may be necessary to bypass this setting. If a route to the proxy server is entered, QBase always enters **qbase4.skml.nl** in the **Proxy bypass** field.

It may be necessary to contact the system administrator in order to enter the correct information.
Clicking the button will restore the default settings.

3.5. Adobe files

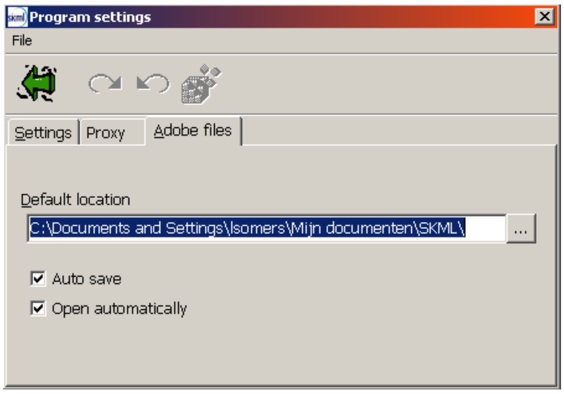

By default QBase saves pdf-files (reports and lists) in the folder "SKML".
This folder will be created as subfolder in the current users "My documents"-folder.
If necessary, QBase creates this folder.
However, users is free to create an own filing structure, as long as it is created on beforehand.
Clicking the button will restore the default settings.

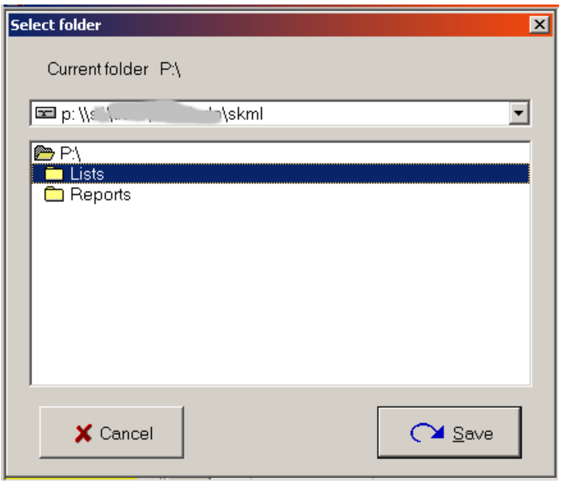

4. Downloading an agenda

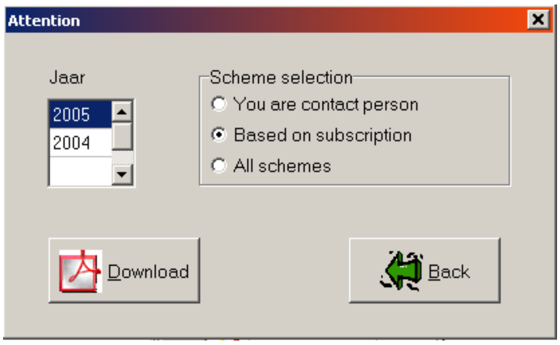

The window opposite will be displayed via the menu option: **Agenda**.
Under the heading Year, the user can select the agenda for the current year or the previous year.
At the beginning of December of each year, the following year will also be given as an option.

There are three choices under the heading **Scheme selection**.

- **You are contact person**: the user will only see the schemes in the agenda for which he or she is registered in the database as the contact person for receiving reports or samples, or for which he or she is the quality officer.
- **Based on subscription**: the user will see all the schemes in which he or she participates.
- **All schemes**: a summary of the plan for an entire year will be displayed.

Once a selection has been made, the agenda will be displayed by clicking the 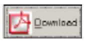 button. Clicking the 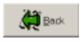 button will return QBase to the main window.

5. SKML messages

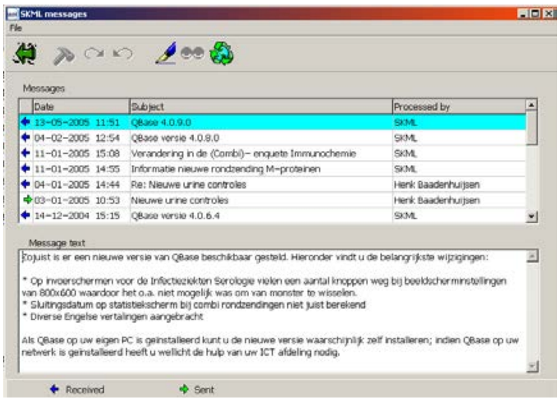

The **SKML messages** menu option allows the user to exchange messages with SKML.
This list contains all the messages sent to and received from SKML over the last year.

5.1. The tool bar

The tool bar contains a number of buttons which are context sensitive. In other words, the buttons can only be clicked if the functionality which they provide is applicable.
The buttons are:

- Close window
- Edit message [1)]
- Save changes (Ctrl+S)
- Undo
- Write new message to SKML
- Mark message as read (Ctrl+G)[2)]
- Refresh data in this window

[1)] A message can be edited as long as it is marked as **Unread** by SKML.
[2)] See "Reading a message": mark automatically as read.

5.2. The menu bar

The following functions are available in the File menu. The menu can be opened by clicking File or by using the shortcut <alt>+<B>.

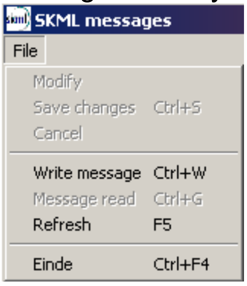

Modify		Edit the text.
Save changes	Ctrl+S	Save changes.
Cancel		Cancel.
Write message	Ctrl+W	Write a new message.
Message read	Ctrl+G	Mark an SKML message as read.
Refresh	F5	Check for new messages.
Exit	Ctlr+F4	Close window.

5.3. The context-sensitive mouse menu

The right mouse button has no functionality in this window.

5.4. The status bar

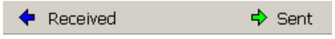

The status bar is self-explanatory.

5.5. Writing a message

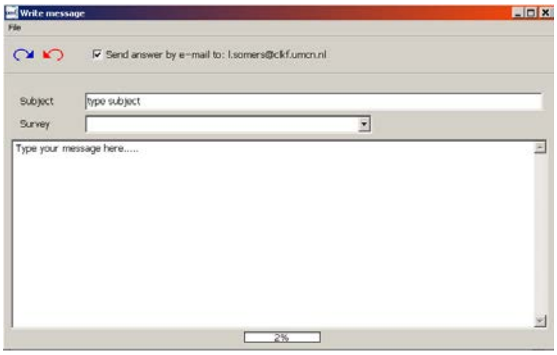

Click to open the window shown opposite.
Enter a subject and type the text of the message. The button will become active as soon as one letter of the message has been typed.
Once the message has been saved, it will be placed at the top of the message window and can then be viewed by SKML.
The **Send answer by e-mail to** check box is self-explanatory.
Whilst typing the message, the box at the bottom of the window will display how much space has already been used for the message.

5.6. Reading a message

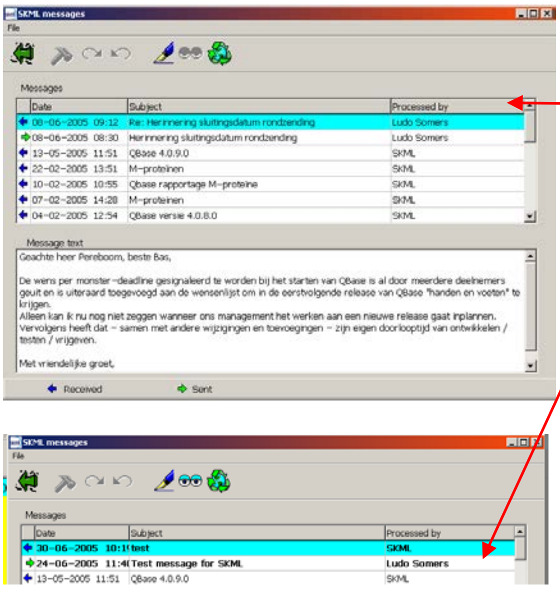

A message can be edited after it has been written, so long as it has not been processed by SKML. The status of the message in the **Processed by** column will then be **Unread**.
If an SKML employee has read the message, this can be seen because the employee's name will be displayed in the **Processed by** column.
An unread answer from SKML will be displayed in **bold**.
If a message is selected to be read, it will be automatically marked as read after 10 seconds, or earlier if the button is clicked.
Messages cannot be deleted, because they remain saved as part of the SKML quality control system.

6. Reports

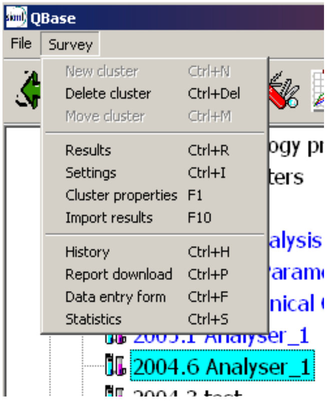

Reports of surveys can be downloaded from the main window of QBase by selecting the menu option **Report download**, by clicking the report button 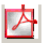 in the **Results** window or from the Statistics window.

<u>Comment:</u>
For further information, see the explanation of how to download a report from the Statistics window: 12.1.3 Reports
Detailed information is contained in the file "Graphical_interpretation.pdf".
This file is available from the downloads page on the SKML website:
http://www.skzl.nl/dl/Graphical_interpretation.pdf

7. Data entry form

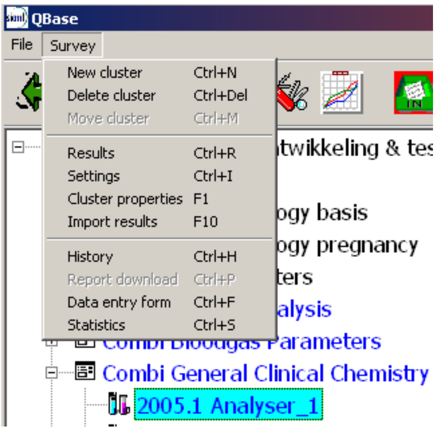

The menu option **Data entry form** will become available when a cluster is selected in the main window of QBase. For further information, see ***12.1.5 Data entry*** form.

<u>*Comment*</u>*:*
If no results have been entered in the cluster, then the data entry form will be empty.
This document can be printed via the default PDF file reader to be used as a data entry form at the workplace.

8. Clusters

A cluster must be created before data can be entered for a scheme: select the survey number, for example 2005.1, and then select the function “Create new cluster” (Ctrl+N, the button or the menu option: **New cluster**).

What happens next depends on what QBase finds in the database:

- There is no history concerning the scheme (see chapter ***8.1***).
- There is a history, but nothing can be selected because there is only one analysis environment (see chapter ***8.1.2***).
- There is a history concerning the scheme, namely a number of cluster chains (see chapter ***8.1.3***). This situation only occurs if there are a number of analysis environments, for example, two or more chemical analysers or two blood gas machines. Therefore, more than one chain can be continued with.

Comment:
A new cluster can only be created in a time window of 14 days before the start date of a survey until 28 days after the deadline. However, after the deadline, no more results may be entered. It will still be possible to create a cluster, though, and a report can be generated based on this cluster.

8.1. Creating a (new) cluster

8.1.1. There is no history

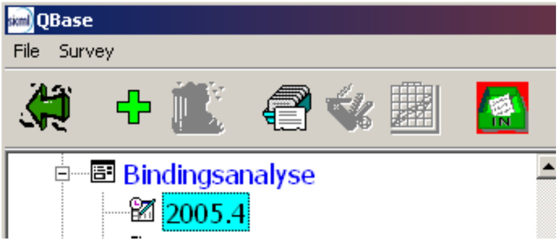

Select the scheme and then the survey in the scheme for which a new cluster must be created and then click the button.

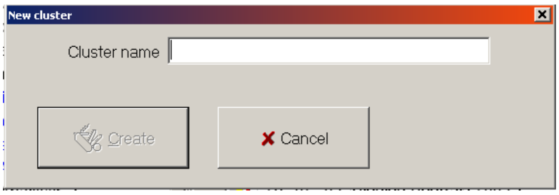

In the **Cluster name** field, type a name for the analysis environment which can help to distinguish the results from a second set of results. The Create button will become active when typing a name. Click this button to create the cluster. QBase will then open the **Cluster settings** window.

Comment:
The name which must be entered only becomes significant once a number of data sets or clusters have been created. QBase will suppress the name if only one cluster chain has been created. See the examples of possible windows in the next chapters.

8.1.2. There is a history, but nothing can be selected

If, when creating a new cluster, it is only possible to continue based on one cluster which is present from previous surveys, QBase will create a new cluster based on the

settings of the previous cluster. Depending on the type of scheme, QBase will open the **Analyse data** window for entering the measurement moments or the **Results** window (for Combi schemes).

8.1.3. There is a history from which to make a selection

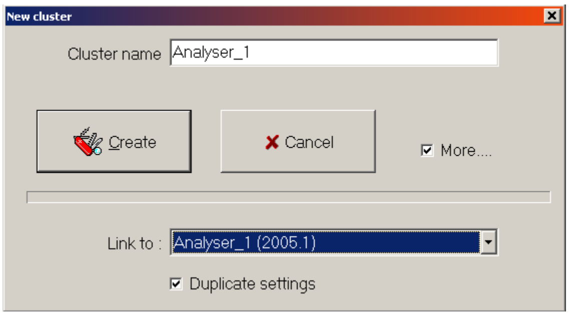

If there are a number of cluster chains in the history – a number of measurement environments which are being checked - QBase will offer the option to select which cluster <u>chain</u> the new cluster must be linked to. To do this, select the **More…** check box.
Selecting the **Duplicate settings** check box indicates that the settings for the old cluster must also be used for the new cluster.

QBase will create a new cluster in the selected chain and then open the **Sample dates** window.

8.1.4. Entering the sample dates

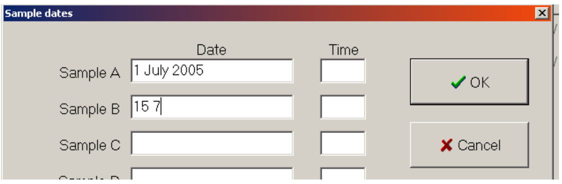

One of the properties of a result is “when the measurement was taken”. This must be entered via the **Sample dates** window opposite.
The date will be displayed in the format 2 January 2005. Any other entries, for example 21 1 5 or 21-01-05 will automatically be changed to the valid format.
Typing T (Today) will give the current date. The day can be moved backwards or forwards using the + and – keys.

There is an option to enter an analysis time in the right-hand field (*<u>hh:mm</u>*).
By default, QBase will display the number of samples which have been defined within the context of the scheme concerned. There are two samples in this example, **Sample A** and **Sample B**.
There are 6 samples (samples A to F) for the example scheme.
The **Results** window will then be displayed and the results can then be entered in this window.
<u>Comments</u>

- *If a date is entered which falls outside the time frame between start date and deadline, the background will become yellow and the OK button will be deactivated.*
- *For internal control material (for the COMBI schemes), the analysis dates are only entered in the result window or created when importing the results (also see chapters 10.7.4 and 10.8).*

8.1.5. When a new cluster has been created

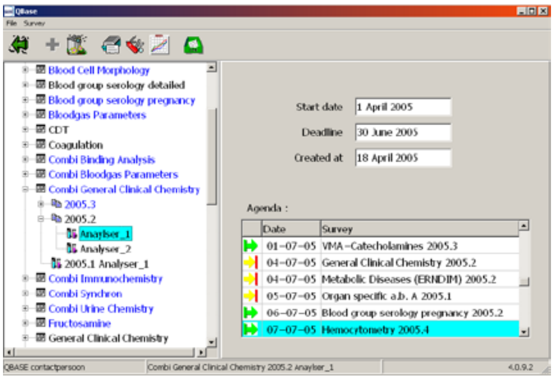

An icon is placed before the survey number to indicate that a (new) cluster has been created for that survey.

indicates that one cluster has been created in that survey to process the data.

indicates that a number of clusters/cluster chains have been created for that survey.

8.2. Deleting a cluster

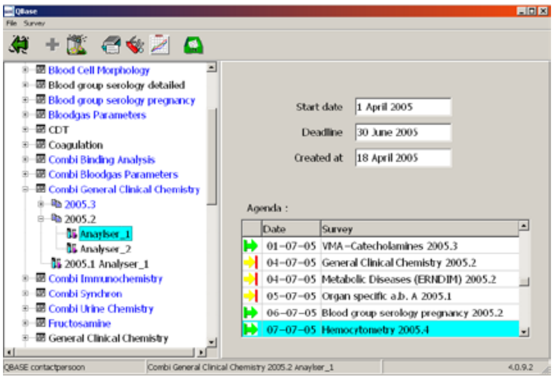

A cluster can also be deleted, but only under certain conditions.
A cluster can be deleted if:

- There are no results present.
- The deadline has not yet passed.

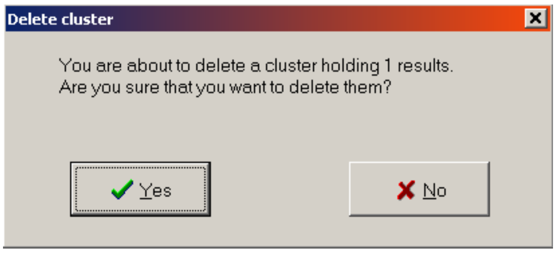

A warning will be given if the cluster to be deleted contains any results.

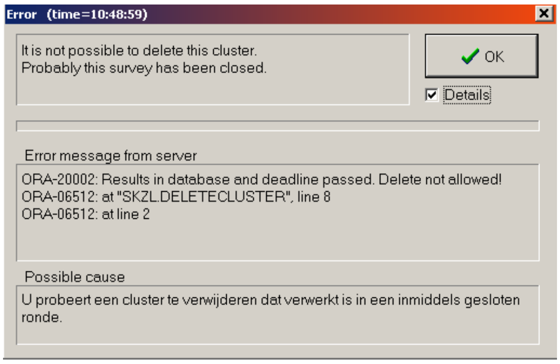

It may not always be possible to delete a cluster.

More details will be displayed if the **Details** check box is selected.

8.3. Changing the name of a cluster

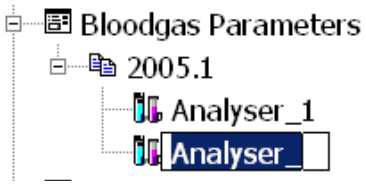

Select the cluster and press the <F2> key. It will then be possible to change the cluster name.
Press the <Enter> key after changing the name.

8.4. The history of a cluster

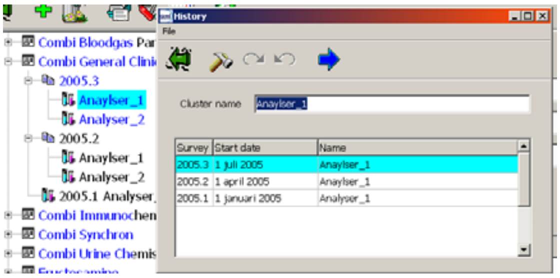

The **History** function displays the history of a certain cluster. Select a cluster and press Ctrl+H.
This function can be used, for example, to find out in which chain each of the two clusters from the previous paragraph belong so that they can be included in the same report.

8.5. Moving a cluster

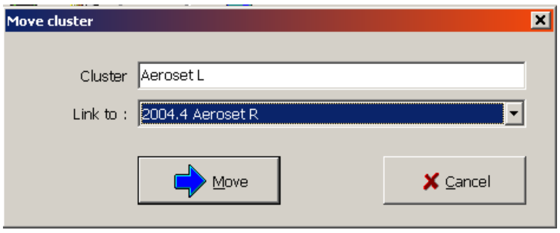

It is possible to move a cluster from one chain to another. If a new cluster is accidentally created in the wrong chain, it can be moved to the correct chain.

- Select the cluster to be moved.
- Click **Move cluster**.
- In the **Link to** field, select the chain to which the cluster must be moved to.
- Click 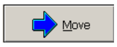.

9. Cluster settings

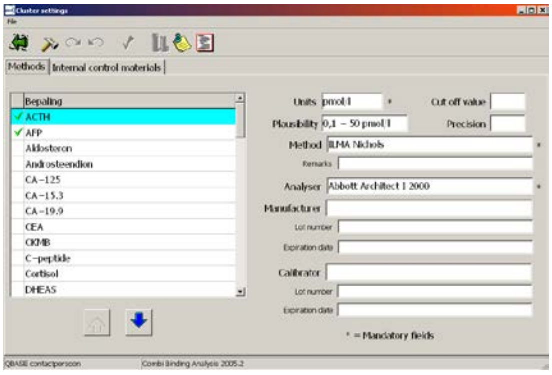

If, when creating a cluster, it appears that there are no cluster settings, QBase will automatically open the **Cluster settings** window.
This window can be used to indicate (select) for which analytes you wish to enter results and in which order.
The following must be indicated for each analyte:

- The **unit***).
- The analysis **method** used.
- The **analyser** used.

*) <u>*Comment*</u>:

- *The unit can only be given for analytes for which SKML supports the use of different units. The default unit is then given, but can be changed to one of the units which can be selected via the selection box.*
- *If an ❗ is placed before an analyte, this means that an analyser or method setting which is no longer valid has been entered for the analyte. This must, therefore, be altered.*

9.1. The tool bar

The tool bar contains a number of buttons which are context sensitive. In other words, the buttons can only be clicked if the functionality which they provide is applicable. The buttons are:

Close window (Ctrl+F4)	Select analyte
Edit	Go to **Add/remove internal control material**
Save changes (Ctrl+S)	Go to **Remarks**
Undo (Alt+BkSp)	Display cluster properties (F1)

The menu bar

The functions shown below are available in the File menu. The menu can be opened by clicking File or by using the shortcut <alt>+<B>.

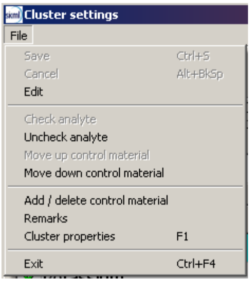

Save changes	Ctrl+S	Save changes
Cancel		Cancel
Edit		Edit settings
Check analyte		Select analyte
Uncheck analyte		Unselect analyte
Move up control material		Change order
Move down control material		Change order
Add/delete control material		Add/delete control material
Remarks		Go to **Remarks**
Cluster properties	F1	Go to **SKML report** and display the cluster properties
Exit	Ctrl+F4	Close window

9.3. The context-sensitive mouse menu

The mouse menu contains the following functions:

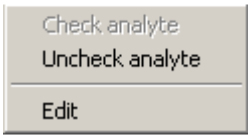

Display the analyte in the result window.
Hide the analyte in the result window.
Edit the method settings, etc.

9.4. The status bar

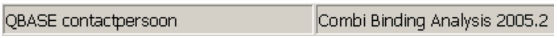

The status bar displays the name of the user who is logged in, together with the name of the scheme and the survey.

Creating cluster settings

The layout of the window and the information which is displayed depends on the type of survey for which the settings must be entered or altered.
As stated in chapter ***0 Introduction***
only External and Combi schemes are described in this instruction manual (see chapter ***9.6***).

- **External**
  The analyser and method (and sometimes the unit of the result) are given for each analyte.
- **Combi**
  The same as for External, but also includes information about the internal control materials used.

Comment:
If, when creating a cluster, the deadline of the survey has elapsed, it is still possible to create a cluster in QBase, but it will not be possible to make any alterations to it.
*By creating a cluster, it will be possible to download a report of the survey concerned (see **6** Reports).*

9.5.1. Altering cluster settings; automatic settings

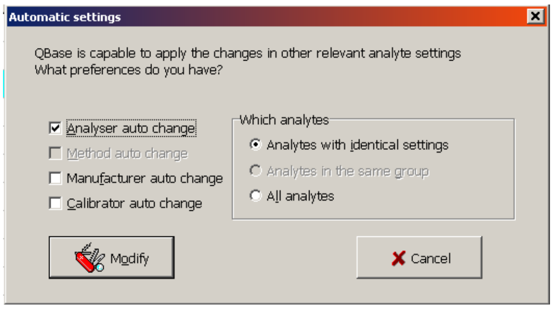

If QBase notices that an altered setting can also be used for another analyte, the window shown opposite will be displayed to ask whether changes must be applied to all the analytes at once.
The choices which can be made in this window are self-explanatory.

9.6. Cluster settings

The term cluster settings applies to all the analyte settings which have been selected (ticked) to be included in a certain survey of a scheme.
This concerns settings such as the analysis method used, the analyser used, if any, the unit of the result, details concerning the reagents and calibrators used.

9.6.1. Methods (External and Combi)

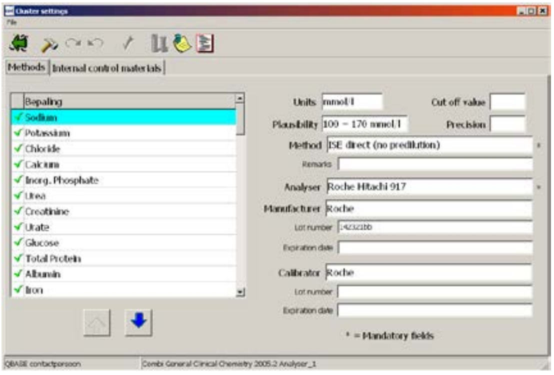

Analyte settings which are included can be entered or edited via the **Methods** tab.
In order to add new settings or alter existing settings, the analyte must first be selected using the mouse before clicking the edit button.
Selection boxes will then become visible in the right-hand panel. They can be expanded by clicking the icon.

Select the correct method, analyser and unit (also see the comments).
Mandatory settings are:

- **Unit**
- **Method**
- **Analyser**

If a method and analyser have been selected for an analyte, they can be saved by clicking the button; this will also automatically activate the analyte. The icon will then be displayed before the analyte.
If the button is clicked, nothing will be saved and the choices made will be cancelled.
The analyte settings which had results saved before the deadline will be locked after the deadline has elapsed. This is made visible by the icon.

Comments:

- *A unit can only be selected if the analyte supports the use of a number of different units.*
- *If an ! is positioned before an analyte, this means that an analyser or method setting which is no longer valid has been selected for the analyte. This must, therefore, be altered.*
- *The **Cut off value** field is (currently) not used.*

- *The number of decimal places of the calculated averages can be set in the* ***Precision*** *field.*
- *The limit values set by SKML are given in the* ***Plausibility*** *field.*
- *This field will only be displayed if there is a value to display.*
- *Each result which is entered will be compared to the values in this field. If the result which is entered falls outside of the indicated limits, a message will be displayed which asks for confirmation of the deviating result.*
- *The other fields -* ***Manufacturer****,* ***Lot number****,* ***Expiration date, Calibrator****,* ***Lot number*** *and* ***Expiration date*** *– are optional. They will be saved in the database and displayed each time, but nothing is (currently) done with these values in the (central) software. Furthermore, these fields will only de displayed if information concerning the manufacturer of the reagent used can be selected. In other cases, these selection fields will not be active.*

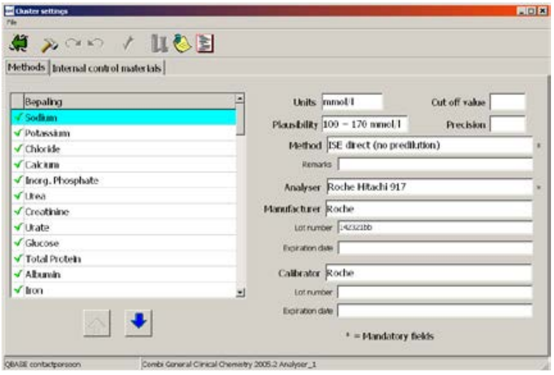

As soon as settings have been entered for an analyte, the analyte will be automatically activated when the settings are saved using the button.
If an analyte is no longer included, it can be deactivated by double-clicking the button which is placed before the analyte or via the menu option: **Uncheck analyte**.

Comment:

The analytes are initially sorted in the "SKML order".

- *The order within this group of selected analytes can be altered using the and buttons. Select the analyte to be moved and then click one of the buttons. After every click, the analyte moves one position up or down in the list (as far as the first or last position). The change can then be saved by clicking the button.*
- *An icon before the analyte means that the analyte is not externally supported by SKML. The analyte can then only be used in the context of internal controls and cannot, therefore, be selected in the Methods tab. It must be selected in the Internal control materials tab.*

If the order of the analytes becomes confusing, it can be returned to the default order via the menu option: ***Default order****.*

9.6.2. Internal control materials (only Combi schemes)

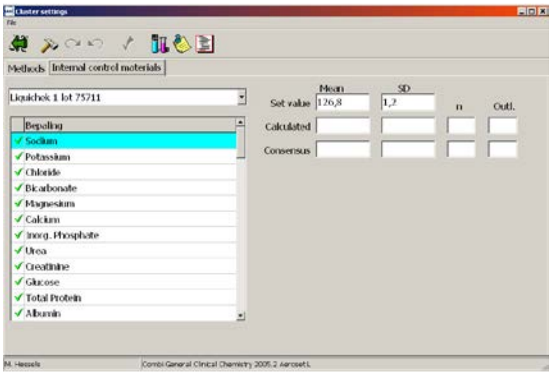

The **Internal control materials** tab is only available for Combi schemes. If the settings for the internal control materials have not yet been created, the window is as shown opposite. In order to create a number of settings, an internal control material must first be entered. To do so, click the button.
The following window will then be displayed in QBase.

The **Set value**, **Calculated** and **Consensus** fields are explained in chapter ***9.6.4 Adjusting user limited values***.

9.6.2.1. Selecting an internal control

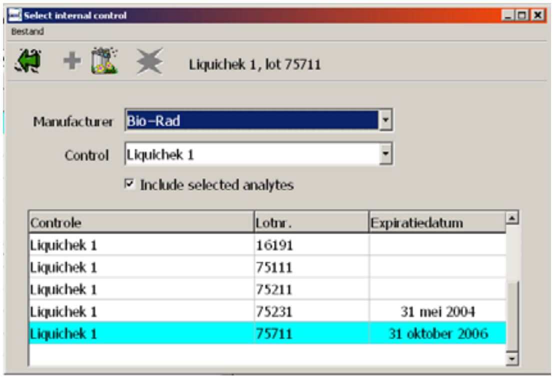

The **Select internal control** window contains a detailed list of <u>all</u> the control materials which are *currently* included in the SKML tables.

9.6.2.2. The tool bar

	Close window	(Ctrl+F4)
	Add control material	(Ctrl+T)
	Delete control material	(Ctrl+Del)
	Exchange control material	(Ctrl+W)

9.6.2.3. The menu bar

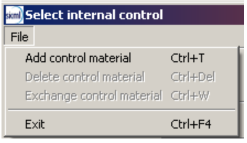

The functions shown opposite are available in the File menu. The menu can be opened by clicking File or by using the shortcut <alt>+<B>.

Add control material	Ctrl+T
Delete control material	Ctrl+Del
Exchange control material	Ctrl+W
Exit	Ctrl+F4

9.6.2.4. Selecting an internal control

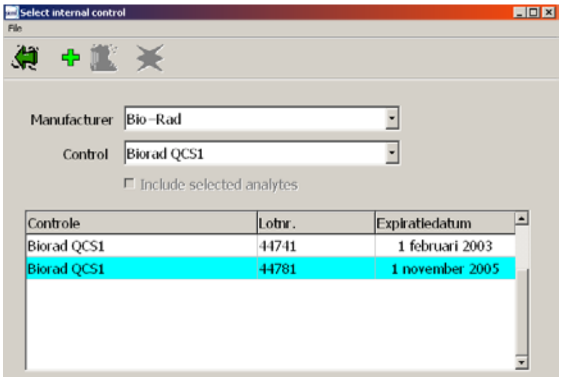

Selecting a **Manufacturer** of a control material limits the materials included in the list considerably.
Next, select the **Control** based on the correct name and the lot number and then click the button.
If this is the first internal control which is added, QBase will open the previous window so that the analyte which will be used for the control which has just been added can be selected .

<u>Comment</u>:
If the control used is not included in the list, or does not have the correct (latest) lot number, then the control is not (yet) known by SKML. You can request the CFS (Central Facilities Service) (via an e-mail to office@skml.nl or via a QBase message) to include the new internal control. The new control can be selected once it has been included in QBase.

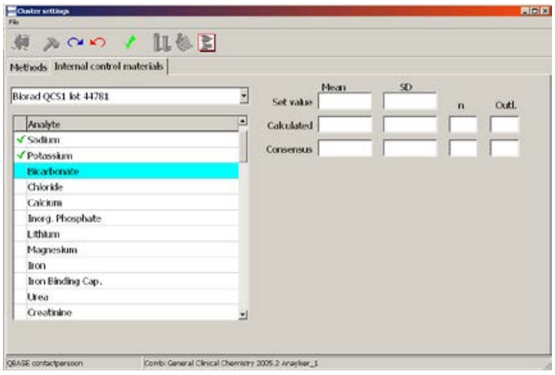

Once the internal control(s) has (have) been added, the analytes must still be selected.
<u>This is not done automatically as is the case for the analytes of external samples.</u>
Select the analyte and click the button.
Save the selection by clicking the button.

Tip:
Select an analyte by double-clicking the field before it.

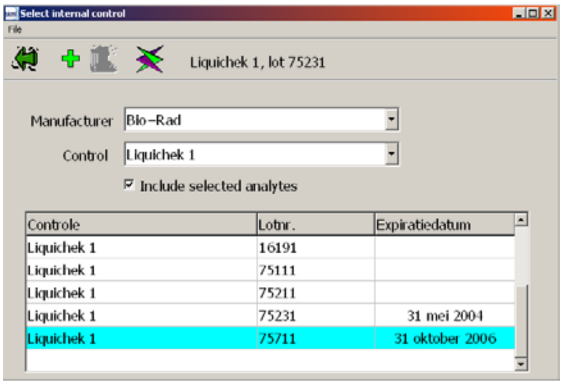

If one or more internal controls are already present, QBase will ask whether the settings of the old control must be used for the new control. Select **Include selected analytes** and then use the tool bar to select the control whose settings will be copied. **Include selected analytes** is selected by default, because this will usually be the intention.
Select the next control and add this by clicking the button.

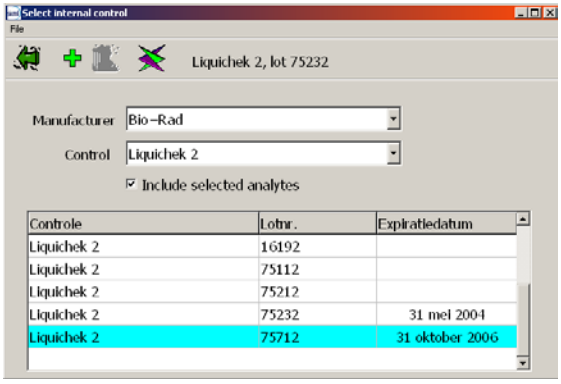

Select other controls and add these by clicking the button.

Comment:
QBase supports adding an unlimited number of internal controls per analyte.
*In fact, an endless list of controls can be added. However, since the information on the report will then be represented in such a way that it will no longer be easy to read, it is recommended to <u>not link more than 4 internal controls</u> to an analyte. Be aware of this and use the **Include selected analytes** function sensibly.*

9.6.2.5. Exchanging internal controls

At some time, the existing control material will be replaced by a new control material, often from the same manufacturer and with the same name, but with a new lot number and a new expiration date.
The **Exchange control material** function is very useful when this happens.
Carry out the following:

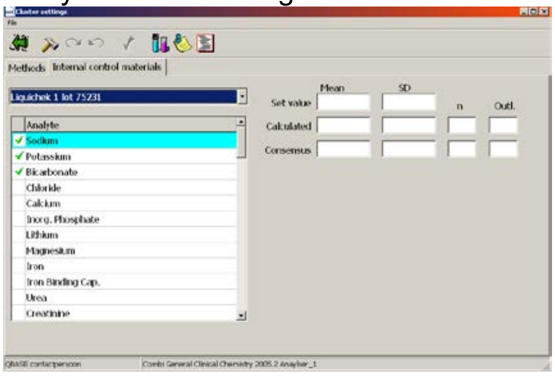

Select the internal control to be replaced in the **Cluster settings** window and click the button.

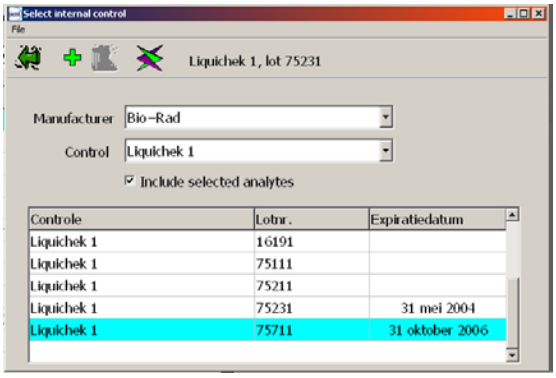

Select the new internal control and *__the new lot number__* for the desired manufacturer and click the exchange button .

__Comment:__
If the control used is not included in the list, or if it does not have the latest lot number, then the control is not (yet) known by SKML. You can request the CFS (Central Facilities Service) (via an e-mail to office@skml.nl or via a QBase message) to include the new internal control in the list. It will be possible to select the new control can be selected once it has been included in the list.

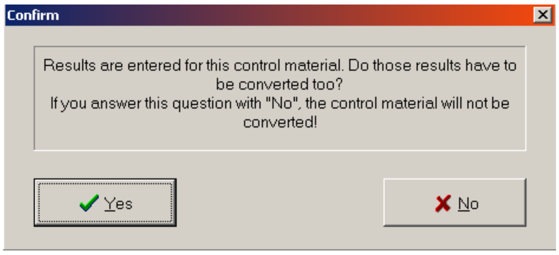

If, when __exchanging__ control material, results are already present in the database, QBase will report this and ask for confirmation for the exchange. QBase will include the results if the Yes button is clicked.
__Comment:__
This is possible for as long as the survey has not been closed.

9.6.3. Order of internal control materials

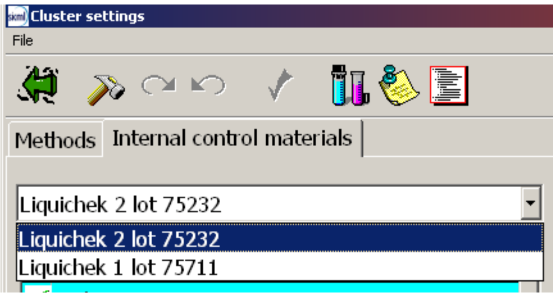

The order of the internal control material may not be ideal.
QBase includes a function which can be used to change the order.

9.6.3.1. Changing the order of the internal control materials

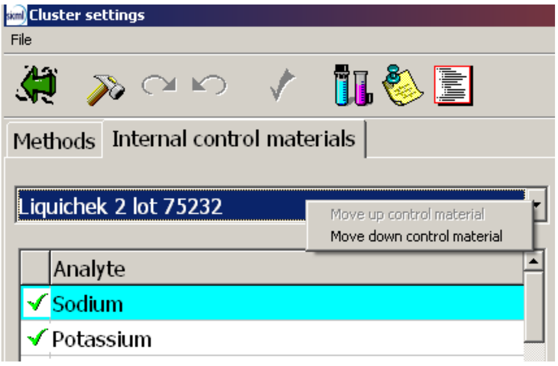

Select the control which must be moved up or down from the list and **right**-click the field or open the File menu.
A sub-menu will then be displayed with the following functions: **Move up control material up** and **Move down control material down**.
Click the desired direction to change the order.

9.6.4. Adjusting user limited values

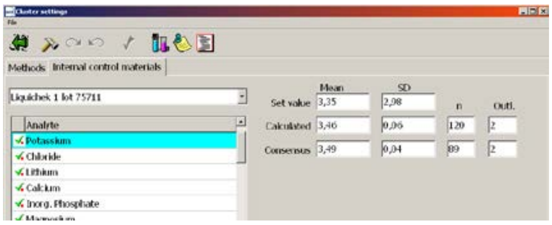

It is possible to set limit values for every combination of internal control/analyte: enter the mean value and the accompanying SD in the **Set value** field. Every result which is entered will be compared to the set limit values **<u>and</u>** the result will be checked to see whether it falls within the plausibility limits set by SKML. For any deviations, QBase will request confirmation that the result has been entered correctly or whether it is necessary to enter the correct result.

The results of the statistical calculations of the selected analyte of the internal control are displayed in the **Calculated** fields. These values are re-calculated every night after alterations or additions.
The same is also displayed in the **Consensus** fields, but then for the results of the selected analyte with this internal control of all the participants.

9.7. Summary of the cluster properties

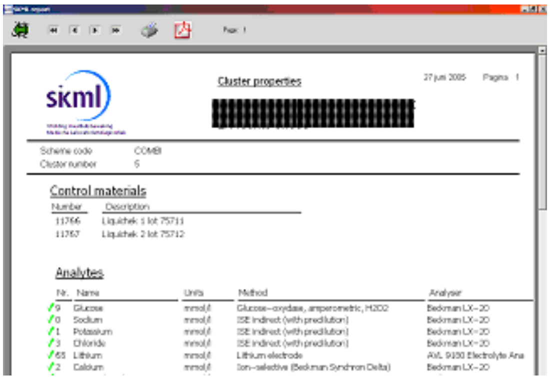

The main reason why this report is included is for the benefit of importing results. The summary shown opposite is displayed by clicking the button (Cluster properties summary) or by pressing the <F1> key.
The heading contains information about the scheme and survey, followed by the cluster name.
The **scheme code** and the **cluster number** are given between the thick lines.
If defined in the cluster concerned, information about the internal control materials in use will be given below the line.
The list contains all the analytes, together with the analyser and the method used for each analyte.

Comment:
The numbers which are given before the internal control materials and the analytes are unique SKML code numbers for the components concerned.
The numbers must be included in the import file together with the code name of the scheme and the cluster name when importing results from laboratory systems.
See section ***10.8*** in the “Results" chapter.

9.8. Remarks

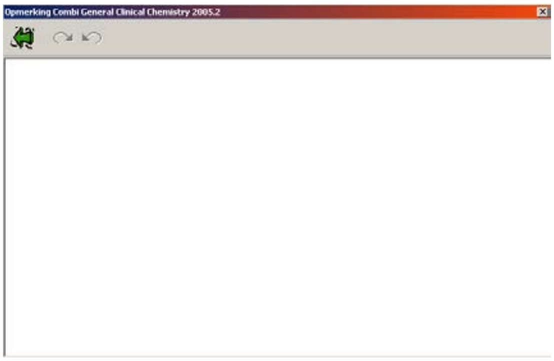

The window opposite is displayed by clicking the Remarks button .
This window can be used by the user to enter relevant comments which will be saved at the cluster level.

This window can also be opened from the **Results** window.

Comment:
The data entered here is only for internal use by the participant.

10. Results entry

The Results window can be opened in a number of different ways from the main window of QBase after a survey has been selected:

- Click .
- Double-click a survey.
- The shortcut <Ctrl><+R>.

An explanation of the general functionality will be given first. Later, a distinction will be made (just as for chapter 4 “Cluster settings”) between:

- **External**
  The external form of entering results. There are several variants which depend on the type of scheme (also see the brief, specific instructions which relate to the scheme).
- **Combi**
  A way of entering results where it is also possible to import results from a lab system.

10.1. The tool bar

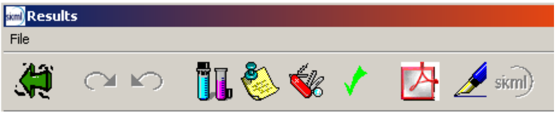

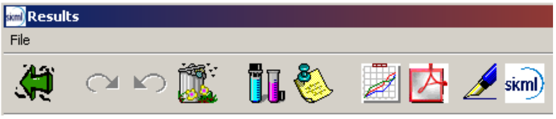

The tool bar contains a number of context-sensitive buttons. In other words, the buttons can only be clicked if the functionality which they provide is applicable. Not all of the buttons are displayed in every result entry window. Only those which (can) have a significance will be displayed.

The buttons are:

Close window

Save changes (Ctrl+S)

Cancel (Alt+BkSp)

Delete sample (Ctrl+Del)

Go to **Sample dates** (Ctrl+A)

Go to **Remarks**

Go to **Settings** (Ctrl+I)[1)]

Validate

Go to **Statistics**

Download report (Ctrl+R)

Send e-mail (Ctrl+E)

Send QBase message (Ctrl+Q)

[1)] Not for Combi schemes

10.2. The menu bar

The functions shown below are available in the File menu. The menu can be opened by clicking File or by using the shortcut <alt>+<B>.

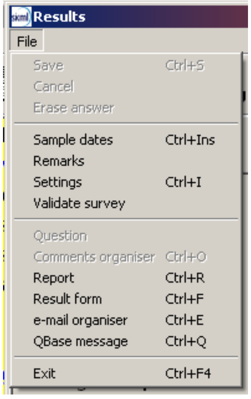

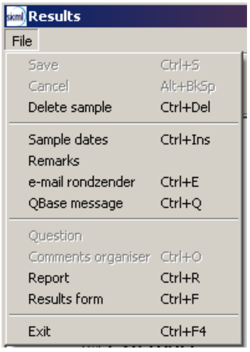

Save	Ctrl+S	Save changes.
Cancel	Alt+BkSp	Cancel
Erase answer		Delete result[1)]
Delete sample	Ctrl+Del	Delete sample[1)]
Sample dates	Ctrl+Ins	Go to **Sample dates**[1)]
Remarks		Go to **Remarks**
Settings	Ctrl+I	Go to **Cluster settings**[1)]
Validate survey		Validate survey (**survey will be locked!**)
Question	Ctrl+Q	Question concerning the test to be carried out[1)]
Comments organiser	Ctrl+O	Added comments[1)]
Report	Ctrl+R	Download report
Result form	Ctrl+F	Print result form
e-mail organiser	Ctrl+E	Send e-mail to organiser
QBase message	Ctrl+Q	Go to **SKML Messages**
Exit	Ctrl+F4	Close window

[1)] Not for all schemes

10.3. The context-sensitive mouse menu

The mouse menu contains the following functions:

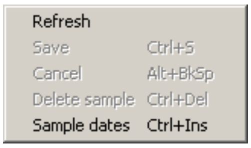

Refresh		Refresh data in the window
Save	Ctrl+S	Save changes
Cancel	Alt+BkSp	Cancel
Erase sample	Ctrl+Del	Delete sample
Sample dates	Ctrl+Ins	Go to Sample dates

10.4. The status bar

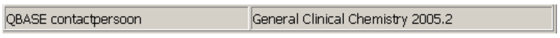

The status bar displays the name of the contact person and the name of the scheme with the selected survey.

10.5. Entering results

Before the various windows for entering results are explained, a number of general comments will be given which concern all the windows used for entering results:

- It is only possible to enter results if the sample date has been entered for the sample to which the analyte belongs (see chapters ***8.1.4 Entering the sample dates*** and ***10.7.4 Entering analysis dates for INTERNAL control material***).
- The results which are entered will be checked in all the windows where this is possible.

- In every window, it is possible to add a comment to a cluster in order to record special or unforeseen circumstances.

The windows for this function will now be explained.

10.5.1. Plausibility check of the entered results

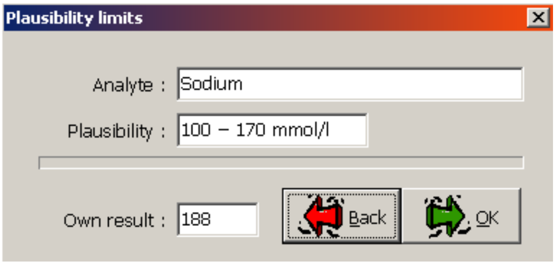

If the result which has been entered (in this example, **188** for **Sodium**) falls outside of the plausibility limits, a window will be displayed which requests confirmation of the result.
Clicking the Back button will return to the result entry field so that the result can be corrected. The result is accepted by clicking the OK button.

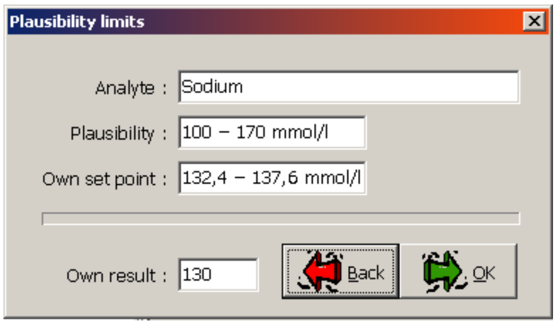

If the result which has been entered (in this example, **130** for **Sodium**) falls outside of the plausibility limits, a window will be displayed which requests confirmation of the result.
Clicking the Back button will return to the result entry field so that the result can be corrected. The result is accepted by clicking the OK button.
When entering results for your own *Internal* control materials, the result will also be tested against your own limit values. For an explanation, see ***9.6.4 Adjusting user limited values***.

10.5.2. Validating the results

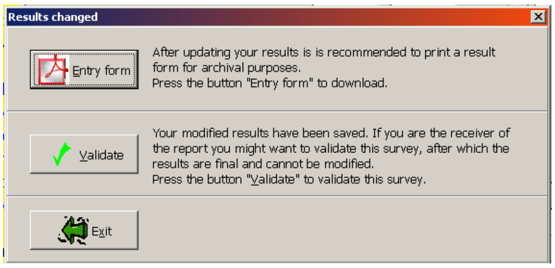

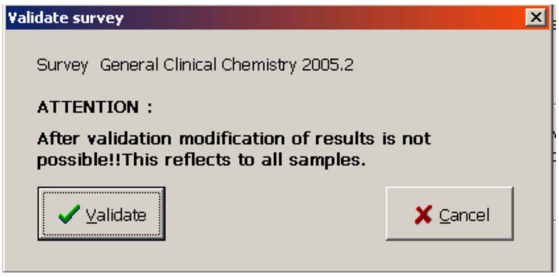

When the result entry window is closed after saving the results, QBase will display the window shown opposite to ask what the next step must be:

- **Entry form** displays a data entry form which contains the results which have been entered; suitable for archiving.
- **Validate**: this functions is <u>only</u> intended to make it impossible to add or edit results within your own organization. In view of the implications of this function, further confirmation will be requested.
  NOTE:
  It is not possible to undo this!

<u>Comment:</u>
The Validate function is not available for schemes which have deadlines for each separate sample. (e.g. all schemes of the Combi type). The button will then be grey.

10.5.3. Remarks

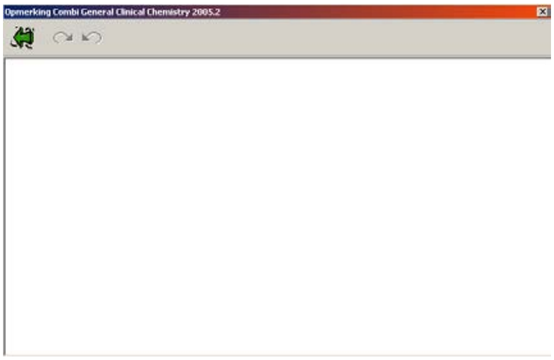

The window opposite will be displayed by clicking the 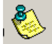 button.
Up to 1,000 characters of text can be typed in this window.
These remarks will be saved at the <u>cluster level</u>.
This window can also be opened from the **Cluster settings** window.

10.6. Result entry window, type External

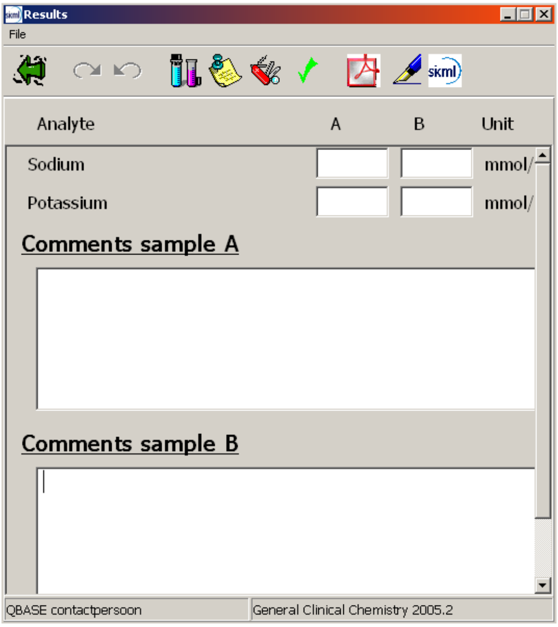

The most simple form of the result entry is as shown opposite in the example for General Clinical Chemistry.
The save and cancel buttons will become active as soon as a result is entered.

Comments:

- *Only analytes for which settings have been entered and which have also been selected will be displayed in the same order as that which has been indicated by the participant in the Settings window.*
- *The save and cancel buttons will become active as soon as something is typed in the result field.*
- *Comments which are given for the various samples **will not be included in the data entry form nor the reports**.*

10.7. Result entry window, type Combi

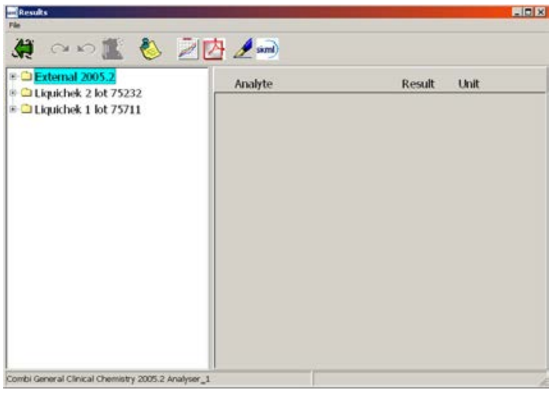

This is the first window that will be displayed by QBase in the example of the Combi General Clinical Chemistry scheme.
Click on a node + sign to expand the tree.
If the + sign in front of **Samples** is clicked, QBase will open the **Result entry per sample** window. Clicking **Analyte** will open the **Result entry per analyte** window.

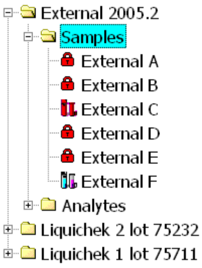

10.7.1. Result entry per sample

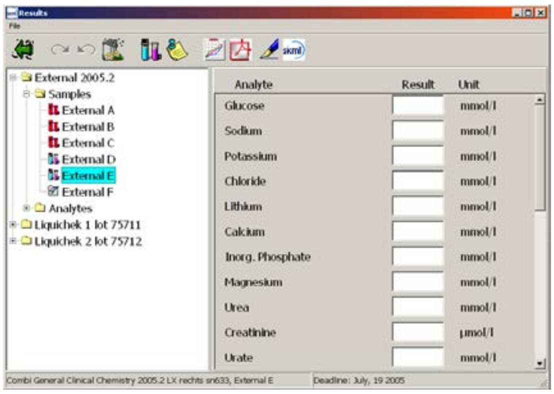

For the (external or internal) control material to be processed, expand the tree by clicking the +sign and then click **Samples** to see the result entry <u>per sample</u>.

<u>Comment:</u>
The analytes shown are those which have been selected in the settings. They are displayed in the same order as in the settings.

Different icons may be displayed before a sample.

- For this sample, the deadline has expired without a sample date being entered. Therefore, no results have been entered.
  This sample is locked.
- For this sample, the deadline has also expired, but a sample date has been entered in time, followed by the results.
  This sample is locked.
- For this sample, the sample date and results have been entered. Results can still be added or edited.
- For this sample, the sample date has been entered, but no results have yet been entered.
- This sample is not yet active.

10.7.2. Result entry per analyte

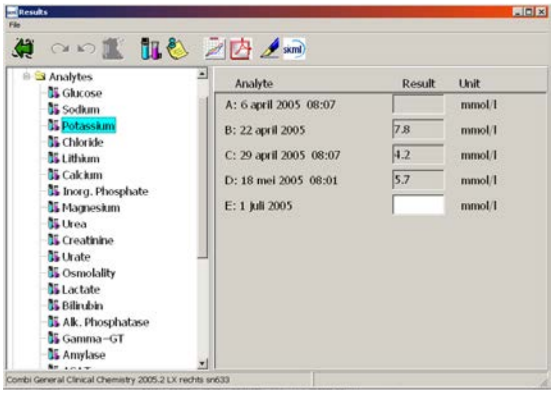

For the (external or internal) control material to be processed, expand the tree by clicking the +sign and then click **Analytes** to see the result entry <u>per analyte</u>.

<u>Comment:</u>
The analytes shown are those which have been selected in the settings. They are displayed in the same order as in the settings.
The samples are sorted according to date/time.

10.7.3. Why is the result field deactivated and entry not possible?

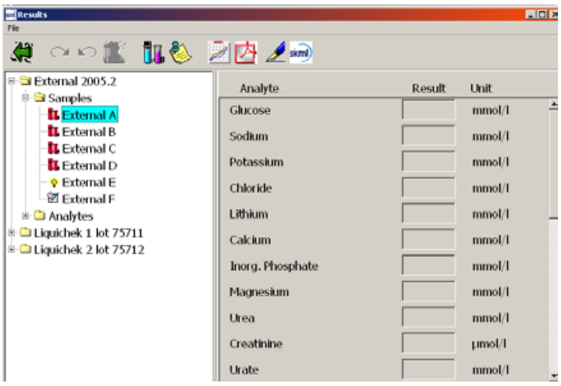

A common problem is that the fields for the result entry are deactivated.
This can be caused by one of three different reasons:

- Too late; see ***10.7.1***
- The deadline has expired; see ***10.7.1***
- There is no sample date; see ***10.7.1***

10.7.4. Entering analysis dates for INTERNAL control material

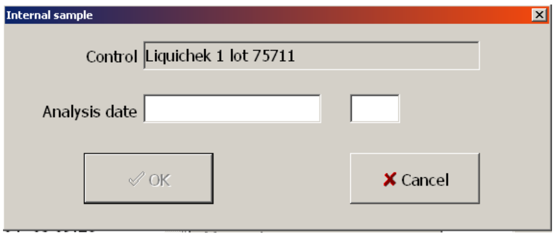

In order to enter results for **internal** samples, the analysis dates must first be entered before the results can be entered.

Expand the tree for an internal control material and click the Samples button. The button will then be added to the tool bar. Clicking this button will open the window shown above. This window can then be used to create samples.

Comment:

*Normally, all the results of the COMBI schemes will be imported. The samples with their respective sample dates will then be created when imported (also see chapter **10.8**).*

10.8. Importing results

Comment:

*Although described here, this function is only available from the main window of QBase and not via the **Results** window. This is due to all the technical implications of regularly refreshing the content of windows.*

For a number of schemes, a large number of results must be processed per survey. This must all be entered via QBase. In order to make this task easier, a function has been included to import all these data via one processing action. This functionality even extends to extra functions in commercial laboratory systems, where the results which must be sent to QBase are prepared via a separate function in a text file with a special, fixed SKML composition (see chapter ***13***).

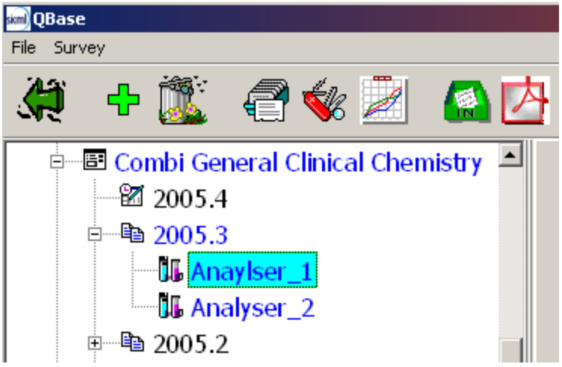

Before results can be imported, the cluster in which the results must be saved must first be selected.

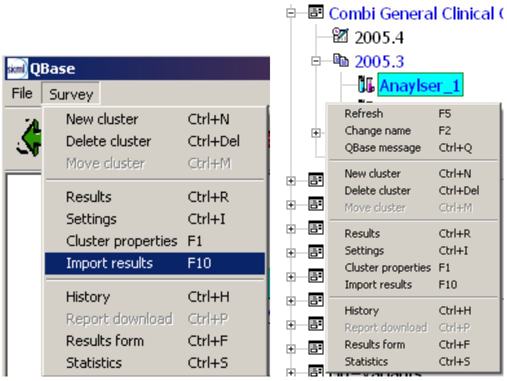

Next, click **Import results** in the **Survey**-or mouse-menu or press the <F10> key.

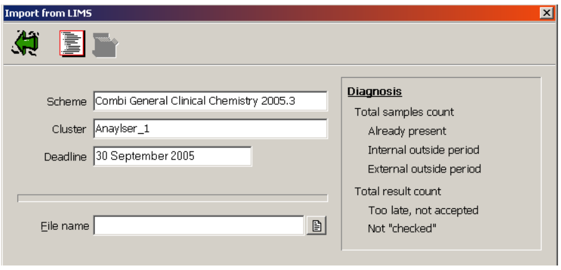

The window shown opposite will then be opened.
The window indicates where the results to be imported will be added and before which date this must take place.
Specify the location and name of the import file in the **File name** field or…

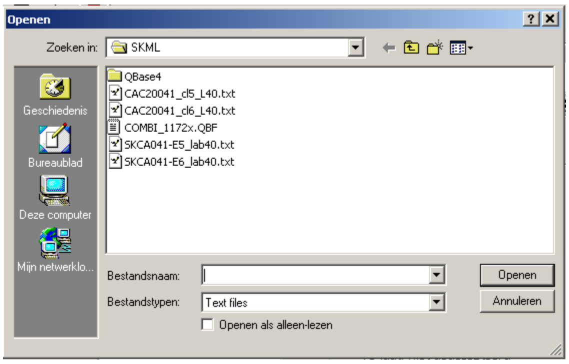

click on the 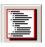 button or the 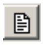 button to search for a file. Double-clicking the file name or selecting the file and then clicking Open will open the file in QBase.

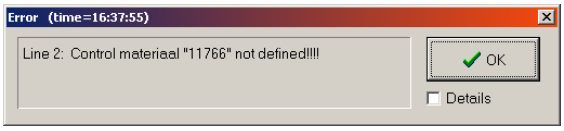

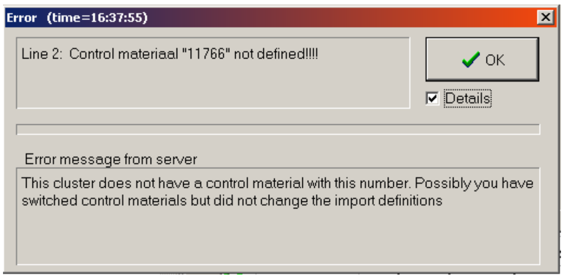

QBase will first check the file for errors which will block the import.
Any errors will be displayed via an **Error** window.

10.8.1. Import function diagnosis

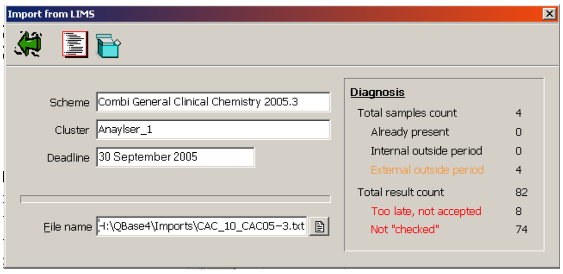

Under the heading **Diagnosis**, QBase will display the number of samples and the number of results in the import file and some further information relating to the count.
Not "checked" means that an analyte (number) which is present in the import file has not been selected in the Cluster settings window.

If QBase does not find any errors or problems, click the 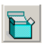 button to start the import.

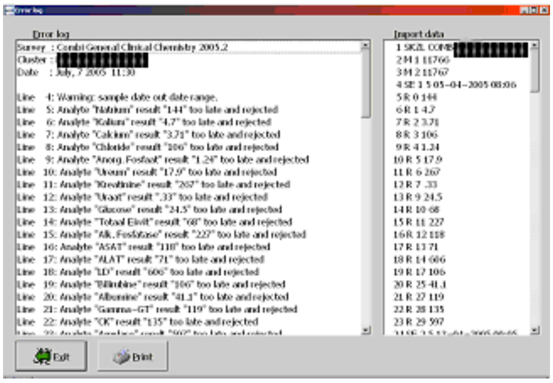

It will be explained exactly what the error is for each line in the import file (right-hand column). **Therefore, there is a left-right link**. The comments in the error log are self-explanatory, but if you have any questions, please send an e-mail to SKML via office@skml.nl, stating what the question is and attach the import file concerned.

<u>Comments</u>:

- QBase cannot determine what the error is before the import takes place. For technical reasons, it is only possible to do so when the line concerned is processed.
- The summary can be printed by clicking the print button.

10.9. Downloading a report

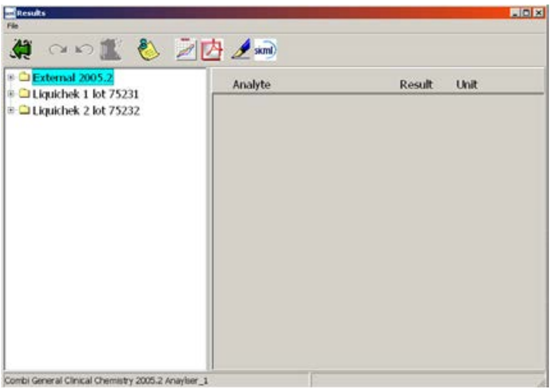

A SKML report of the cluster concerned can be downloaded by clicking the report button in the **Results** window.

11. Report management

11.1. Availablity of reports

Reports and lists are available in a number of ways.

- As automatic service by e-mail after closure of a survey.
- Via QBase users can dowload the just filed report as well as recreate an old report from the past. It is also possible to download different type of reports.
- The website delivers equal functionality as QBase, however only some standard reports.

Reports are filed on the reportserver of the SKML after closure of a survey and after final approval of the representative of the responsable section of the SKML.
Reports are available for a period of 90 days. Subsequently, reports are recreated on user request.

11.2. Getting reports

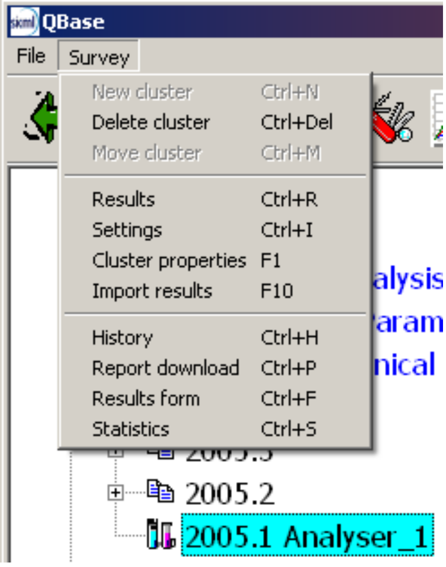

Reports are available in three different ways:

- on the main window via the menu option **Report download** or by clicking the button, or by using the key F12.
- on the **Results** window by clicking the button.
- by making ther appropriate selections on the **Statistics** window.

11.3. The report management window

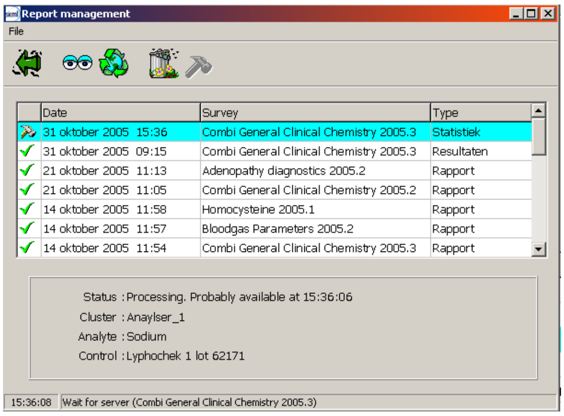

The window **Report management** will occur automatically if it takes longer then a few seconds to present the report.

In this window all reports and lists are showed which have been requested by the participant, including the reports created automatically after closing of a survey.

Remarks:

- *Underneath the table **Status** shows how long it takes to create the report or list. When ready this field shows how long (jn days) the file will be stored on the reportserver..*
- *The fields* **Cluster**, **Analyte** *and* **Control** *are added when applicable.*

11.3.1. The tool bar

The tool bar contains a number of buttons which are context sensitive. In other words, the buttons can only be clicked if the functionality which they provide is applicable. The buttons are:

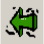 Exit

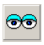 open file (Ctrl+O)

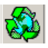 refresh (F5)

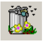 delete report (Ctrl+Del)

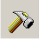 Rebuild report

11.3.2. The menu bar

The following functions are available under the menu options **File**. The menus can be opened by clicking on them and then selecting the desired option or using the shortcuts.

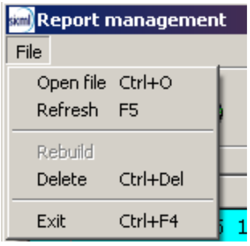

Open file	Ctrl+O	open file
Refresh	F5	refresh table
rebuild		
Delete	Ctrl+Del	
Exitl	Ctrl+F4	exit the window

11.3.3. The context-sensitive mouse menu

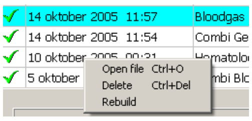

Open file	Ctrl+O	open file
Delete	Ctrl+Del	
Rebuild		

11.3.4. De status bar

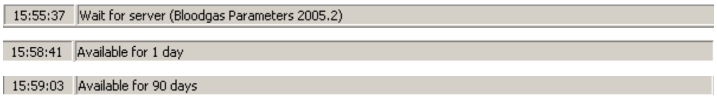

The status bar is self-explanatory

11.4. Reports; E-mail with hyperlinks

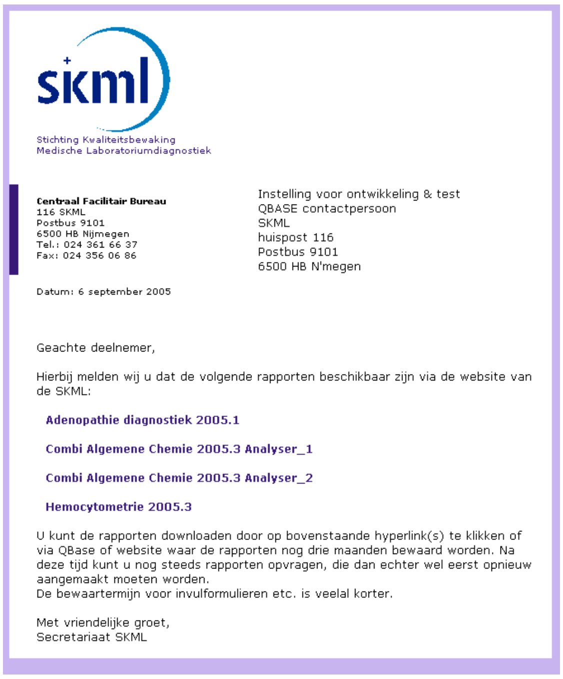

Stichting Kwaliteitsbewaking
Medische Laboratoriumdiagnostiek

Centraal Facilitair Bureau
116 SKML
Postbus 9101
6500 HB Nijmegen
Tel.: 024 361 66 37
Fax: 024 356 06 86

Instelling voor ontwikkeling & test
QBASE contactpersoon
SKML
huispost 116
Postbus 9101
6500 HB N'megen

Datum: 6 september 2005

Geachte deelnemer,

Hierbij melden wij u dat de volgende rapporten beschikbaar zijn via de website van de SKML:

Adenopathie diagnostiek 2005.1

Combi Algemene Chemie 2005.3 Analyser_1

Combi Algemene Chemie 2005.3 Analyser_2

Hemocytometrie 2005.3

U kunt de rapporten downloaden door op bovenstaande hyperlink(s) te klikken of via QBase of website waar de rapporten nog drie maanden bewaard worden. Na deze tijd kunt u nog steeds rapporten opvragen, die dan echter wel eerst opnieuw aangemaakt moeten worden.
De bewaartermijn voor invulformulieren etc. is veelal korter.

Met vriendelijke groet,
Secretariaat SKML

Paticipants receive an e-mail with hyperlinks to download the reports.
These e-mails are send after releasing of the reports.
This function of the report server combines more hyperlinks into one e-mail if possible.

This service is not a function of QBase. It is only mentionned here because of the links to QBase.

12. Statistics

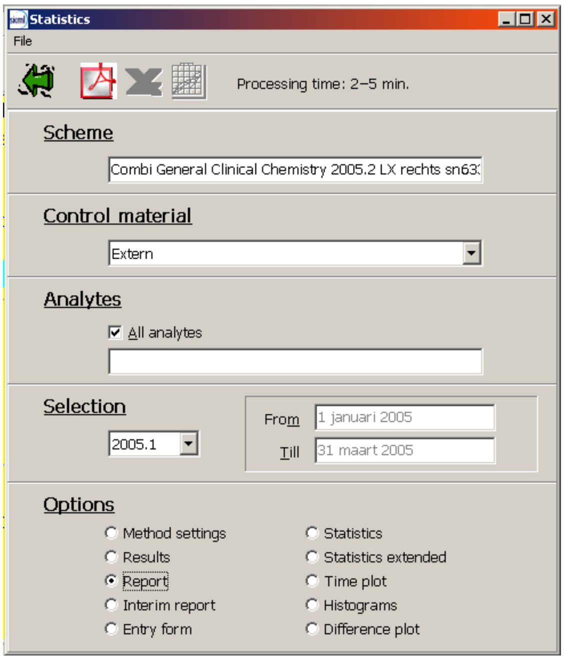

Various summaries can be downloaded via the **Statistics** window.
A pre-selection of the following jointly determine which options will be available:

- **Scheme** (in the main window).
- **Control material** (external control material or one of the internal control materials).
- **Analytes** (all analytes or a specific analyte).
- **Selection** (either a survey or **Period…**, where the dates can be entered in the **From** and **Till** fields).

Next, select one of the available summaries in the **Options** section.

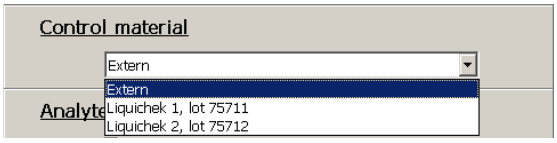

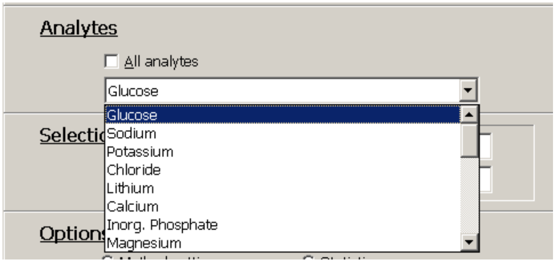

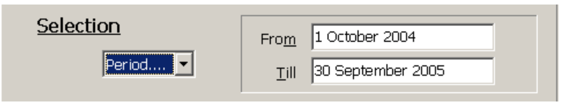

The pre-selection of the following jointly determine which options will be available:

- **Scheme** (in the main window)
- **Control material** (external or one of the internal control materials)
- **Analytes** (all or a specific analyte)
- **Selection** (either a survey or a **Period…** where the dates can be entered in the **From** and **Till** fields).

Next, select one of the available summaries in the **Options** section.

Comment:
Three file formats are available for the output file:

- *An Adobe Acrobat file*
- *An MS Excel file*
- *A graph displayed on the monitor*

Some summaries are only available as a PDF file and others are only available as an Excel file. The button concerned will be activated if the format is available.

12.1. Examples of summaries

The SKML provides various types of summaries.
Examples are listed below.
An explanation of all statistic presentations is also available in a downloadable file on the website of the SKML; just add this link in your browsers address field
http://www.skml.nl/dl/SKML_Graphical_Interpretation_and_tables.pdf
or go to our website (www.skml.nl), switch to the English pages and click the "Downloads"-button.

12.1.1. Method settings

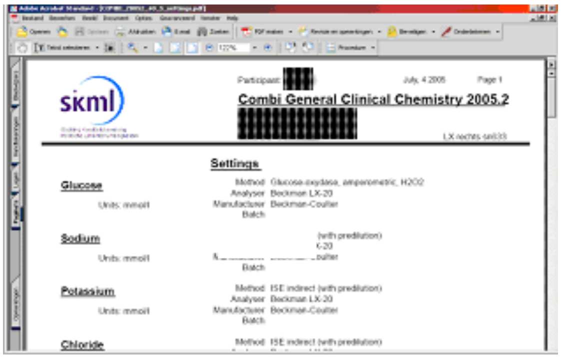

The summary as an Adobe PDF file.

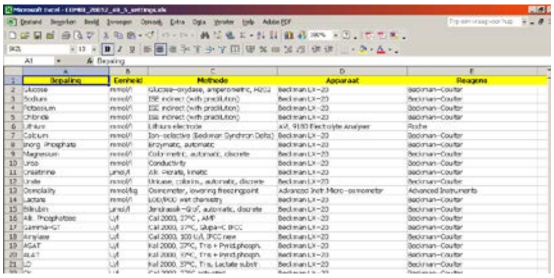

The summary as an MS Excel file.

12.1.2. Results

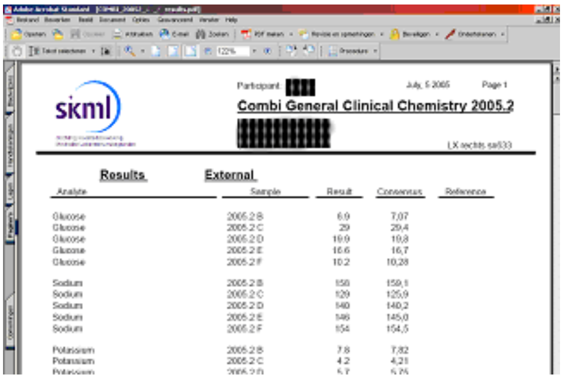

A list of results as a PDF file.

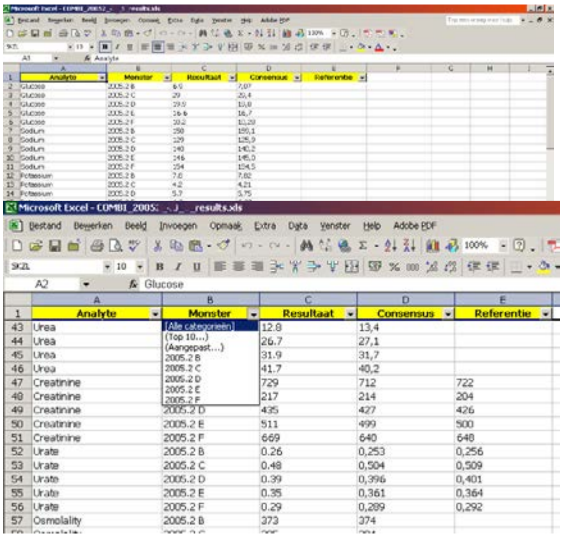

A list of results as an MS Excel file, with the Auto Filter function turned on.

12.1.3. Reports

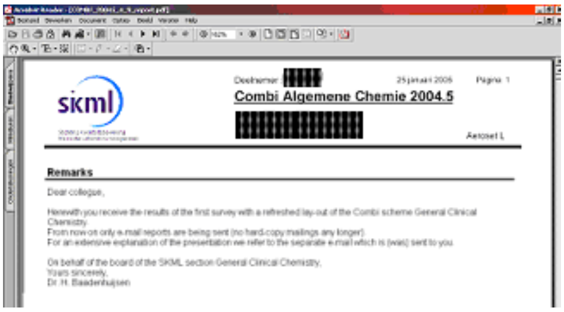

A standard report as an Adobe Acrobat PDF file.

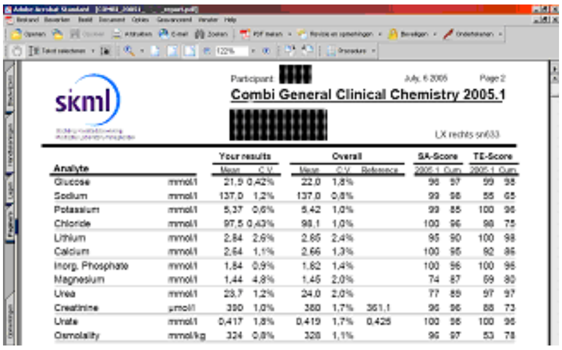

The effect of the **Precision** field in the **Settings** window on the accuracy of the average value:

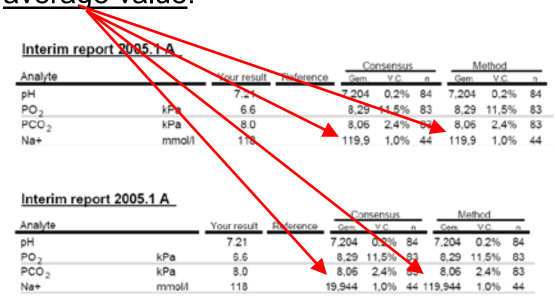

In the first example, the default SKML precision value of 1 is used and in the second example, a precision value of 3 is used.

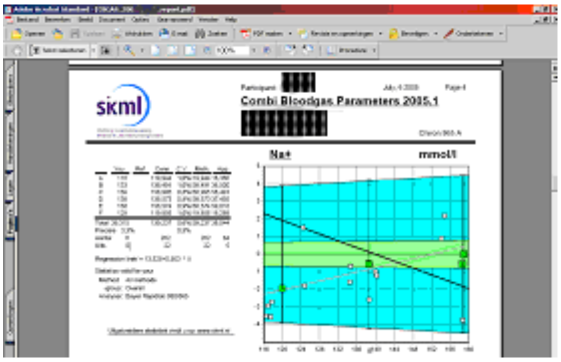

A difference plot.

12.1.4. Interim report

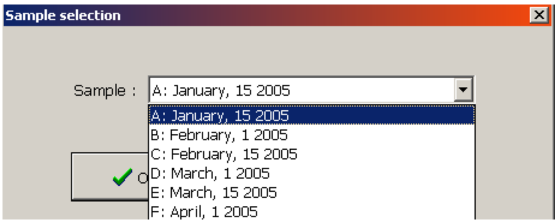

An interim report is only available as a PDF file. The window shown opposite will be displayed after selecting the report and clicking the button. A sample can then be selected.
Next, click the OK button.

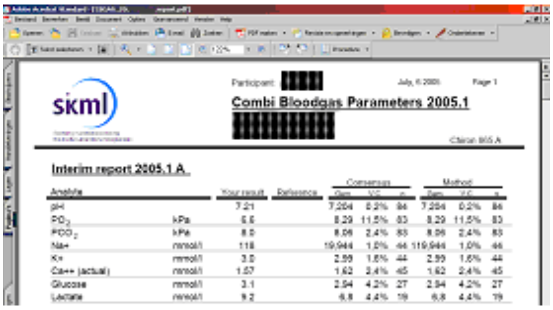
Participant [redacted]

Page 1

Combi Bloodgas Parameters 2005.1

[redacted]

Interim report 2005.1 A

Analyte		Your result	Reference	Consensus Gem	VC	n	Method Gem	VC	n
pH		7.21		7.204	0.2%	84	7.204	0.2%	84
PO_2	kPa	6.6		8.29	11.5%	83	8.29	11.5%	83
PCO_2	kPa	8.0		8.06	2.4%	83	8.06	2.4%	83
Na+	mmol/l	118		119.944	1.0%	44	119.944	1.0%	44
K+	mmol/l	3.0		2.99	1.6%	44	2.99	1.6%	44
Ca++ (actual)	mmol/l	1.57		1.62	2.4%	45	1.62	2.4%	45
Glucose	mmol/l	3.1		2.94	4.2%	27	2.94	4.2%	27
Lactate	mmol/l	9.2		6.8	4.4%	19	6.8	4.4%	19

An example of an interim report.

12.1.5. Data entry form

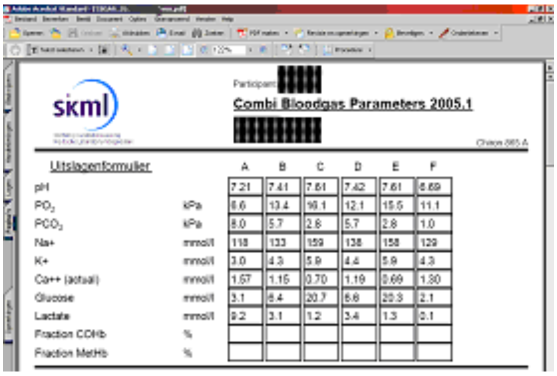
Participant [redacted]

Combi Bloodgas Parameters 2005.1

[redacted]

Uitslagenformulier

		A	B	C	D	E	F
pH		7.21	7.41	7.61	7.42	7.61	6.69
PO_2	kPa	6.6	13.4	16.1	12.1	15.5	11.1
PCO_2	kPa	8.0	5.7	2.8	5.7	2.8	1.0
Na+	mmol/l	118	133	159	138	158	129
K+	mmol/l	3.0	4.3	5.9	4.4	5.9	4.3
Ca++ (actual)	mmol/l	1.57	1.15	0.70	1.19	0.69	1.30
Glucose	mmol/l	3.1	6.4	20.7	6.8	20.3	2.1
Lactate	mmol/l	9.2	3.1	1.2	3.4	1.3	0.1
Fraction COHb	%						
Fraction MetHb	%						

An example of a data entry form is shown opposite.
If this is downloaded before the results have been entered, it can be used as a work list.
After entering the results, they can be printed on the list.

12.1.6. Statistics

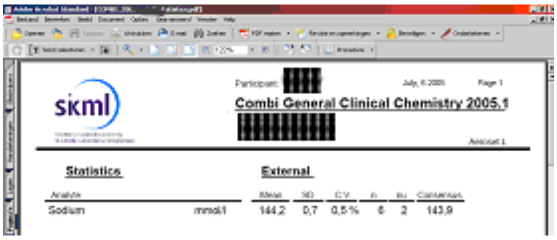

An example of external statistics and internal statistics per analyte are given opposite.

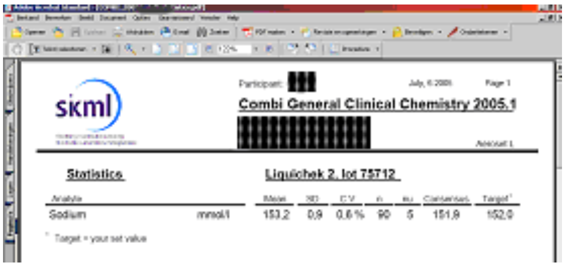

12.1.7. Detailed statistics

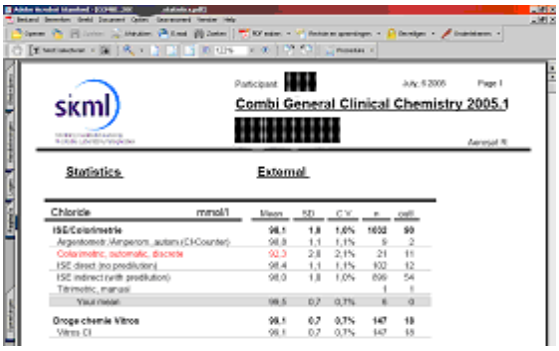

An example of detailed statistics, either per analyte or for all analytes, is given opposite.

The methods given in red are statistically removed methods.

12.1.8. Time diagram

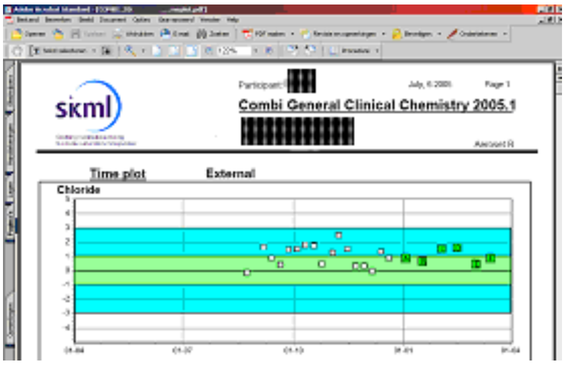

If “All Analytes” is selected for **Analytes**, then all the time plots will be displayed in order, one below the other.

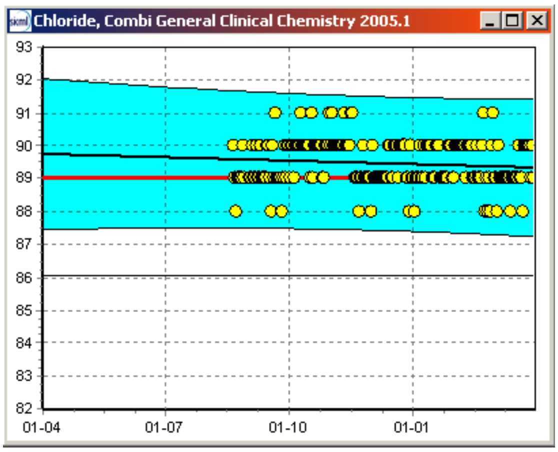

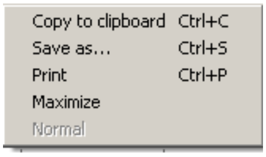

If only one analyte is selected, then the 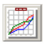 button (Graph Ctrl+G) will be added to the tool bar. The graph shown opposite will be displayed when this button is clicked.

The tool bar and/or menu structure have been omitted from this time diagram on purpose, so that it is possible to *maximize the window whilst maintaining the clarity*. This makes it very suitable for use in presentations.
Extra functions are also available via the right mouse button:

- Copy time diagram to clipboard
- Save as WMF file
- Print time diagram
- Maximize/Normal

This makes it possible to use a diagram in, for example, (PowerPoint) publications.

12.1.9. Histograms

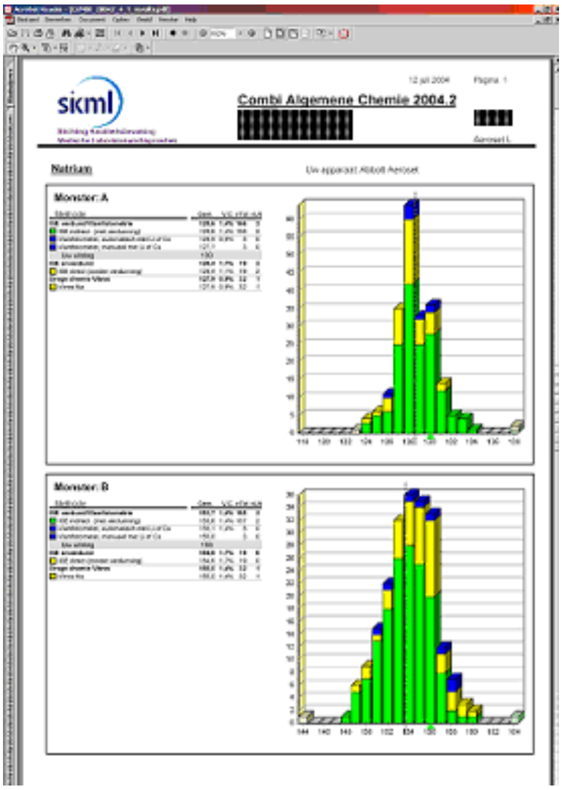

This is an example of a histogram. Besides the graphic information, a list with statistical data of the methods used will also be given.

12.1.10. Difference plots

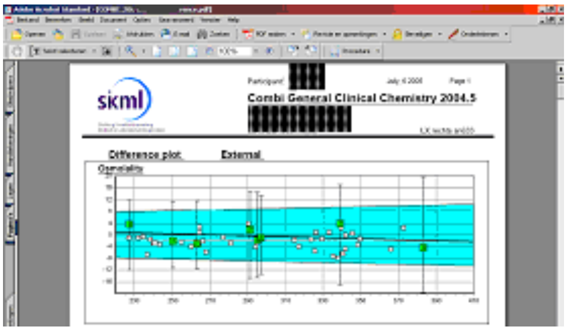

An example of a difference plot.

13. Appendix

13.1. Structure of the QBase import file

Every QBase text file (ASCII, with a **TXT** extension) is composed of the following lines. (The **QBF** extension from previous versions of QBase is still supported.)

QBimport.txt - Kladblok
Bestand Bewerken Opmaak Help

```
SKML COMBI 10
K Dit is een kommentaarregel
M 1 10925 Biorad, Liquichek 1,  lot 75231
M 2 10926 Biorad, Liquichek 2,  lot 75232
SE 1 5 03-11-03 12:41
R 0 105
SE 2 5 10-11-03 08:05
R 0 139
SE 3 5 17-11-03 08:11
R 0 123
SE 4 5 24-11-03 08:13
R 0 155
SE 5 5 01-12-03 08:08
R 0 115
SE 6 5 08-12-03 08:11
R 0 149
SI 1 5 01-11-03 05:57
R 0 127
SI 1 5 01-11-03 14:39
R 0 127
SI 1 5 01-11-03 14:59
R 5 5,8
SI 1 5 02-11-03 03:41
R 0 123
SI 1 5 02-11-03 04:38
R 0 123
SI 2 5 01-11-03 05:57
R 0 155,0
SI 2 5 01-11-03 14:39
R 0 155,0
SI 2 5 01-11-03 14:59
R 5 18,3
SI 2 5 02-11-03 03:41
R 2 2,91
SI 2 5 02-11-03 04:06
R 0 151,0
```

An example of an import file:

- The title (**SKML)** ("SKZL" is also still accepted)
- A comment line (**K**) (optional)
- Material line(s) (**M**)
- External sample line(s) (**SE**)
- Accompanying result lines (**R**)
- Internal sample line(s) (**SI**)
- Accompanying result lines (**R**)

See the following paragraphs for a description of the separate lines.

Comments:
The **order** of the lines in an import file is **not arbitrary**:

- The first line must always be the *title*.
- The second line is a *comment line* (*for your own use; QBase ignores this line*).
- If results from internal control material are imported, the descriptive lines of that internal sample material will then follow.
  The lines for the internal sample material only have to be defined once in the file. The position is not so important, as long as they are positioned before the first SI line which refers to them. To describe them once, at the beginning of the file, immediately after the comment line is the clearest method.
- The lines of the external samples, with the accompanying result records, then follow.
- These are then followed by the lines for the internal samples, with the accompanying result records.

13.1.1. Title

The title has the syntax **SKML [abbreviated name of scheme] [participant number]**
Where:

- **SKML** is fixed text which identifies the title (SKZL is still supported).
- **Scheme name** is the abbreviated name of the scheme (see Cluster properties).
- **Participant number** is your unique participant number (optional).

The title may only be given once and must be the first line.

13.1.2. Comment line

The syntax for the comment line is: **K [comment]**
Where:
- **K** is fixed, identifying text.
- The comment.

Comment:
This line has become unnecessary, but can still be used for your own comment. QBase ignores this line.

13.1.3. Specimen line

The syntax of the material lines is:
M [control material serial number] [control material ID number] [control material name]
Where:
- The **control material serial number** is the serial number which is used to link the control concerned to the accompanying **SI** line(s).
- The **control material ID number** is the identification number which SKML has assigned to the control material concerned.
- The **control material name** is optional and does not necessarily have to be included in the import file.

13.1.4. External sample line

The syntax for the external sample lines is:
SE [external sample] [cluster number] [date/time]
Where:
- **External sample** is the number or letter which identifies the external sample.
- The **cluster number** contains the number of the cluster which the data comes from or to which the data must be added.
- Finally, **date/time** contains the date and time (the time is optional) of the sample concerned. If the time is not identified with the import, then the time 00:00 will automatically be created for the sample. The time 00:00 will not be displayed anywhere.

This line is followed by a number of **R** type lines in which the result of the analyte in that sample is given for each analyte.

13.1.5. Result line

The syntax of the result lines is:
R [analyte] [result]
Where:
- **Analyte** is the code number of the analyte according to the SKML code system.
- **Result** contains the result as saved in the database.

13.1.6. Internal sample line

The syntax for the internal sample lines is:
SI [control material serial number] [cluster number] [date/time]
Where:

- The **control material serial number** is the number of the control material. (This is the link to the accompanying control material. Also see the description for the **M** line.)
- The **cluster number** contains the number of the cluster which the data comes form or to which the data must be added.
- Finally, **date/time** contains the date and time (the time is optional) of the sample concerned. If the time is not identified with the import, then the time 00:00 will automatically be created for the sample.

14. Installation

Go to the SKML website (http://www.skml.nl) and click the 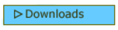

button. The Download page contains a hyperlink for downloading the set-up file.
Start the installation once the download has been completed.

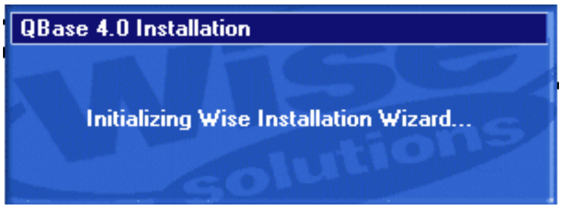

The installation of QBase uses the Wise Solutions installer.
A number of messages will be displayed after the window shown opposite.

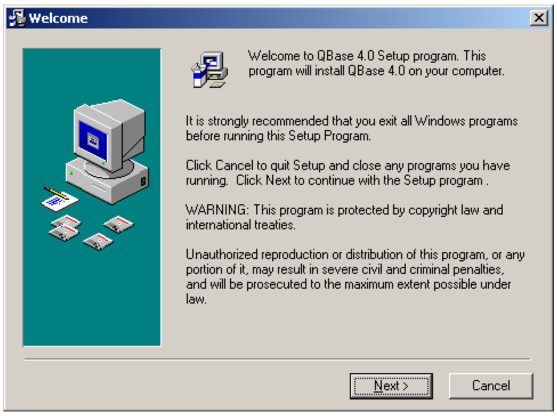

Click 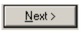

.

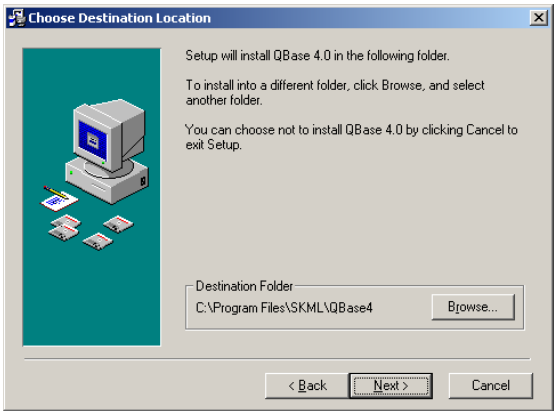

This window explains that QBase will be installed in the folder "C:\Program Files\SKML\QBase4", unless you choose a different location by clicking the 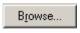

button.
It is also possible to install QBase on a network disk.
If necessary, contact the help desk for more information.

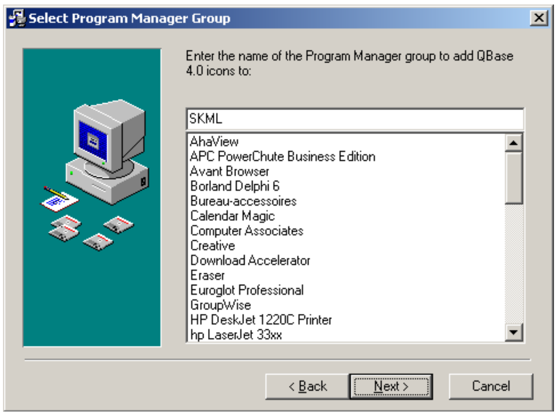

The installation program will create an SKML program group in which the shortcut to the program will be placed.
Click 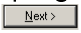

.

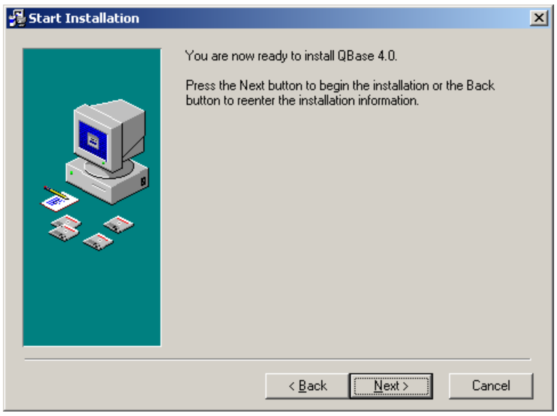

This window is self-explanatory.
Click [Next >].

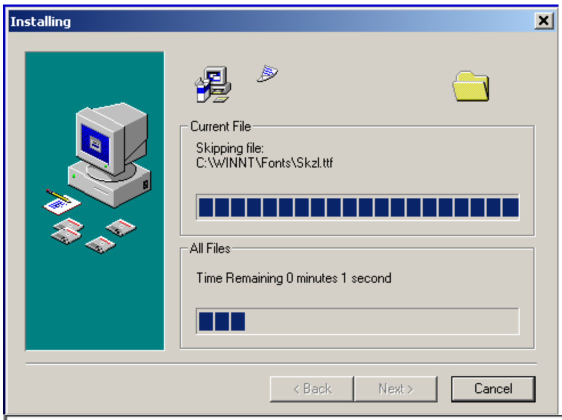

This screen will display the progress of the installation.
The install.log file in the folder in which QBase4 is installed tells you where all the files have been installed.

```
***  Installation Started 04/13/2004 14:52  ***
Title: QBase 4.0 Installation
Source: C:\Documents and Settings\Administrator\Bureaublad\SetupQB4.exe
Made Dir: C:\Program Files\SKML
Made Dir: C:\Program Files\SKML\QBase4
File Copy: C:\Program Files\SKML\QBase4\UNWISE.EXE
RegDB Key: Software\Microsoft\Windows\CurrentVersion\Uninstall\QBase 4.0
RegDB Val: QBase 4.0
RegDB Name: DisplayName
RegDB Root: 2
RegDB Key: Software\Microsoft\Windows\CurrentVersion\Uninstall\QBase 4.0
RegDB Val: C:\PROGRA~1\SKML\QBASE4\UNWISE.EXE C:\PROGRA~1\SKML\QBASE4\INSTALL.LOG
RegDB Name: UninstallString
RegDB Root: 2
File Copy: C:\WINNT\System32\midas.dll
Date/Time: Following file not copied.
 File Copy: C:\WINNT\Fonts\Skzl.ttf
File Copy: C:\Program Files\SKML\QBase4\QBase4.exe
Made Dir: C:\Documents and Settings\Administrator\Menu Start\Programma's\SKML
Shell Link: C:\Documents and Settings\Administrator\Menu Start\Programma's\SKML\QBase4.lnk
User Rights: Admin
```

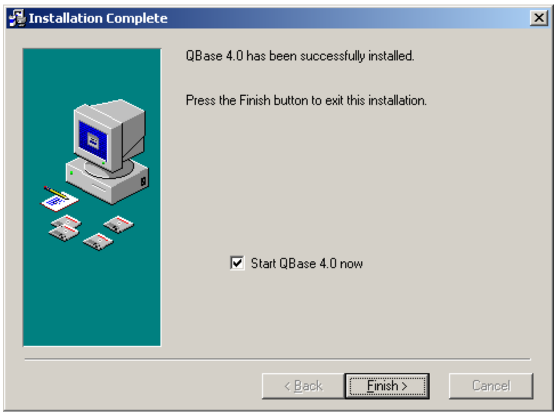

Unless you unselect the check box, QBase will start once the installation has been completed.

Comments for the installation:

- Some system administrators have made alterations to Windows, so that it is not possible for a user to install software. In that case, please contact your system administrator.
- An identification key is created as part of the installation procedure when the program is started up for the first time. This is done automatically and you will probably not notice anything.
- The program can be started from a network without any problems. The system administrator must, however, make sure that the MIDAS.DLL file and the SKZL.TTF font are installed.

14.1. New version of QBase

14.1.1. Updating automatically

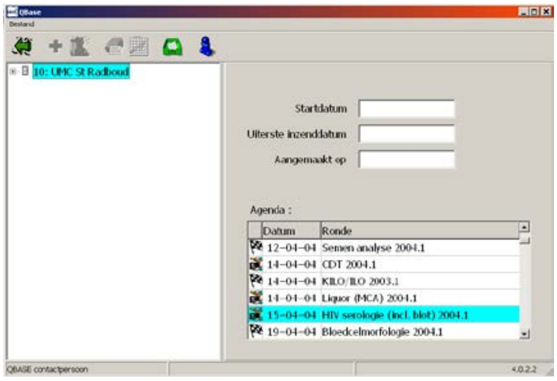

If, after logging in to QBase, the button is visible in the tool bar, a new version of the program is available and it may be possible to update QBase automatically. Click the button to start the download and installation of the new version.

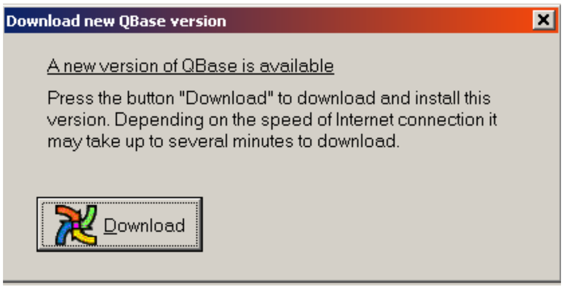

Once the button has been clicked, QBase will first check whether the user of the PC has sufficient rights on that PC to copy files. If so, the window shown opposite will be displayed. Clicking the Actualiseren button will start the actual download.

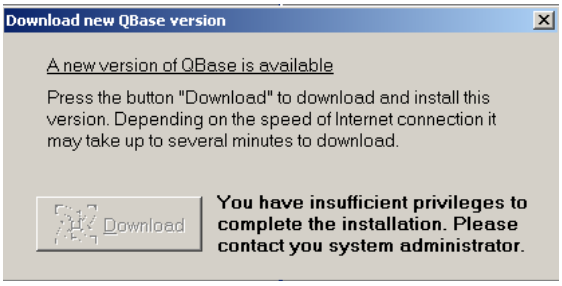

If the system administrator (of the department or the hospital) has limited the individual user rights to an extent that it is no longer possible to copy files, then it is only possible for QBase to be updated by the system administrator.
If the program has been successfully downloaded and installed, QBase will start again and display the Login window.

Comment:
If QBase has determined that the user can download and install a new version, then this is not a guarantee that the installation will be successful. For example, it must also be possible to have a program started by another program on the PC concerned.
The description of the method followed for updating QBase is as follows:

- The user sees that a new version is available and clicks the button and then the button.
- QBase will download the file of the new version and save it under the name "QBase_new.exe" in the same folder as where the program is currently located.
- QBase will start Rename_QB and then close.
- Rename_QB will remove QBase.exe.
- Rename_QB will rename QBase_new.exe as QBase4.exe.
- Rename_QB will start QBase.exe and then close.

If it now appears that nothing is happening after the button has been clicked, or the update procedure is not successfully completed (the Login window should be displayed again), please contact the CFS (Central Facility Service) of SKML. (☎+31 24 361 66 37).

14.2. Information for system administrators

QBase saves a number of registry keys during installation and the first time it is used:

- HKCU\Software\Microsoft\Windows\Currentversion\Explorer\Menuorder\StartMenu\Programs\Skml
- HKLM\software\\Microsoft\Windows\Currentversion\installer\userdata\S-1-5-18\Products\381…..
- HKLM\software\\Microsoft\Windows\Currentversion\Uninstall\QBase4.0

QBase4 is a program which has been developed by the Dutch Foundation for Quality Assessment in Clinical Laboratories (SKML) and which participants of the quality control schemes can use to enter their data into the SKML database.
QBase4 is a relatively small application which can be installed anywhere in a network. Some information for system administrators is given below:

- Three files are installed on the computer during installation:
  - QBase4.exe (the program)
  - Midas.dll (contains all the information for transforming database assignments into XML/http)
  - SKZL.ttf (the font used to display symbols, such as β, γ en 10^9 IgG_1).
- Most PCs which run under Windows XP and Windows NT will probably be protected so that the user must have administrator rights in order to install the MIDAS.dll file in the system32 directory and the SKZL.ttf file in the fonts directory.
- For a default installation on a PC, the program is installed in C:\Program Files\SKML\QBase4, but QBase can be installed in any (network) location.

- QBase communicates using the http protocol via port 80. Proxy servers may be configured too strictly for this. QBase includes functionality which allows system administrators to configure the proxy.
- When the user starts QBase4, the program connects to the SKML web server which is based on the SOAP-protocol and which is located in Nijmegen, the Netherlands.
- The user can configure the program so that it will be necessary to log in every time the program is started, after a fixed time or never.
- After logging in, the user is recorded in the central database based on a key. This key is saved once in the HKCU registry.
- The connection with the central database will always be created by the participant, never by the central database.

15. Reference words

A
Agenda
downloading options 23
Altering login data 12
Altering login name 12
Altering login password 13
Analyte settings 32
analyser 32
method 32
C
Clinical interpretation 9
Cluster 8, 28
chain 8
importing results 40
new cluster
create 28
history
cluster chains 29
no history 28
name analysis environment 28
properties 40
settings 28, 34
(de-)-activate analyte 35
analyte NOT supported 35
analyte order 35
analyte settings 32
analyser 32
method 32
calibrator 35
expiration date 35
internal control materials 35
lot number 35
methods 34
plausibility 35
precision 35
producer 35
remarks 40
sample dates 29
type 'Combi' 33
type 'External' 33
unique code numbers 40
Connecting to TEST-database 13
Contact person 7
Contents 2
D
Data entry form 27
Definite result 9
Definitions 7
clinical interpretation 9
cluster 8
cluster chain 8
contact person 7
evaluation 9
hospital 7
institute 7
participant 7
production centre 8
provisional result 9
result
definite result 9
score 9
result 9
sample 8
scheme 7
scheme coordinator 8
subscription 7
survey 8
survey distributor 8
Definitions☹detailled) questionnaire 9
E
Evaluation 9
G
Getting reports 51
H
Hospital 7
I
Importing results 40, 47
Installation
QBase 64
download software 64
Welcome 64
Institue
name 16
Institute 7
Instruction manual
buttons 7
design menu options 6
design toolbar options 6
hintinfo 7
menu options 7
mouse button 7
reading instructions 6
default fonts 6
exclamation mark 6

references 6
shortcut keys 7
Internal control
activate analytes 37
changing order internal controls..... 39
exchanging internal controls 38
limit values...................................... 39
lot number 36
manufacturer 36
not included in list 36
request confirmation 39
select next control
include selected analytes 37
selecting 36
support unlimited # internal controls
.... 37
L
Login-window 10
altering login data 12
altering login password 13
error.. 12
forgotten password 12
logging in automatically 11
options.. 11
remember data 11
M
Messages 24
reading a message 25
writing a message........................... 25
N
Node-sign 16
P
Participant.. 7
Presentations
statistics
time plot 59
Production centre................................. 8
Program settings 20
decimal sign 20
proxy bypass 21
proxy server.................................... 21
proxy settings 21
S.D.value 20
saving Adobe pdf files.................... 21
Provisional result 9
Q
QBase
automatic download........................ 66
test user rights 66
user right limitid by administrator 66
automatic notifications 13
connecting to TEST-database........ 13
donwloading automatically 14
blocking too old versions 15
check privilages 14
history
QBaseWWW 6
version 1-4 6
import file
comment line 61, 62
external sample line 61, 62
internal sample line 61, 63
result line 61, 62
specimen line 61, 62
structure 61
title line 61
main window 16
agenda 16
deadline 16
start date 16
starting QBase 10
special functions 10
Questionnaire (detailed)...................... 9
R
Remarks 44
Report management window............ 52
Reports 26
e-mail with hyperlink(s) 53
Result 9
Result entry 41
by analyte 46
by sample 46
Combi 41
External 41
import error 49
import function
error log 49
import function diagnosis 49
importing results 47
internal sample 47
plausibility limits
external samples 43
internal samples 43
remarks 44
result field deactivated 47
select import file 48
type 'combi' 45
type 'external' 45
validate survey 44
Result field deactivated 47
S
Sample 8

Scheme 7
coordinator 8
type 'Combi' 6
cluster settings 33
type 'External' 6
cluster settings 33
Score 9
SKML messages 24
reading a message 25
writing a message 25
SOAP-proptocol 68
Statistics 58
by scheme 54
by survey 54
data entry form 57
difference plot 54, 56
diffference plot 60
histogram 59
histograms 54
interim report 57
method settings 54, 55
precision 56
report 54, 56
results 54, 55
select
analyte 54
control material 54
survey or period 54
statistics detailed 58
time plot 54, 58
presentations 59
various summaries 54
Subscription 7
Survey 8
Survey distributor 8
T
TEST-database
connecting to 13
Time plot
presentaties 59
V
Validate
survey 44
W
Website
www.skml.nl 6
Windows versions
98 and later 6
