# Supplementary material for: First international external quality assessment scheme of nucleic acid amplification tests for the detection of Schistosoma and soil-transmitted helminths, including Strongyloides: A pilot study
Source: PLoS Negl Trop Dis. 2020 Jun 16;14(6):e0008231. doi: 10.1371/journal.pntd.0008231 (PMC7319349; doi:10.1371/journal.pntd.0008231)
Supplement: S2 File — (PDF) [file pntd.0008231.s002.pdf]

S2 File. INSTRUCTIONS ACCOMPANYING THE PANEL ON HOW TO REMOVE ETHANOL FROM THE STOOL SAMPLES

SKML Helminths External Quality Assessment Scheme (SHEQAS)

Rotterdam, the Netherlands, July 6th 2017

Dear sir/madam,

Your laboratory participates as an expert-laboratory in the SKML Helminths External Quality Assessment Scheme (SHEQAS) for the detection of helminths by DNA amplification techniques.

The responsible coordinator in your laboratory has received information by email about the number and type of specimens that your laboratory should receive. Please find enclosed the stool samples in ethanol solution (500 µl) and purified DNA samples (50 µl). Please check whether you have received the proper set of samples.

Prior to DNA extraction from the stool samples, pre-analysis processing is required to remove the solvent ethanol (see below for description of the procedure).

The tubes with the purified DNA material can be centrifuged with a brief spin to concentrate the 50µl volume at the bottom of the tube. Subsequently the DNA solution can directly be added to the PCR mixture. It can thus be used as if it is the “DNA elution solution” produced by your regular DNA extraction-method.

Examine all supplied specimens for the presence of Soil Transmitted Helminths (*Trichuris trichiura*, *Ascaris* spp and hookworm species), *Strongyloides stercoralis* and if available *Schistosoma* spp. (so examine all specimens for all targets).

Results should be registered in the excel “SHEQAS_2017_reference” sheet, which has been by email sent to the coordinator of your laboratory. For each sample the results for each target should be provided qualitatively (positive, negative, failed, amplification inhibited) and if positive the Cq (Ct or Cp) value should be reported. When all examinations have been performed the completed result sheet can be send by email to j.vanhellemond@erasmusmc.nl and r.koelewijn@erasmusmc.nl of the central SHEQAS sample preparation laboratory.

The deadline for submission of the results is **September 1st, 2017**.

In case you have any questions, please feel free to contact us by mail and we will answer your questions as soon as possible.

Best regards,

Rob Koelewijn & Dr. Jaap van Hellemond
Dept. Medical Microbiology & Infectious Diseases
Erasmus University Medical Centre & Harbour Hospital, Rotterdam, the Netherlands
Email: r.koelewijn@erasmusmc.nl and j.vanhellemond@erasmusmc.nl

Pre-analysis processing of stool samples *(please perform exactly as described)*

1. transfer 250 µl of the ethanol-stool suspension to a clean eppendorf tube
2. centrifuge for 1 minute (10.000 rpm)
3. remove the ethanol with a micro-pipette and vortex pellet
4. add 1000 µl of PBS (or 0.9 % NaCl) and vortex
5. centrifuge for 1 minute (10.000 rpm)
6. remove the PBS (or 0.9 % NaCl) with micro-pipette
7. resuspend pellet by vortexing in the suspension fluid you would normally use to make fecal suspensions in your DNA isolation procedure
