# Supplementary material for: First international external quality assessment scheme of nucleic acid amplification tests for the detection of Schistosoma and soil-transmitted helminths, including Strongyloides: A pilot study
Source: PLoS Negl Trop Dis. 2020 Jun 16;14(6):e0008231. doi: 10.1371/journal.pntd.0008231 (PMC7319349; doi:10.1371/journal.pntd.0008231)
Supplement: S3 File — (PDF) [file pntd.0008231.s003.pdf]

S3 File. THE TARGET VALIDATION OF THE STOOL AND DNA PANEL BY EXPERT LABORATORIES

Each table shows the results of the nucleic acid amplification tests of one sample by the six expert laboratories. The first row of each table lists the sample name and the different targets. The results of the quintuplicates analyzed by a single laboratory are shown in the white rows with negative results indicated by 0 and positive results documented by the Cq value. The results of the laboratories that analyzed a single aliquot are shown in the grey rows, with a positive results indicated by 1 and a negative result indicated by 0.
The criteria used to define a sample as positive, negative or educational for a certain target are listed below.

Positive	1. All quintuplicates positive for that target<br>2. Standard deviation (SD) Cq quintuplicates < 2<br>3. Found positive by all other expert laboratories for that target
Negative	1. All quintuplicates negative for that target<br>2. Found negative by all other expert laboratories for that target
Educational	Not positive and not negative for that target

The results of the target validation are shown in the last row of each table.
Legend: ST, stool sample; DNA, DNA sample; Exp. Lab., expert laboratory; SD, standard deviation; nd, not determined.

Stool panel

ST1	*Ascaris lumbricoides*	*Trichuris trichiura*	*Necator americanus*	*Ancylostoma duodenale*	*Strongyloides stercoralis*	*Schistosoma mansoni*
Exp. Lab. 5	0	0	0	0	0	0
	0	0	0	0	0	0
	0	0	0	0	0	0
	0	0	0	0	0	0
	0	0	0	0	0	0
Mean						
SD						
Exp. Lab. 1	0	0	0	0	0	0
Exp. Lab. 2	0	0	0	0	0	0
Exp. Lab. 3	0	0	0	0	nd	nd
Exp. Lab. 4	0	0	0	0	0	0
Exp. Lab. 6	nd	nd	nd	nd	0	0
	Negative	Negative	Negative	Negative	Negative	Negative

ST2	*Ascaris lumbricoides*	*Trichuris trichiura*	*Necator americanus*	*Ancylostoma duodenale*	*Strongyloides stercoralis*	*Schistosoma mansoni*
Exp. Lab. 1	0	0	0	0	22.3	0
	0	0	0	0	22.6	0
	0	0	38.0	0	21.4	0
	0	0	0	0	22.7	0
	0	0	0	0	21.9	0
Mean					22.2	
SD					0.47	
Exp. Lab. 2	0	0	0	0	1	0
Exp. Lab. 3	0	0	0	0	nd	nd
Exp. Lab. 4	1	0	0	0	1	0
Exp. Lab. 5	0	0	0	0	0	0
Exp. Lab. 6	nd	nd	nd	nd	1	0
	Educational	Negative	Educational	Negative	Educational	Negative

ST3	*Ascaris lumbricoides*	*Trichuris trichiura*	*Necator americanus*	*Ancylostoma duodenale*	*Strongyloides stercoralis*	*Schistosoma mansoni*
Exp. Lab. 2	22.6	0	0	0	0	0
	23.5	31.7	0	0	0	0
	22.7	33.6	0	0	0	0
	23.3	0	0	0	0	0
	23.0	0	0	0	0	0
Mean	23.0					
SD	0.34					
Exp. Lab. 1	1		0	0	0	0
Exp. Lab. 3	1		0	0	nd	nd
Exp. Lab. 4	0		0	0	0	0
Exp. Lab. 5	1		0	0	0	0
Exp. Lab. 6	nd		nd	nd	0	0
	Educational	Educational	Negative	Negative	Negative	Negative

ST4	*Ascaris lumbricoides*	*Trichuris trichiura*	*Necator americanus*	*Ancylostoma duodenale*	*Strongyloides stercoralis*	*Schistosoma mansoni*
Exp. Lab. 2	0	29.3	0	0	0	0
	0	30.6	0	0	0	0
	0	28.9	0	0	0	0
	0	29.4	0	0	0	0
	0	29.1	0	0	0	0
Mean		29.5				
SD		0.60				
Exp. Lab. 1	0	1	0	0	0	0
Exp. Lab. 3	0	1	0	0	nd	nd
Exp. Lab. 4	0	1	0	0	0	0
Exp. Lab. 5	0	1	0	0	0	0
Exp. Lab. 6	0	nd	0	0	0	0
	Negative	Positive	Negative	Negative	Negative	Negative

ST5	*Ascaris lumbricoides*	*Trichuris trichiura*	*Necator americanus*	*Ancylostoma duodenale*	*Strongyloides stercoralis*	*Schistosoma mansoni*
Exp. Lab. 2	0	0	30.5	0	0	0
	0	0	27.3	0	0	0
	0	0	28.4	0	0	0
	0	0	28.9	0	0	0
	0	0	30.9	0	0	0
Mean			29.2			
SD			1.34			
Exp. Lab. 1	0	0	1	0	0	0
Exp. Lab. 3	0	0	1	0	nd	nd
Exp. Lab. 4	0	0	1	0	0	0
Exp. Lab. 5	0	0	1	0	0	0
Exp. Lab. 6	nd	nd	nd	nd	nd	nd
	Negative	Negative	Positive	Negative	Negative	Negative

ST6	*Ascaris lumbricoides*	*Trichuris trichiura*	*Necator americanus*	*Ancylostoma duodenale*	*Strongyloides stercoralis*	*Schistosoma mansoni*
Exp. Lab. 1	29.2	0	28.6	0	0	0
	28.0	0	29.5	0	0	0
	28.1	0	27.8	0	0	0
	28.3	0	28.4	0	0	0
	29.2	0	29.0	0	0	0
Mean	28.6		28.7			
SD	0.54		0.55			
Exp. Lab. 2	1	0	1	0	1	0
Exp. Lab. 3	1	0	1	0	nd	nd
Exp. Lab. 4	1	0	1	0	0	0
Exp. Lab. 5	1	0	1	0	0	0
Exp. Lab. 6	nd	nd	nd	nd	1	0
	Positive	Negative	Positive	Negative	Educational	Negative

ST7	*Ascaris lumbricoides*	*Trichuris trichiura*	*Necator americanus*	*Ancylostoma duodenale*	*Strongyloides stercoralis*	*Schistosoma mansoni*
Exp. Lab. 3	28.8	33.5	0	0	0	nd
	28.8	31.7	0	0	0	nd
	28.9	35.7	0	0	0	nd
	29.4	33.2	0	0	0	nd
	29.6	34.6	0	0	0	nd
Mean	29.1	33.7				
SD	0.33	1.35				
Exp. Lab. 1	1	1	0	0	0	0
Exp. Lab. 2	1	1	0	0	0	0
Exp. Lab. 4	1	1	0	0	0	0
Exp. Lab. 5	1	1	0	0	0	0
Exp. Lab. 6	nd	nd	nd	nd	0	0
	Positive	Positive	Negative	Negative	Negative	Negative

ST8	*Ascaris lumbricoides*	*Trichuris trichiura*	*Necator americanus*	*Ancylostoma duodenale*	*Strongyloides stercoralis*	*Schistosoma mansoni*
Exp. Lab. 3	31.7	0	0	0	0	nd
	32.1	0	0	0	0	nd
	32.9	0	0	0	0	nd
	32.0	0	0	0	0	nd
	0	0	0	0	0	nd
Mean						
SD						
Exp. Lab. 1		1	0	0	0	0
Exp. Lab. 2		1	0	0	0	0
Exp. Lab. 4		1	0	0	0	0
Exp. Lab. 5		0	0	0	0	0
Exp. Lab. 6		nd	nd	nd	0	0
	Educational	Educational	Negative	Negative	Negative	Negative

ST9	*Ascaris lumbricoides*	*Trichuris trichiura*	*Necator americanus*	*Ancylostoma duodenale*	*Strongyloides stercoralis*	*Schistosoma mansoni*
Exp. Lab. 1	34.6	37.6	32.2	0	0	24.5
	32.8	33.5	31.1	0	0	23.4
	34.8	34.4	30.9	0	0	24.4
	33.6	34.2	31.4	0	0	25.2
	33.2	32.4	32.1	0	0	28.4
Mean	33.8	34.4	31.5			25.2
SD	0.77	1.71	0.54			1.71
Exp. Lab. 2	0	1	1	0	0	1
Exp. Lab. 3	1	0	1	0	0	nd
Exp. Lab. 4	1	1	1	0	0	1
Exp. Lab. 5	1	0	1	0	0	0
Exp. Lab. 6	nd	nd	nd	nd	0	1
	Educational	Educational	Positive	Negative	Negative	Educational

ST10	*Ascaris lumbricoides*	*Trichuris trichiura*	*Necator americanus*	*Ancylostoma duodenale*	*Strongyloides stercoralis*	*Schistosoma mansoni*
Exp. Lab. 4	0	30.1	0	0	0	0
	0	29.3	0	0	0	0
	0	32.9	0	0	0	0
	0	31.1	0	0	0	0
	0	30.6	0	0	0	0
Mean		30.8				
SD		1.21				
Exp. Lab. 1	0	1	0	0	0	0
Exp. Lab. 2	0	1	0	0	0	0
Exp. Lab. 3	0	0	0	0	0	nd
Exp. Lab. 5	0	0	0	0	0	0
Exp. Lab. 6	nd	nd	nd	nd	0	0
	Negative	Educational	Negative	Negative	Negative	Negative

ST11	*Ascaris lumbricoides*	*Trichuris trichiura*	*Necator americanus*	*Ancylostoma duodenale*	*Strongyloides stercoralis*	*Schistosoma mansoni*
Exp. Lab. 4	33.3	32.2	33.3	0	0	26.1
	34.0	0	33.7	0	0	27.3
	33.7	33.0	35.5	0	0	26.9
	37.6	0	33.6	0	0	25.9
	33.7	33.5	31.8	0	0	25.3
Mean	34.5		33.6			26.3
SD	1.59		1.18			0.72
Exp. Lab. 1	1		1	0	0	1
Exp. Lab. 2	1		1	0	0	1
Exp. Lab. 3	1		1	0	0	nd
Exp. Lab. 5	1		1	0	0	0
Exp. Lab. 6	nd		nd	nd	0	0
	Positive	Educational	Positive	Negative	Negative	Educational

ST12	*Ascaris lumbricoides*	*Trichuris trichiura*	*Necator americanus*	*Ancylostoma duodenale*	*Strongyloides stercoralis*	*Schistosoma mansoni*
Exp. Lab. 5	34.3	0	22.4	0	0	0
	33.9	0	24.2	0	0	0
	33.8	0	23.4	0	0	0
	33.8	0	25.2	0	0	0
	33.7	0	23.6	0	0	0
Mean	33.9		23.8			
SD	0.21		0.92			
Exp. Lab. 1	1	1	1	0	0	1
Exp. Lab. 2	1	0	1	0	0	1
Exp. Lab. 3	1	0	1	0	0	nd
Exp. Lab. 4	1	1	1	0	0	1
Exp. Lab. 6	nd	nd	nd	nd	0	1
	Positive	Educational	Positive	Negative	Negative	Educational

DNA panel

DNA1	*Ascaris lumbricoides*	*Trichuris trichiura*	*Necator americanus*	*Ancylostoma duodenale*	*Strongyloides stercoralis*	*Schistosoma mansoni*
Exp. Lab. 1	0	0	0	0	26.1	0
	0	0	0	0	26.0	0
	0	0	0	0	26.1	0
	0	0	0	0	26.1	0
	0	0	0	0	26.2	0
Mean					26.1	
SD					0.04	
Exp. Lab. 2	0	0	0	0	1	0
Exp. Lab. 3	0	0	0	0	nd	nd
Exp. Lab. 4	0	0	0	0	1	0
Exp. Lab. 5	0	0	0	0	1	0
Exp. Lab. 6	nd	nd	nd	nd	1	nd
	Negative	Negative	Negative	Negative	Positive	Negative

DNA2	*Ascaris lumbricoides*	*Trichuris trichiura*	*Necator americanus*	*Ancylostoma duodenale*	*Strongyloides stercoralis*	*Schistosoma mansoni*
Exp. Lab. 1	0	0	0	0	32.3	0
	0	0	0	0	32.4	0
	0	0	0	0	32.0	0
	0	0	0	0	32.5	0
	0	0	0	0	32.8	0
Mean					32.4	
SD					0.26	
Exp. Lab. 2	0	0	0	0	1	0
Exp. Lab. 3	0	0	0	0	nd	nd
Exp. Lab. 4	0	0	0	0	1	0
Exp. Lab. 5	0	0	0	0	0	0
Exp. Lab. 6	nd	nd	nd	nd	1	nd
	Negative	Negative	Negative	Negative	Educational	Negative

DNA3	*Ascaris lumbricoides*	*Trichuris trichiura*	*Necator americanus*	*Ancylostoma duodenale*	*Strongyloides stercoralis*	*Schistosoma mansoni*
Exp. Lab. 2	0	0	0	0	0	24.3
	0	0	0	0	0	24.0
	0	0	0	0	0	24.0
	0	0	0	0	0	24.0
	0	0	0	0	0	24.2
Mean						24.1
SD						0.13
Exp. Lab. 1	0	0	0	0	0	1
Exp. Lab. 3	0	0	0	0	nd	nd
Exp. Lab. 4	0	0	0	0	0	1
Exp. Lab. 5	0	0	0	0	0	0
Exp. Lab. 6	nd	nd	nd	nd	0	1
	Negative	Negative	Negative	Negative	Negative	Educational

DNA4	*Ascaris lumbricoides*	*Trichuris trichiura*	*Necator americanus*	*Ancylostoma duodenale*	*Strongyloides stercoralis*	*Schistosoma mansoni*
Exp. Lab. 2	0	0	0	0	0	27.6
	0	0	0	0	0	27.5
	0	0	0	0	0	27.4
	0	0	0	0	0	27.5
	0	0	0	0	0	27.3
Mean						27.5
SD						0.10
Exp. Lab. 1	0	0	0	0	0	1
Exp. Lab. 3	0	0	0	0	nd	nd
Exp. Lab. 4	0	0	0	0	0	1
Exp. Lab. 5	0	0	0	0	0	0
Exp. Lab. 6	nd	nd	nd	nd	0	1
	Negative	Negative	Negative	Negative	Negative	Educational

DNA5	*Ascaris lumbricoides*	*Trichuris trichiura*	*Necator americanus*	*Ancylostoma duodenale*	*Strongyloides stercoralis*	*Schistosoma mansoni*
Exp. Lab. 3	29.3	0	0	0	nd	nd
	28.9	0	0	0	nd	nd
	28.8	0	0	0	nd	nd
	29.2	0	0	0	nd	nd
	29.5	0	0	0	nd	nd
Mean	29.1					
SD	0.26					
Exp. Lab. 1	1	0	0	0	0	0
Exp. Lab. 2	1	0	0	0	0	0
Exp. Lab. 4	1	0	0	0	0	0
Exp. Lab. 5	1	0	0	0	0	0
Exp. Lab. 6	nd	nd	nd	nd	nd	nd
	Positive	Negative	Negative	Negative	Negative	Negative

DNA6	*Ascaris lumbricoides*	*Trichuris trichiura*	*Necator americanus*	*Ancylostoma duodenale*	*Strongyloides stercoralis*	*Schistosoma mansoni*
Exp. Lab. 4	29.3	0	0	0	0	0
	28.9	0	0	0	0	0
	28.8	0	0	0	0	0
	29.2	0	0	0	0	0
	29.5	0	0	0	0	0
Mean	29.1					
SD	0.26					
Exp. Lab. 1	1	0	0	0	0	0
Exp. Lab. 2	1	0	0	0	0	0
Exp. Lab. 3	1	0	0	0	nd	nd
Exp. Lab. 5	1	0	0	0	0	0
Exp. Lab. 6	nd	nd	nd	nd	nd	nd
	Positive	Negative	Negative	Negative	Negative	Negative

DNA7	*Ascaris lumbricoides*	*Trichuris trichiura*	*Necator americanus*	*Ancylostoma duodenale*	*Strongyloides stercoralis*	*Schistosoma mansoni*
Exp. Lab. 5	0	0	21.5	0	0	0
	0	0	21.7	0	0	0
	0	0	21.9	0	0	0
	0	0	22.6	0	0	0
	0	0	22.2	0	0	0
Mean			22.0			
SD			0.39			
Exp. Lab. 1	0	0	1	0	0	0
Exp. Lab. 2	0	0	1	0	0	0
Exp. Lab. 4	0	0	1	0	nd	Nd
Exp. Lab. 5	0	0	1	0	0	0
Exp. Lab. 6	nd	nd	nd	nd	nd	Nd
	Negative	Negative	Positive	Negative	Negative	Negative

DNA8	*Ascaris lumbricoides*	*Trichuris trichiura*	*Necator americanus*	*Ancylostoma duodenale*	*Strongyloides stercoralis*	*Schistosoma mansoni*
Exp. Lab. 5	0	0	25.4	0	0	0
	0	0	25.0	0	0	0
	0	0	24.9	0	0	0
	0	0	25.1	0	0	0
	0	0	25.2	0	0	0
Mean			25.1			
SD			0.17			
Exp. Lab. 1	0	0	1	0	0	0
Exp. Lab. 2	0	0	1	0	0	0
Exp. Lab. 4	0	0	1	0	0	0
Exp. Lab. 5	0	0	1	0	0	0
Exp. Lab. 6	nd	nd	nd	nd	nd	Nd
	Negative	Negative		Negative	Negative	Negative
