# Supplementary material for: First international external quality assessment scheme of nucleic acid amplification tests for the detection of Schistosoma and soil-transmitted helminths, including Strongyloides: A pilot study
Source: PLoS Negl Trop Dis. 2020 Jun 16;14(6):e0008231. doi: 10.1371/journal.pntd.0008231 (PMC7319349; doi:10.1371/journal.pntd.0008231)
Supplement: S4 File — (PDF) [file pntd.0008231.s004.pdf]

S4 File. EXAMPLE OF A HEMQAS REPORT

Helminth External Mol. Quality Assesment 2018.1

Survey	Helminth External Mol. Quality Assesment 2018.1
Period	February 10, 2018 - May 25, 2018
Report for	
Subscriptions	15
Supervision	dr. J.J. van Hellemond (Coördinator)
MUSE manual	www.skml.nl/muse-manual.pdf

Scores	your score	MAP	reported
Qualitative	108	116	100%

Helminth External Mol. Quality Assesment 2018.1

Analyte		Trueness				Precision		Performance	
		your mean	ref.	cons.	SDbl	your SD	SDwl	this survey PS	cumulative PSc
Ascaris spp PCR	Cq	35.5		31.8	3.1	1.2	1.0		
Trichuris trichiura PCR	Cq	31.2		31.2	2.7	0.8	0.9		
Necator americanus PCR	Cq	33.6		28.7	4.3	2.6	1.8		
Ancylostoma spp PCR	Cq	38.5	38.5	0.0	0.0				
Strongyloides stercoralis PCR	Cq	28.3		28.2	3.0		2.3		
Schistosoma spp PCR	Cq	27.6		26.5	4.4	0.9	0.5		

Qualitative scores

Analyte	This survey				Cumulative			
	correct	incorrect	total	pictogram	correct	incorrect	total	pictogram
Ascaris spp PCR	8	0	8		8	0	8	
Trichuris trichiura PCR	5	1	6		5	1	6	
Necator americanus PCR	7	3	10		7	3	10	
Ancylostoma spp PCR	12	0	12		12	0	12	
Strongyloides stercoralis PCR	10	0	10		10	0	10	
Schistosoma spp PCR	12	0	12		12	0	12	

Helminth External Mol. Quality Assesment 2018.1

Sample :	A Ethanol fixed stool.
Patient :	Stool sample preserved in ethanol.
Question :	Molecular analysis (PCR) for the following helminths; Ascaris spp., Trichuris trichiura, Necator americanus, Ancylostoma spp., Strongyloides stercoralis and Schistosoma spp.
Remarks :	In case a PCR test is not performed by your laboratory, please unmark this test on the ‘settings page’.

Results	Expert values qual.	Expert values quant.	Your results qual.	Your results quant.	Score
Ascaris spp PCR	E Negative		Negative		2
Trichuris trichiura PCR	E Negative		Negative		2
Necator americanus PCR	E Negative		Negative		2
Ancylostoma spp PCR	E Negative		Negative		2
Strongyloides stercoralis PCR	E Negative		Negative		2
Schistosoma spp PCR	E Negative		Negative		2

E = Expert value Total 12

Helminth External Mol. Quality Assesment 2018.1

Sample :	B Ethanol fixed stool.
Patient :	Stool sample preserved in ethanol.
Question :	Molecular analysis (PCR) for the following helminths; Ascaris spp., Trichuris trichiura, Necator americanus, Ancylostoma spp., Strongyloides stercoralis and Schistosoma spp.
Remarks :	In case a PCR test is not performed by your laboratory, please unmark this test on the ‘settings page’.

Results	Expert values qual.	Expert values quant.	Your results qual.	Your results quant.	Score
Ascaris spp PCR	E Positive		Negative		
Trichuris trichiura PCR	E Negative		Negative		2
Necator americanus PCR	E Positive		Negative		
Ancylostoma spp PCR	E Negative		Negative		2
Strongyloides stercoralis PCR	E Positive	28.2 M Cq	Positive	28.3 Cq	
Schistosoma spp PCR	E Negative		Negative		2

E = Expert value ; M = Method group consensus value

Total 6

Helminth External Mol. Quality Assesment 2018.1

Sample :	C Ethanol fixed stool.
Patient :	Stool sample preserved in ethanol.
Question :	Molecular analysis (PCR) for the following helminths; Ascaris spp., Trichuris trichiura, Necator americanus, Ancylostoma spp., Strongyloides stercoralis and Schistosoma spp.
Remarks :	In case a PCR test is not performed by your laboratory, please unmark this test on the ‘settings page’.

Results	Expert values qual.	Expert values quant.	Your results qual.	Your results quant.	Score
Ascaris spp PCR	E Positive	29.2 M Cq	Positive	35.2 Cq	
Trichuris trichiura PCR	E Positive	35.4 M Cq	Negative		
Necator americanus PCR	E Negative		Negative		2
Ancylostoma spp PCR	E Negative		Negative		2
Strongyloides stercoralis PCR	E Negative		Negative		2
Schistosoma spp PCR	E Negative		Negative		2

E = Expert value ; M = Method group consensus value

Total 8

Helminth External Mol. Quality Assesment 2018.1

Sample :	D Ethanol fixed stool.
Patient :	Stool sample preserved in ethanol.
Question :	Molecular analysis (PCR) for the following helminths; Ascaris spp., Trichuris trichiura, Necator americanus, Ancylostoma spp., Strongyloides stercoralis and Schistosoma spp.
Remarks :	In case a PCR test is not performed by your laboratory, please unmark this test on the ‘settings page’.

Results	Expert values qual.	Expert values quant.	Your results qual.	Your results quant.	Score
Ascaris spp PCR	E Negative		Negative		2
Trichuris trichiura PCR	E Positive	34.6 M Cq	Negative		0
Necator americanus PCR	E Positive	33.0 M Cq	Negative		
Ancylostoma spp PCR	E Negative		Negative		2
Strongyloides stercoralis PCR	E Negative		Negative		2
Schistosoma spp PCR	E Negative		Negative		2

E = Expert value ; M = Method group consensus value

Total 8

Sample :	E Ethanol fixed stool.
Patient :	Stool sample preserved in ethanol.
Question :	Molecular analysis (PCR) for the following helminths; Ascaris spp., Trichuris trichiura, Necator americanus, Ancylostoma spp., Strongyloides stercoralis and Schistosoma spp.
Remarks :	In case a PCR test is not performed by your laboratory, please unmark this test on the 'settings page'.

Results	Expert values		Your results		Score
	qual.	quant.	qual.	quant.	
Ascaris spp PCR	E Negative		Negative		2
Trichuris trichiura PCR	E Negative		Negative		2
Necator americanus PCR	E Positive	29.3 M Cq	Positive	32.5 Cq	2
Ancylostoma spp PCR	E Negative		Negative		2
Strongyloides stercoralis PCR	E Negative		Negative		2
Schistosoma spp PCR	E Negative		Negative		2

E = Expert value ; M = Method group consensus value   Total 12

Helminth External Mol. Quality Assesment 2018.1

Sample :	F Ethanol fixed stool.
Patient :	Stool sample preserved in ethanol.
Question :	Molecular analysis (PCR) for the following helminths; Ascaris spp., Trichuris trichiura, Necator americanus, Ancylostoma spp., Strongyloides stercoralis and Schistosoma spp.
Remarks :	In case a PCR test is not performed by your laboratory, please unmark this test on the ‘settings page’.

Results	Expert values		Your results		Score
	qual.	quant.	qual.	quant.	
Ascaris spp PCR	E Positive	32.8 M Cq	Positive	36.2 Cq	2
Trichuris trichiura PCR	E Negative		Negative		2
Necator americanus PCR	E Positive	28.3 M Cq	Negative		0
Ancylostoma spp PCR	E Negative		Negative		2
Strongyloides stercoralis PCR	E Positive	38.6 M Cq	Negative		
Schistosoma spp PCR	E Negative		Negative		2

E = Expert value ; M = Method group consensus value

Total 8

Helminth External Mol. Quality Assesment 2018.1

Sample :	G Ethanol fixed stool.
Patient :	Stool sample preserved in ethanol.
Question :	Molecular analysis (PCR) for the following helminths; Ascaris spp., Trichuris trichiura, Necator americanus, Ancylostoma spp., Strongyloides stercoralis and Schistosoma spp.
Remarks :	In case a PCR test is not performed by your laboratory, please unmark this test on the ‘settings page’.

Results	Expert values qual.	Expert values quant.	Your results qual.	Your results quant.	Score
Ascaris spp PCR	E Positive	26.8 M Cq	Positive	30.9 Cq	2
Trichuris trichiura PCR	E Positive	30.9 M Cq	Positive	29.7 Cq	2
Necator americanus PCR	E Negative		Negative		2
Ancylostoma spp PCR	E Negative		Negative		2
Strongyloides stercoralis PCR	E Negative		Negative		2
Schistosoma spp PCR	E Negative		Negative		2

E = Expert value ; M = Method group consensus value

Total 12

Sample :	H Ethanol fixed stool.
Patient :	Stool sample preserved in ethanol.
Question :	Molecular analysis (PCR) for the following helminths; Ascaris spp., Trichuris trichiura, Necator americanus, Ancylostoma spp., Strongyloides stercoralis and Schistosoma spp.
Remarks :	In case a PCR test is not performed by your laboratory, please unmark this test on the 'settings page'.

Results	Expert values qual.	Expert values quant.	Your results qual.	Your results quant.	Score
Ascaris spp PCR	E Positive	32.7 M Cq	Positive	34.1 Cq	
Trichuris trichiura PCR	E Positive	31.1 M Cq	Positive	31.8 Cq	
Necator americanus PCR	E Negative		Negative		2
Ancylostoma spp PCR	E Negative		Negative		2
Strongyloides stercoralis PCR	E Negative		Negative		2
Schistosoma spp PCR	E Negative		Negative		2

E = Expert value ; M = Method group consensus value

Total 8

Sample :	Ethanol fixed stool.
Patient :	Stool sample preserved in ethanol.
Question :	Molecular analysis (PCR) for the following helminths; Ascaris spp., Trichuris trichiura, Necator americanus, Ancylostoma spp., Strongyloides stercoralis and Schistosoma spp.
Remarks :	In case a PCR test is not performed by your laboratory, please unmark this test on the 'settings page'.

Results	Expert values qual.	Expert values quant.	Your results qual.	Your results quant.	Score
Ascaris spp PCR	E Positive	35.7 M Cq	Negative	38.7 Cq	
Trichuris trichiura PCR	E Positive	32.5 M Cq	Negative		
Necator americanus PCR	E Positive	29.8 M Cq	Negative		0
Ancylostoma spp PCR	E Negative		Negative		2
Strongyloides stercoralis PCR	E Negative		Negative		2
Schistosoma spp PCR	E Positive	24.5 M Cq	Positive	26.0 Cq	2

E = Expert value ; M = Method group consensus value — Total 6

Helminth External Mol. Quality Assesment 2018.1

Sample :	J Ethanol fixed stool.
Patient :	Stool sample preserved in ethanol.
Question :	Molecular analysis (PCR) for the following helminths; Ascaris spp., Trichuris trichiura, Necator americanus, Ancylostoma spp., Strongyloides stercoralis and Schistosoma spp.
Remarks :	In case a PCR test is not performed by your laboratory, please unmark this test on the 'settings page'.

Results	Expert values qual.	Expert values quant.	Your results qual.	Your results quant.	Score
Ascaris spp PCR	E Negative		Negative		2
Trichuris trichiura PCR	E Positive	30.5 M Cq	Positive	30.5 Cq	
Necator americanus PCR	E Negative		Negative		2
Ancylostoma spp PCR	E Negative		Negative		2
Strongyloides stercoralis PCR	E Negative		Negative		2
Schistosoma spp PCR	E Negative		Negative		2

E = Expert value ; M = Method group consensus value

Total 10

Sample :	K Ethanol fixed stool.
Patient :	Stool sample preserved in ethanol.
Question :	Molecular analysis (PCR) for the following helminths; Ascaris spp., Trichuris trichiura, Necator americanus, Ancylostoma spp., Strongyloides stercoralis and Schistosoma spp.
Remarks :	In case a PCR test is not performed by your laboratory, please unmark this test on the ‘settings page’.

Results	Expert values qual.	Expert values quant.	Your results qual.	Your results quant.	Score
Ascaris spp PCR	E Positive	31.9 M Cq	Positive	36.3 Cq	2
Trichuris trichiura PCR	E Positive	32.3 M Cq	Positive	32.6 Cq	
Necator americanus PCR	E Positive	28.1 M Cq	Positive	34.8 Cq	2
Ancylostoma spp PCR	E Negative		Negative		2
Strongyloides stercoralis PCR	E Negative		Negative		2
Schistosoma spp PCR	E Positive	27.2 M Cq	Positive	29.0 Cq	2

E = Expert value ; M = Method group consensus value

Total 10

Helminth External Mol. Quality Assesment 2018.1

Sample :	L Ethanol fixed stool.
Patient :	Stool sample preserved in ethanol.
Question :	Molecular analysis (PCR) for the following helminths; Ascaris spp., Trichuris trichiura, Necator americanus, Ancylostoma spp., Strongyloides stercoralis and Schistosoma spp.
Remarks :	In case a PCR test is not performed by your laboratory, please unmark this test on the ‘settings page’.

Results	Expert values qual.	Expert values quant.	Your results qual.	Your results quant.	Score
Ascaris spp PCR	E Positive	33.4 M Cq	Positive	37.2 Cq	2
Trichuris trichiura PCR	E Positive	33.6 M Cq	Negative		
Necator americanus PCR	E Positive	30.4 M Cq	Negative		0
Ancylostoma spp PCR	E Negative		Negative		2
Strongyloides stercoralis PCR	E Negative		Negative		2
Schistosoma spp PCR	E Positive	27.8 M Cq	Positive	27.9 Cq	2

E = Expert value ; M = Method group consensus value

Total 8

Helminth External Mol. Quality Assesment 2018.1

Ascaris spp PCR

units: Cq

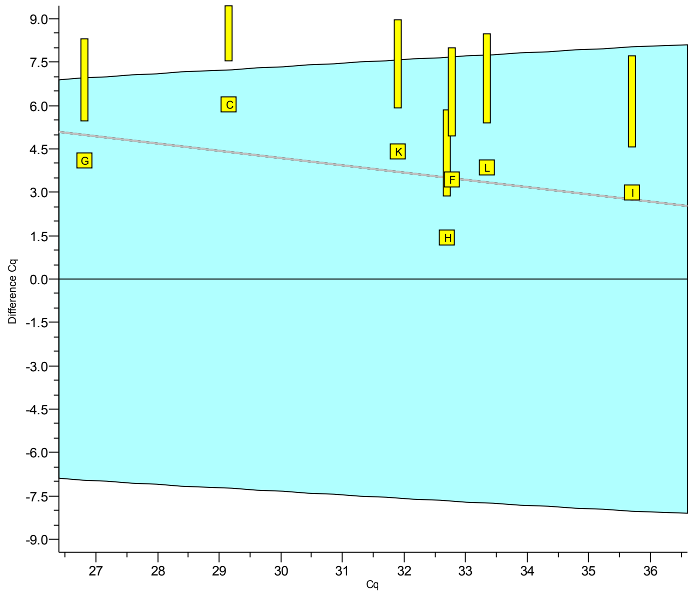

	2018.1	cumulative
Trueness	+12%	+12%
Precision	3.9%	3.9%
Number	7	7
Outliers	0	0
Sigma-TE		
Sigma-SA	4.1	4.1
Score pictogram		
Regression line	11.7 + 0.750.x	11.7 + 0.750.x
Consensus group	ALTM	
Method	Onbekend	
Analyser	Onbekend	
Your factor	0.0 + 1.000.x	
Method factor	0.0 + 1.000.x	

2018.1 A

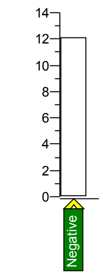

score : 2

2018.1 B

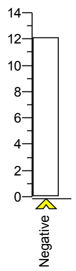

2018.1 C

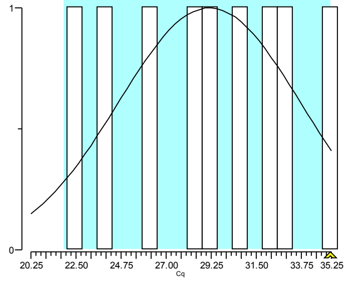

	cons.	meth.	ALTM	lab
mean	29.2	29.2	29.2	35.2
SD	4.6	4.6	4.6	
n	9	9	9	
no	0	0	0	
rec.	121%	121%	121%	

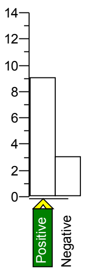

2018.1 D

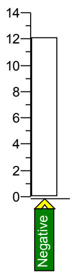

score : 2

Ascaris spp PCR

units: Cq

2018.1 E

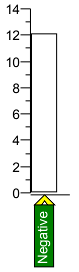

score : 2

2018.1 F

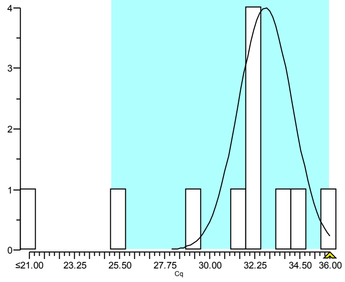

	cons.	meth.	ALTM	lab
mean	32.8	32.8	32.8	36.2
SD	1.3	1.3	1.3	
n	11	11	11	
no	2	2	2	
rec.	110%	110%	110%	

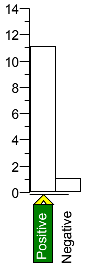

score : 2

2018.1 G

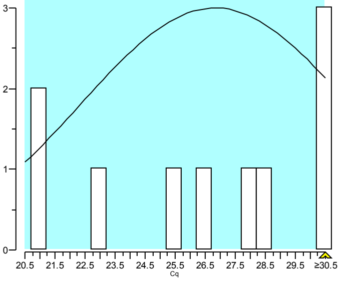

	cons.	meth.	ALTM	lab
mean	26.8	26.8	26.8	30.9
SD	4.4	4.4	4.4	
n	10	10	10	
no	0	0	0	
rec.	115%	115%	115%	

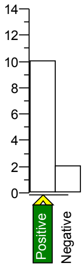

score : 2

2018.1 H

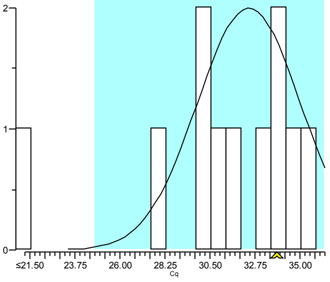

	cons.	meth.	ALTM	lab
mean	32.7	32.7	32.7	34.1
SD	2.6	2.6	2.6	
n	11	11	11	
no	1	1	1	
rec.	104%	104%	104%	

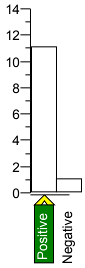

2018.1 I

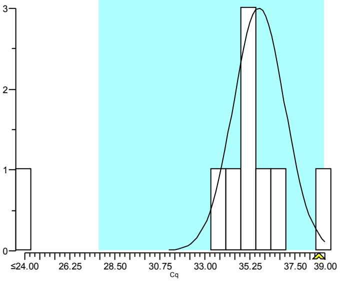

	cons.	meth.	ALTM	lab
mean	35.7	35.7	35.7	38.7
SD	1.3	1.3	1.3	
n	9	9	9	
no	1	1	1	
rec.	108%	108%	108%	

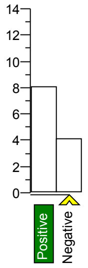

2018.1 J

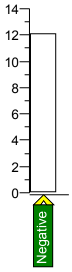

score : 2

2018.1 K

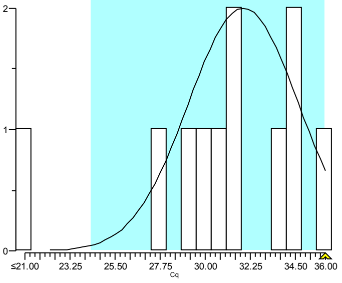

	cons.	meth.	ALTM	lab
mean	31.9	31.9	31.9	36.3
SD	2.8	2.8	2.8	
n	11	11	11	
no	1	1	1	
rec.	114%	114%	114%	

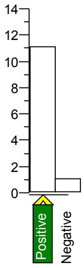

score : 2

2018.1 L

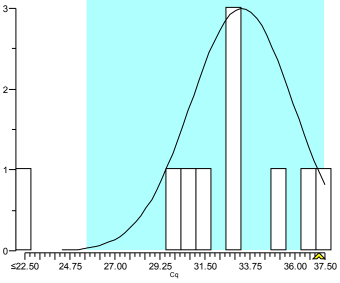

	cons.	meth.	ALTM	lab
mean	33.4	33.4	33.4	37.2
SD	2.6	2.6	2.6	
n	10	10	10	
no	1	1	1	
rec.	112%	112%	112%	

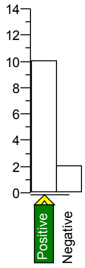

score : 2

Trichuris trichiura PCR

units: Cq

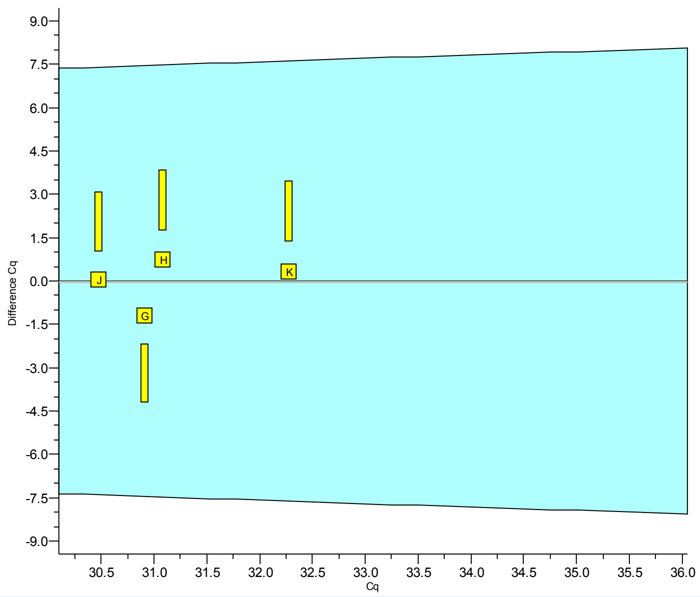

	2018.1	cumulative
Trueness	-0.12%	-0.12%
Precision	2.7%	2.7%
Number	4	4
Outliers	0	0
Sigma-TE		
Sigma-SA	6.0	6.0
Score pictogram		
Regression line	0.0 + 0.999.x	0.0 + 0.999.x

Consensus group	ALTM
Method	Onbekend
Analyser	Onbekend
Your factor	0.0 + 1.000.x
Method factor	0.0 + 1.000.x

2018.1 A

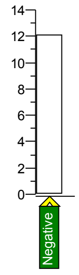

score : 2

2018.1 B

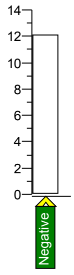

score : 2

2018.1 C

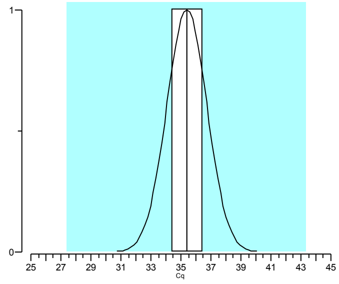

	cons.	meth.	ALTM	lab
mean	35.4	35.4	35.4	
SD				
n	2	2	2	
no	0	0	0	
rec.				

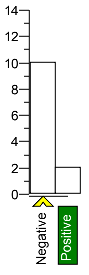

2018.1 D

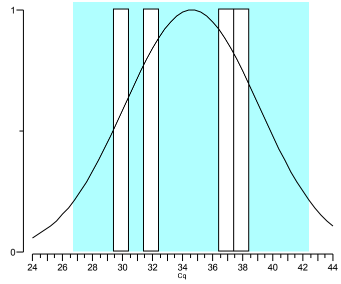

	cons.	meth.	ALTM	lab
mean	34.6	34.6	34.6	
SD				
n	4	4	4	
no	0	0	0	
rec.				

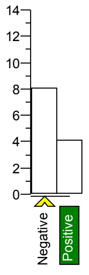

score : 0

Stichting Kwaliteitsbewaking Medische Laboratoriumdiagnostiek

MUSE

Helminth External Mol. Quality Assesment 2018.1

Trichuris trichiura PCR

units: Cq

2018.1 E

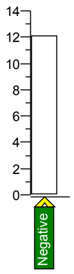

score : 2

2018.1 F

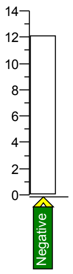

score : 2

2018.1 G

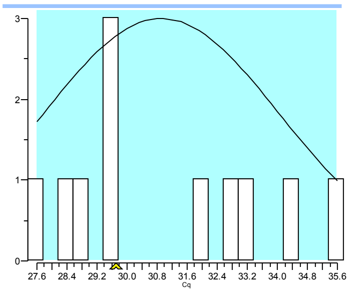

	cons.	meth.	ALTM	lab
mean	30.9	30.9	30.9	29.7
SD	3.1	3.1	3.1	
n	11	11	11	
no	0	0	0	
rec.	96%	96%	96%	

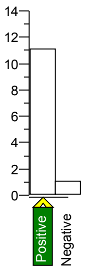

score : 2

2018.1 H

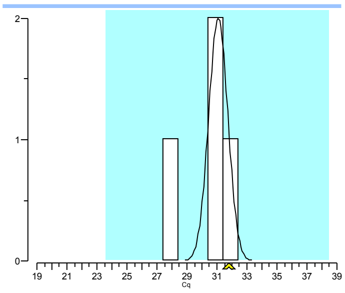

	cons.	meth.	ALTM	lab
mean	31.1	31.1	31.1	31.8
SD				
n	4	4	4	
no	1	1	1	
rec.	102%	102%	102%	

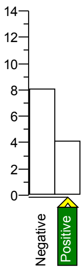

2018.1 I

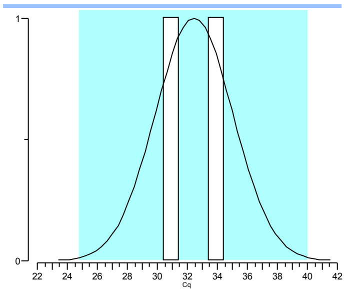

	cons.	meth.	ALTM	lab
mean	32.5	32.5	32.5	
SD				
n	2	2	2	
no	0	0	0	
rec.				

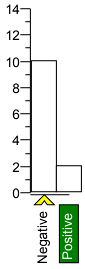

2018.1 J

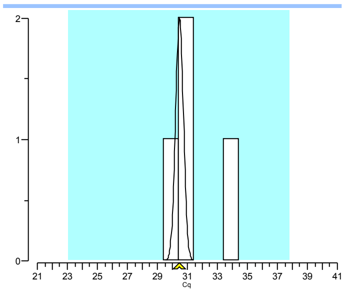

	cons.	meth.	ALTM	lab
mean	30.5	30.5	30.5	30.5
SD				
n	4	4	4	
no	1	1	1	
rec.	100%	100%	100%	

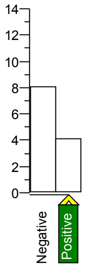

2018.1 K

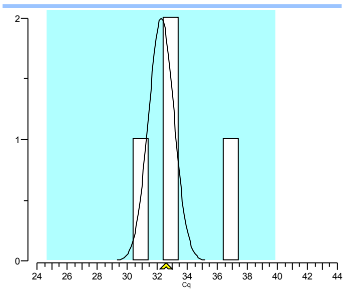

	cons.	meth.	ALTM	lab
mean	32.3	32.3	32.3	32.6
SD				
n	4	4	4	
no	1	1	1	
rec.	101%	101%	101%	

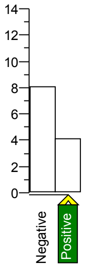

2018.1 L

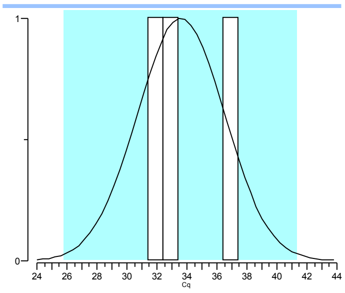

	cons.	meth.	ALTM	lab
mean	33.6	33.6	33.6	
SD				
n	3	3	3	
no	0	0	0	
rec.				

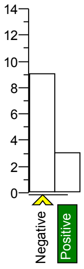

MUSE

Stichting Kwaliteitsbewaking Medische Laboratoriumdiagnostiek

Helminth External Mol. Quality Assesment 2018.1

Necator americanus PCR

units: Cq

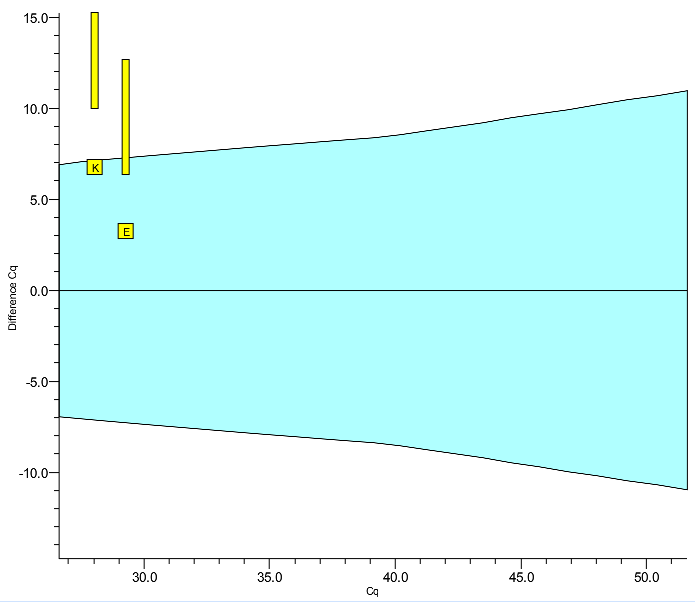

	2018.1	cumulative
Trueness	+17%	+17%
Precision	9.1%	9.1%
Number	2	2
Outliers	0	0
Sigma-TE		
Sigma-SA	1.7	1.7
Score pictogram		
Regression line		
Consensus group	ALTM	
Method	Onbekend	
Analyser	Onbekend	
Your factor	0.0 + 1.000.x	
Method factor	0.0 + 1.000.x	

2018.1 A

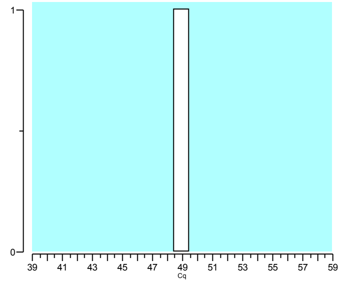

	cons.	meth.	ALTM	lab
mean	49.1	49.1	49.1	
SD				
n	1	1	1	
no	0	0	0	
rec.				

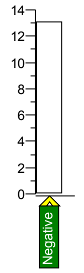

score : 2

2018.1 B

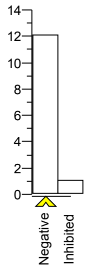

2018.1 C

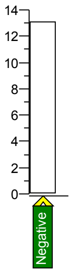

score : 2

2018.1 D

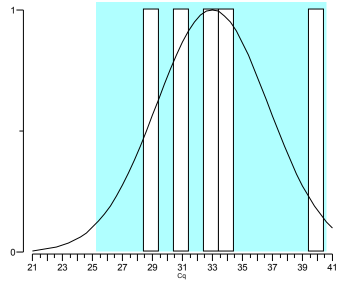

	cons.	meth.	ALTM	lab
mean	33.0	33.0	33.0	
SD	3.7	3.7	3.7	
n	5	5	5	
no	0	0	0	
rec.				

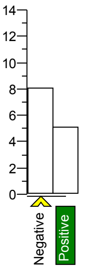

Helminth External Mol. Quality Assesment 2018.1

Necator americanus PCR

units: Cq

2018.1 E

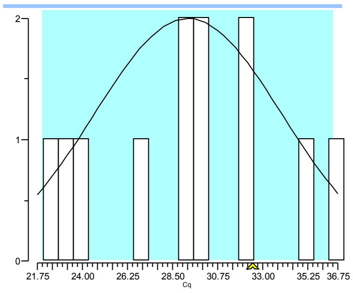

	cons.	meth.	ALTM	lab
mean	29.3	29.3	29.3	32.5
SD	4.7	4.7	4.7	
n	12	12	12	
no	0	0	0	
rec.	111%	111%	111%	

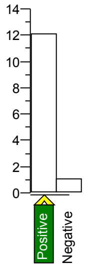

score : 2

2018.1 F

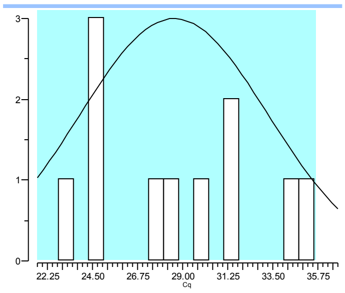

	cons.	meth.	ALTM	lab
mean	28.3	28.3	28.3	
SD	4.7	4.7	4.7	
n	11	11	11	
no	0	0	0	
rec.				

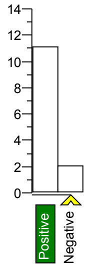

score : 0

2018.1 G

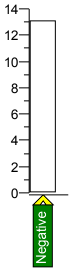

score : 2

2018.1 H

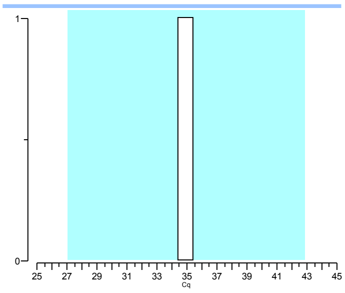

	cons.	meth.	ALTM	lab
mean	35.0	35.0	35.0	
SD				
n	1	1	1	
no	0	0	0	
rec.				

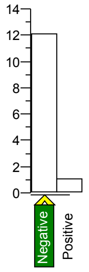

score : 2

2018.1 I

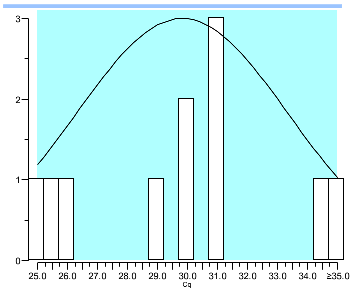

	cons.	meth.	ALTM	lab
mean	29.8	29.8	29.8	
SD	3.5	3.5	3.5	
n	11	11	11	
no	0	0	0	
rec.				

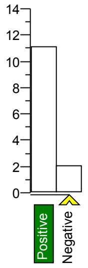

score : 0

2018.1 J

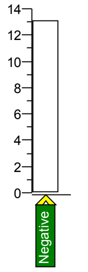

score : 2

2018.1 K

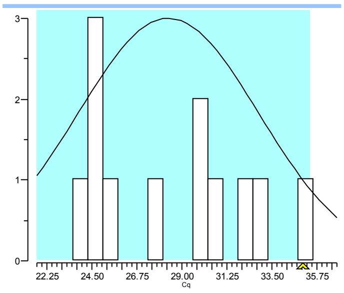

	cons.	meth.	ALTM	lab
mean	28.1	28.1	28.1	34.8
SD	4.5	4.5	4.5	
n	12	12	12	
no	0	0	0	
rec.	124%	124%	124%	

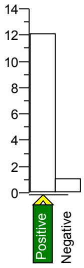

score : 2

2018.1 L

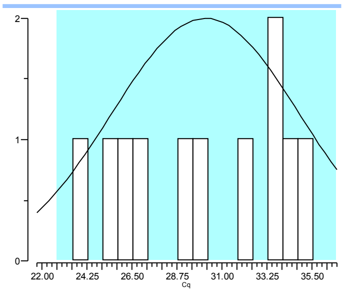

	cons.	meth.	ALTM	lab
mean	30.4	30.4	30.4	
SD	4.7	4.7	4.7	
n	11	11	11	
no	0	0	0	
rec.				

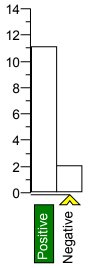

score : 0

Ancylostoma spp PCR

units: Cq

2018.1 A

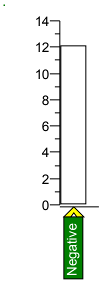

score : 2

2018.1 B

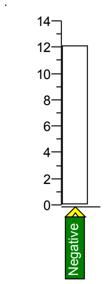

score : 2

2018.1 C

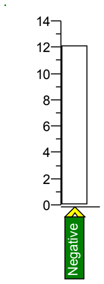

score : 2

2018.1 D

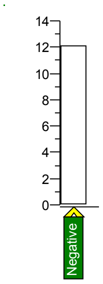

score : 2

2018.1 E

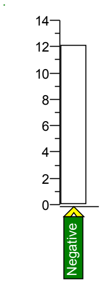

score : 2

2018.1 F

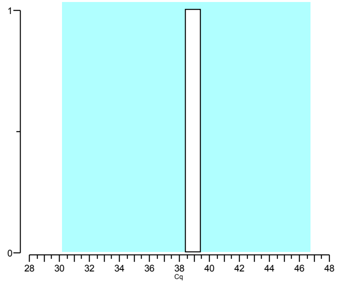

	cons.	meth.	ALTM	lab
mean	38.5	38.5	38.5	
SD				
n	1	1	1	
no	0	0	0	
rec.				

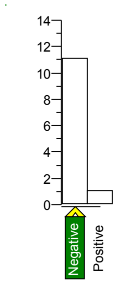

score : 2

2018.1 G

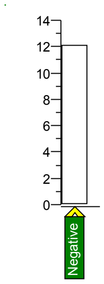

score : 2

2018.1 H

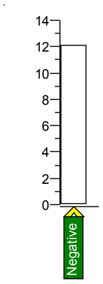

score : 2

Ancylostoma spp PCR

units: Cq

2018.1 I	2018.1 J	2018.1 K	2018.1 L
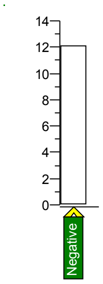	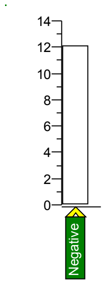	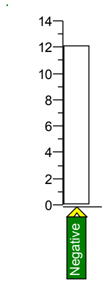	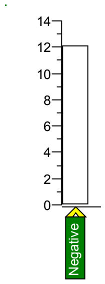
score : 2	score : 2	score : 2	score : 2

Strongyloides stercoralis PCR

units: Cq

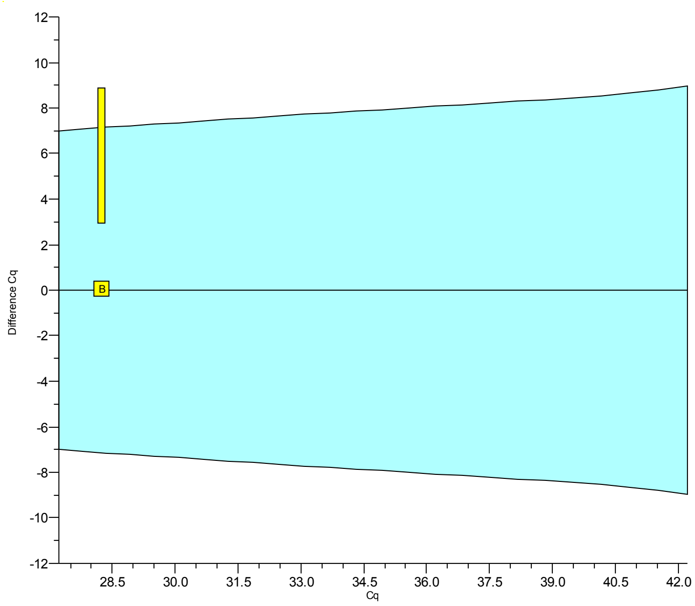

	2018.1	cumulative
Trueness	+0.19%	+0.19%
Precision		
Number	1	1
Outliers	0	0
Sigma-TE		
Sigma-SA	3.8	3.8
Score pictogram		
Regression line		

Consensus group	ALTM
Method	Onbekend
Analyser	Onbekend
Your factor	0.0 + 1.000.x
Method factor	0.0 + 1.000.x

Helminth External Mol. Quality Assesment 2018.1

Strongyloides stercoralis PCR

units: Cq

2018.1 A

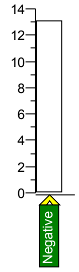

score : 2

2018.1 B

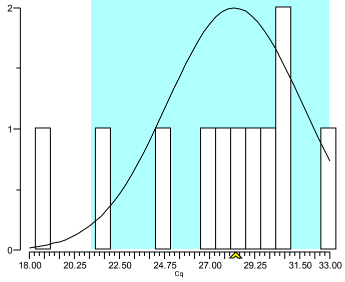

	cons.	meth.	ALTM	lab
mean	28.2	28.2	28.2	28.3
SD	3.4	3.4	3.4	
n	11	11	11	
no	0	0	0	
rec.	100%	100%	100%	

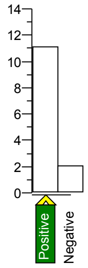

2018.1 C

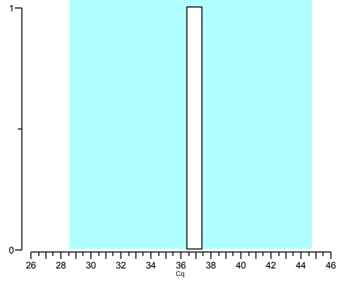

	cons.	meth.	ALTM	lab
mean	36.7	36.7	36.7	
SD				
n	1	1	1	
no	0	0	0	
rec.				

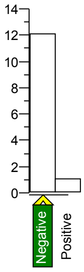

score : 2

2018.1 D

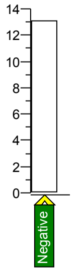

score : 2

2018.1 E

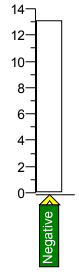

score : 2

2018.1 F

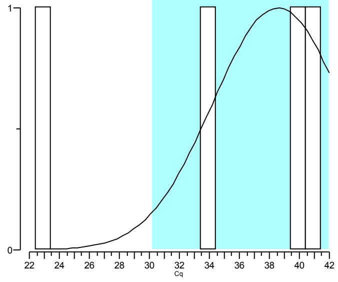

	cons.	meth.	ALTM	lab
mean	38.6	38.6	38.6	
SD				
n	4	4	4	
no	1	1	1	
rec.				

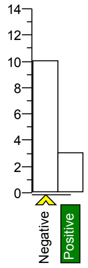

2018.1 G

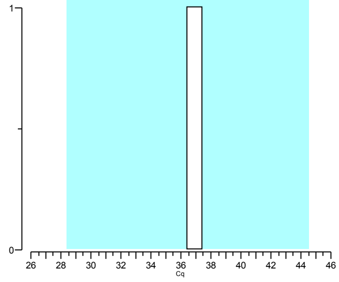

	cons.	meth.	ALTM	lab
mean	36.5	36.5	36.5	
SD				
n	1	1	1	
no	0	0	0	
rec.				

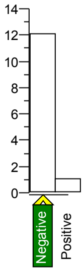

score : 2

2018.1 H

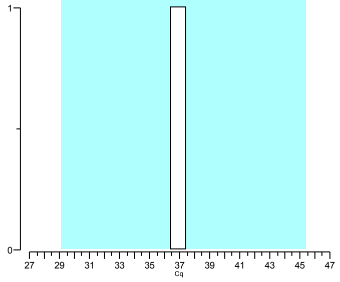

	cons.	meth.	ALTM	lab
mean	37.3	37.3	37.3	
SD				
n	1	1	1	
no	0	0	0	
rec.				

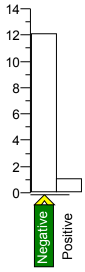

score : 2

Stichting Kwaliteitsbewaking Medische Laboratoriumdiagnostiek

MUSE

Helminth External Mol. Quality Assesment 2018.1

Strongyloides stercoralis PCR units: Cq

2018.1 I

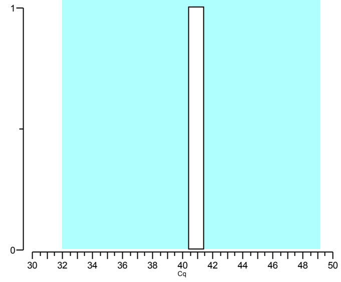

	cons.	meth.	ALTM	lab
mean	40.6	40.6	40.6	
SD				
n	1	1	1	
no	0	0	0	
rec.				

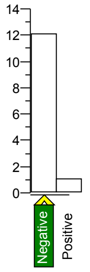

score : 2

2018.1 J

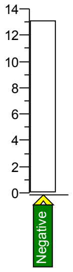

score : 2

2018.1 K

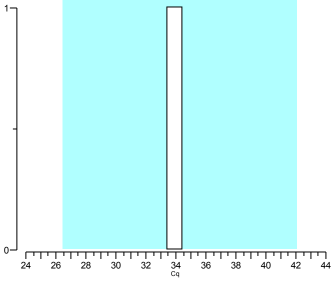

	cons.	meth.	ALTM	lab
mean	34.3	34.3	34.3	
SD				
n	1	1	1	
no	0	0	0	
rec.				

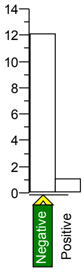

score : 2

2018.1 L

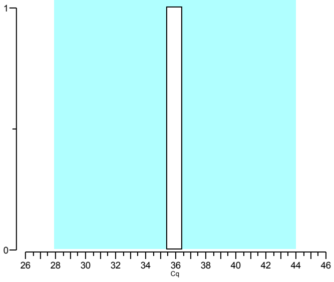

	cons.	meth.	ALTM	lab
mean	36.0	36.0	36.0	
SD				
n	1	1	1	
no	0	0	0	
rec.				

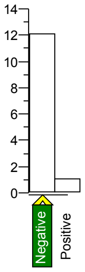

score : 2

Schistosoma spp PCR units: Cq

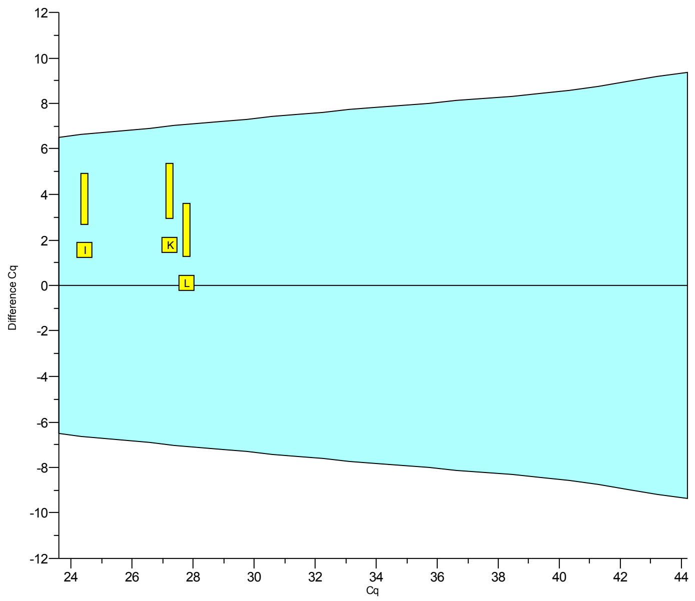

	2018.1	cumulative
Trueness	+4.2%	+4.2%
Precision	3.6%	3.6%
Number	3	3
Outliers	0	0
Sigma-TE		
Sigma-SA	6.0	6.0
Score pictogram		
Regression line		

Consensus group	ALTM
Method	Onbekend
Analyser	Onbekend
Your factor	0.0 + 1.000.x
Method factor	0.0 + 1.000.x

Schistosoma spp PCR

units: Cq

2018.1 A

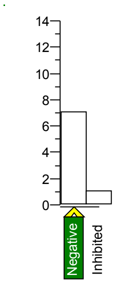

score : 2

2018.1 B

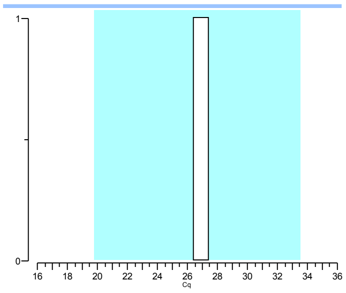

	cons.	meth.	ALTM	lab
mean	26.7	26.7	26.7	
SD				
n	1	1	1	
no	0	0	0	
rec.				

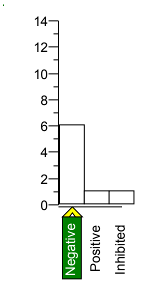

score : 2

2018.1 C

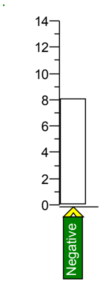

score : 2

2018.1 D

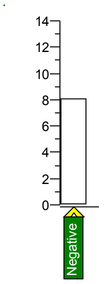

score : 2

2018.1 E

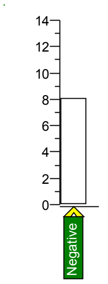

score : 2

2018.1 F

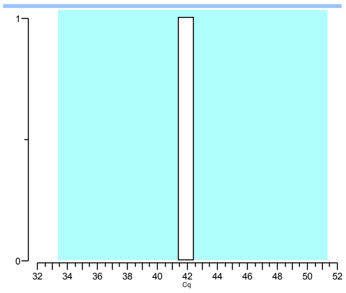

	cons.	meth.	ALTM	lab
mean	42.4	42.4	42.4	
SD				
n	1	1	1	
no	0	0	0	
rec.				

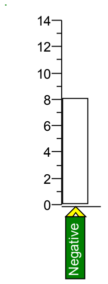

score : 2

2018.1 G

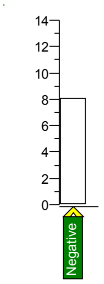

score : 2

2018.1 H

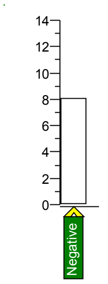

score : 2

Helminth External Mol. Quality Assesment 2018.1

Schistosoma spp PCR

units: Cq

2018.1 I

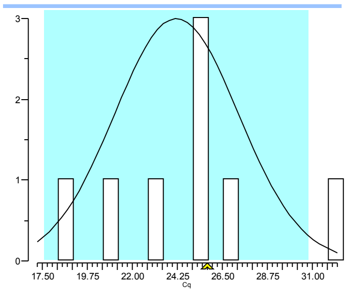

	cons.	meth.	ALTM	lab
mean	24.5	24.5	24.5	26.0
SD	3.1	3.1	3.1	
n	8	8	8	
no	1	1	1	
rec.	106%	106%	106%	

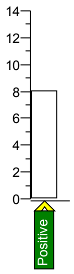

score : 2

2018.1 J

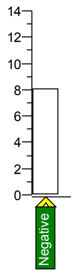

score : 2

2018.1 K

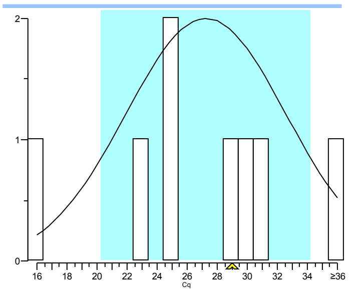

	cons.	meth.	ALTM	lab
mean	27.2	27.2	27.2	29.0
SD	5.3	5.3	5.3	
n	8	8	8	
no	0	0	0	
rec.	106%	106%	106%	

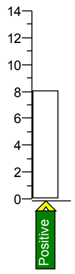

score : 2

2018.1 L

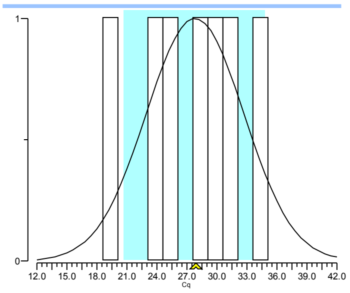

	cons.	meth.	ALTM	lab
mean	27.8	27.8	27.8	27.9
SD	4.9	4.9	4.9	
n	7	7	7	
no	0	0	0	
rec.	100%	100%	100%	

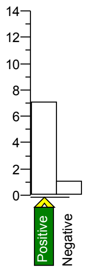

score : 2

Helminth External Mol. Quality Assesment 2018.2

Survey	Helminth External Mol. Quality Assesment 2018.2
Period	February 10, 2018 - May 25, 2018
Report for	
Subscriptions	15
Supervision	dr. J.J. van Hellemond (Coördinator)
MUSE manual	www.skml.nl/muse-manual.pdf

Scores	your score	MAP	reported
Qualitative	94	94	100%

Helminth External Mol. Quality Assesment 2018.2

Analyte		Trueness				Precision		Performance	
		your mean	ref.	cons.	SDbl	your SD	SDwl	this survey PS	cumulative PSc
Ascaris spp PCR	Cq	34.3		31.6	1.2	1.2	1.1		
Necator americanus PCR	Cq	33.0		27.9	6.7	1.6	1.7		
Strongyloides stercoralis PCR	Cq	30.6		31.4	2.0	1.2	1.6		
Schistosoma spp PCR	Cq	30.8		30.4	1.6	0.8	1.1		

Qualitative scores

Analyte	This survey				Cumulative			
	correct	incorrect	total	pictogram	correct	incorrect	total	pictogram
Ascaris spp PCR	8	0	8		16	0	16	
Trichuris trichiura PCR	8	0	8		13	1	14	
Necator americanus PCR	8	0	8		15	3	18	
Ancylostoma spp PCR	8	0	8		20	0	20	
Strongyloides stercoralis PCR	7	0	7		17	0	17	
Schistosoma spp PCR	8	0	8		20	0	20	

Helminth External Mol. Quality Assesment 2018.2

Sample :	A Purified DNA.
Patient :	Purified DNA sample in TE buffer.
Question :	Molecular analysis (PCR) for the following helminths; Ascaris spp., Trichuris trichiura, Necator americanus, Ancylostoma spp., Strongyloides stercoralis and Schistosoma spp.
Remarks :	In case a PCR test is not performed by your laboratory, please unmark this test on the 'settings page'.

Results	Expert values qual.	Expert values quant.	Your results qual.	Your results quant.	Score
Ascaris spp PCR	E Negative		Negative		2
Trichuris trichiura PCR	E Negative		Negative		2
Necator americanus PCR	E Negative		Negative		2
Ancylostoma spp PCR	E Negative		Negative		2
Strongyloides stercoralis PCR	E Positive	28.4 M Cq	Positive	26.6 Cq	2
Schistosoma spp PCR	E Negative		Negative		2

E = Expert value ; M = Method group consensus value

Total 12

Helminth External Mol. Quality Assesment 2018.2

Sample :	B Purified DNA.
Patient :	Purified DNA sample in TE buffer.
Question :	Molecular analysis (PCR) for the following helminths; Ascaris spp., Trichuris trichiura, Necator americanus, Ancylostoma spp., Strongyloides stercoralis and Schistosoma spp.
Remarks :	In case a PCR test is not performed by your laboratory, please unmark this test on the 'settings page'.

Results	Expert values qual.	Expert values quant.	Your results qual.	Your results quant.	Score
Ascaris spp PCR	E Negative		Negative		2
Trichuris trichiura PCR	E Negative		Negative		2
Necator americanus PCR	E Positive	29.5 M Cq	Positive	34.4 Cq	2
Ancylostoma spp PCR	E Negative		Negative		2
Strongyloides stercoralis PCR	E Negative		Negative		2
Schistosoma spp PCR	E Negative		Negative		2

E = Expert value ; M = Method group consensus value Total 12

Sample :	C Purified DNA.
Patient :	Purified DNA sample in TE buffer.
Question :	Molecular analysis (PCR) for the following helminths; Ascaris spp., Trichuris trichiura, Necator americanus, Ancylostoma spp., Strongyloides stercoralis and Schistosoma spp.
Remarks :	In case a PCR test is not performed by your laboratory, please unmark this test on the 'settings page'.

Results	Expert values		Your results		Score
	qual.	quant.	qual.	quant.	
Ascaris spp PCR	E Negative		Negative		2
Trichuris trichiura PCR	E Negative		Negative		2
Necator americanus PCR	E Negative		Negative		2
Ancylostoma spp PCR	E Negative		Negative		2
Strongyloides stercoralis PCR	E Negative		Negative		2
Schistosoma spp PCR	E Positive	28.7 M Cq	Positive	28.9 Cq	2

E = Expert value ; M = Method group consensus value

Total 12

Sample :	D Purified DNA.
Patient :	Purified DNA sample in TE buffer.
Question :	Molecular analysis (PCR) for the following helminths; Ascaris spp., Trichuris trichiura, Necator americanus, Ancylostoma spp., Strongyloides stercoralis and Schistosoma spp.
Remarks :	In case a PCR test is not performed by your laboratory, please unmark this test on the ‘settings page’.

Results	Expert values		Your results		Score
	qual.	quant.	qual.	quant.	
Ascaris spp PCR	E Positive	30.1 M Cq	Positive	32.8 Cq	2
Trichuris trichiura PCR	E Negative		Negative		2
Necator americanus PCR	E Negative		Negative		2
Ancylostoma spp PCR	E Negative		Negative		2
Strongyloides stercoralis PCR	E Negative		Negative		2
Schistosoma spp PCR	E Negative		Negative		2

E = Expert value ; M = Method group consensus value

Total 12

Sample :	E Purified DNA.
Patient :	Purified DNA sample in TE buffer.
Question :	Molecular analysis (PCR) for the following helminths; Ascaris spp., Trichuris trichiura, Necator americanus, Ancylostoma spp., Strongyloides stercoralis and Schistosoma spp.
Remarks :	In case a PCR test is not performed by your laboratory, please unmark this test on the ‘settings page’.

Results	Expert values qual.	Expert values quant.	Your results qual.	Your results quant.	Score
Ascaris spp PCR	E Negative		Negative		2
Trichuris trichiura PCR	E Negative		Negative		2
Necator americanus PCR	E Positive	26.2 M Cq	Positive	31.5 Cq	2
Ancylostoma spp PCR	E Negative		Negative		2
Strongyloides stercoralis PCR	E Negative		Negative		2
Schistosoma spp PCR	E Negative		Negative		2

E = Expert value ; M = Method group consensus value

Total 12

Sample :	F Purified DNA.
Patient :	Purified DNA sample in TE buffer.
Question :	Molecular analysis (PCR) for the following helminths; Ascaris spp., Trichuris trichiura, Necator americanus, Ancylostoma spp., Strongyloides stercoralis and Schistosoma spp.
Remarks :	In case a PCR test is not performed by your laboratory, please unmark this test on the 'settings page'.

Results	Expert values qual.	Expert values quant.	Your results qual.	Your results quant.	Score
Ascaris spp PCR	E Positive	33.2 M Cq	Positive	35.8 Cq	2
Trichuris trichiura PCR	E Negative		Negative		2
Necator americanus PCR	E Negative		Negative		2
Ancylostoma spp PCR	E Negative		Negative		2
Strongyloides stercoralis PCR	E Negative		Negative		2
Schistosoma spp PCR	E Negative		Negative		2

E = Expert value ; M = Method group consensus value

Total 12

Sample :	G Purified DNA.
Patient :	Purified DNA sample in TE buffer.
Question :	Molecular analysis (PCR) for the following helminths; Ascaris spp., Trichuris trichiura, Necator americanus, Ancylostoma spp., Strongyloides stercoralis and Schistosoma spp.
Remarks :	In case a PCR test is not performed by your laboratory, please unmark this test on the 'settings page'.

Results	Expert values		Your results		Score
	qual.	quant.	qual.	quant.	
Ascaris spp PCR	E Negative		Negative		2
Trichuris trichiura PCR	E Negative		Negative		2
Necator americanus PCR	E Negative		Negative		2
Ancylostoma spp PCR	E Negative		Negative		2
Strongyloides stercoralis PCR	E Negative		Negative		2
Schistosoma spp PCR	E Positive	32.1 M Cq	Positive	32.8 Cq	2

E = Expert value ; M = Method group consensus value

Total 12

Helminth External Mol. Quality Assesment 2018.2

Sample :	H Purified DNA.
Patient :	Purified DNA sample in TE buffer.
Question :	Molecular analysis (PCR) for the following helminths; Ascaris spp., Trichuris trichiura, Necator americanus, Ancylostoma spp., Strongyloides stercoralis and Schistosoma spp.
Remarks :	In case a PCR test is not performed by your laboratory, please unmark this test on the ‘settings page’.

Results	Expert values qual.	Expert values quant.	Your results qual.	Your results quant.	Score
Ascaris spp PCR	E Negative		Negative		2
Trichuris trichiura PCR	E Negative		Negative		2
Necator americanus PCR	E Negative		Negative		2
Ancylostoma spp PCR	E Negative		Negative		2
Strongyloides stercoralis PCR	E Positive	34.3 M Cq	Positive	34.5 Cq	
Schistosoma spp PCR	E Negative		Negative		2

E = Expert value ; M = Method group consensus value

Total 10

Ascaris spp PCR

units: Cq

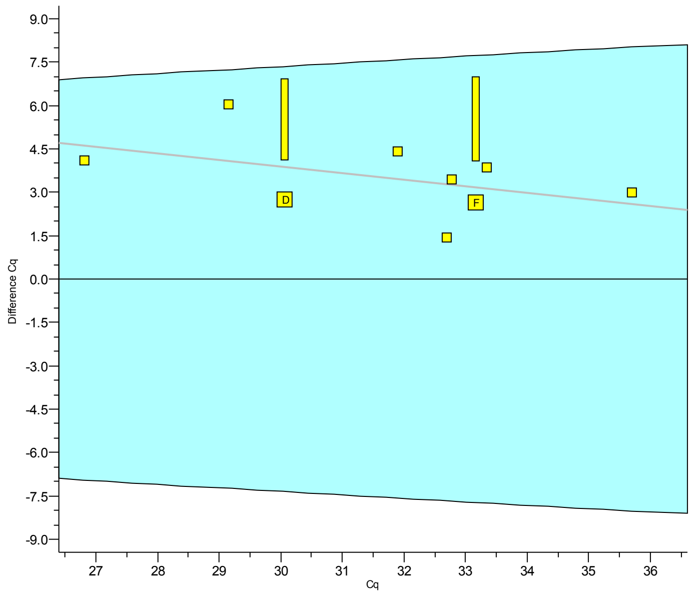

	2018.2	cumulative
Trueness	+8.5%	+11%
Precision	0.79%	3.7%
Number	2	9
Outliers	0	0
Sigma-TE		
Sigma-SA	5.1	4.4
Score pictogram		
Regression line		10.7 + 0.773.x

Consensus group	ALTM
Method	Onbekend
Analyser	Onbekend
Your factor	0.0 + 1.000.x
Method factor	0.0 + 1.000.x

2018.2 A

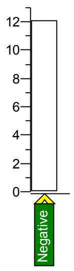

score : 2

2018.2 B

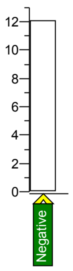

score : 2

2018.2 C

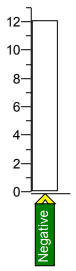

score : 2

2018.2 D

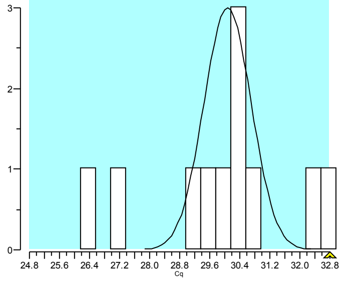

	cons.	meth.	ALTM	lab
mean	30.1	30.1	30.1	32.8
SD	0.6	0.6	0.6	
n	11	11	11	
no	4	4	4	
rec.	109%	109%	109%	

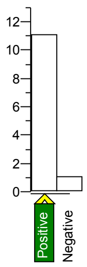

score : 2

Helminth External Mol. Quality Assesment 2018.2

Ascaris spp PCR

units: Cq

2018.2 E | 2018.2 F | 2018.2 G | 2018.2 H

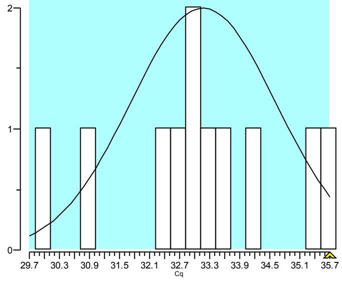

	cons.	meth.	ALTM	lab
mean	33.2	33.2	33.2	35.8
SD	1.5	1.5	1.5	
n	11	11	11	
no	0	0	0	
rec.	108%	108%	108%	

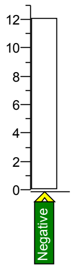

score : 2

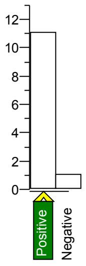

score : 2

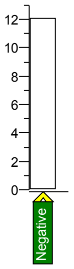

score : 2

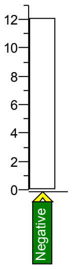

score : 2

Trichuris trichiura PCR

units: Cq

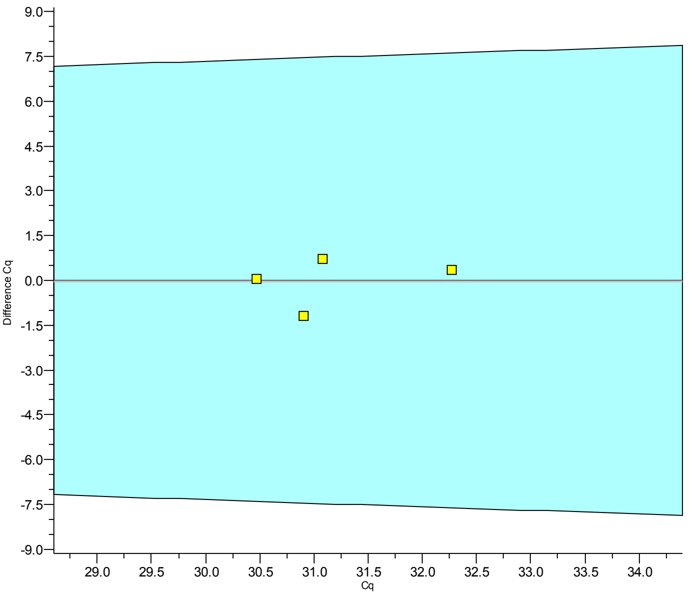

	2018.2	cumulative
Trueness		-0.12%
Precision		2.7%
Number		4
Outliers		0
Sigma-TE		
Sigma-SA		6.0
Score pictogram		
Regression line		0.0 + 0.999.x

Consensus group	ALTM
Method	Onbekend
Analyser	Onbekend
Your factor	0.0 + 1.000.x
Method factor	0.0 + 1.000.x

2018.2 A | 2018.2 B | 2018.2 C | 2018.2 D

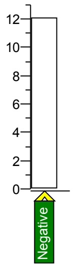

score : 2

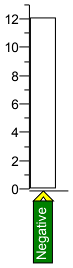

score : 2

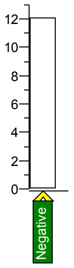

score : 2

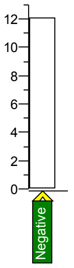

score : 2

Helminth External Mol. Quality Assesment 2018.2

Trichuris trichiura PCR

units: Cq

2018.2 E

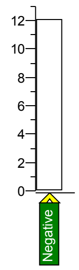

score : 2

2018.2 F

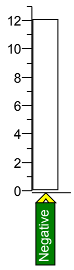

score : 2

2018.2 G

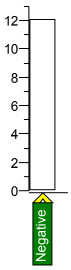

score : 2

2018.2 H

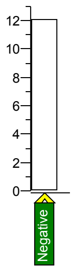

score : 2

Necator americanus PCR

units: Cq

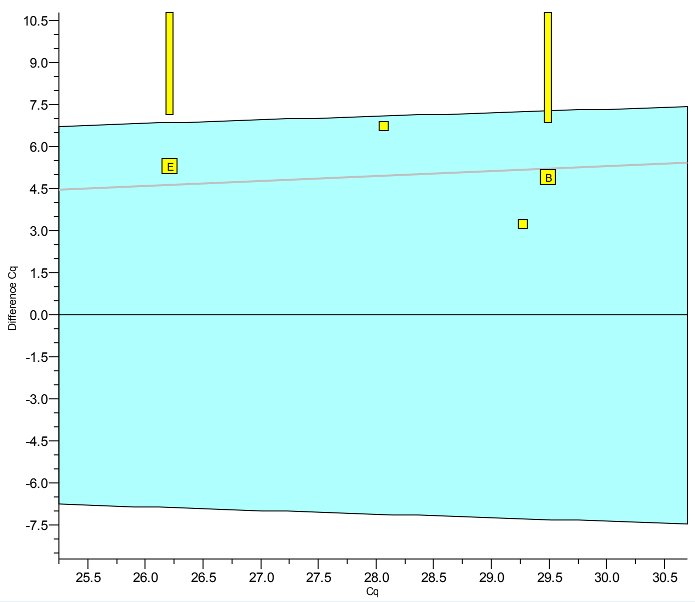

	2018.2	cumulative
Trueness	+18%	+18%
Precision	2.5%	5.6%
Number	2	4
Outliers	0	0
Sigma-TE		
Sigma-SA	2.2	2.2
Score pictogram		
Regression line		0.0 + 1.177.x

Consensus group	ALTM
Method	Onbekend
Analyser	Onbekend
Your factor	0.0 + 1.000.x
Method factor	0.0 + 1.000.x

2018.2 A

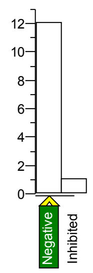

score : 2

2018.2 B

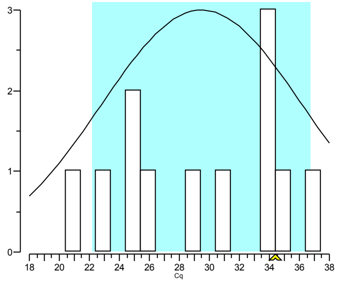

	cons.	meth.	ALTM	lab
mean	29.5	29.5	29.5	34.4
SD	6.7	6.7	6.7	
n	12	12	12	
no	0	0	0	
rec.	117%	117%	117%	

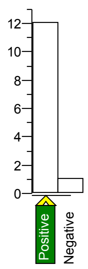

score : 2

2018.2 C

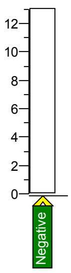

score : 2

2018.2 D

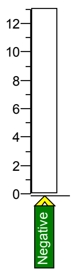

score : 2

Helminth External Mol. Quality Assesement 2018.2

Necator americanus PCR

units: Cq

2018.2 E

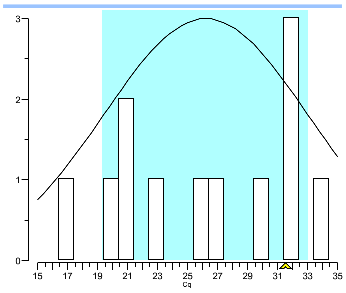

	cons.	meth.	ALTM	lab
mean	26.2	26.2	26.2	31.5
SD	6.8	6.8	6.8	
n	12	12	12	
no	0	0	0	
rec.	120%	120%	120%	

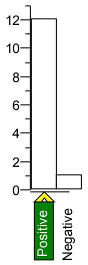

score : 2

2018.2 F

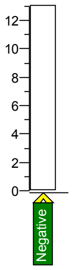

score : 2

2018.2 G

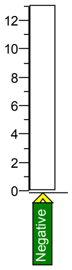

score : 2

2018.2 H

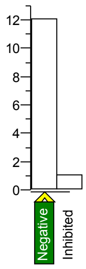

score : 2

Ancylostoma spp PCR

units: Cq

2018.2 A

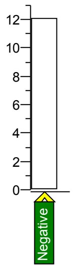

score : 2

2018.2 B

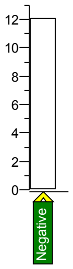

score : 2

2018.2 C

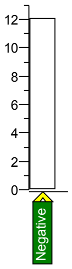

score : 2

2018.2 D

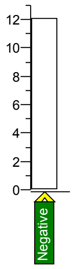

score : 2

2018.2 E

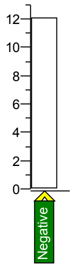

score : 2

2018.2 F

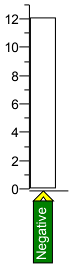

score : 2

2018.2 G

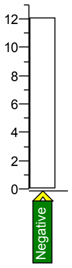

score : 2

2018.2 H

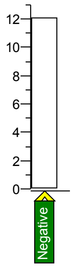

score : 2

Helminth External Mol. Quality Assesment 2018.2

Strongyloides stercoralis PCR

units: Cq

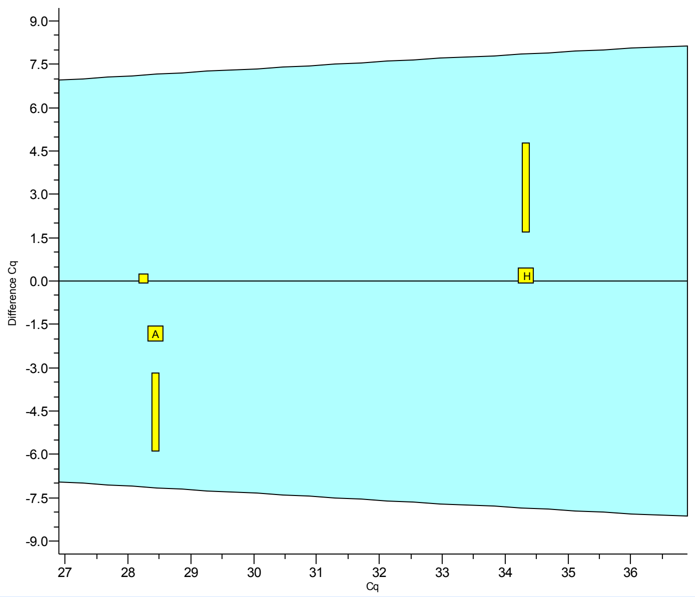

	2018.2	cumulative
Trueness	-2.7%	-1.8%
Precision	4.9%	3.8%
Number	2	3
Outliers	0	0
Sigma-TE		
Sigma-SA	6.0	6.0
Score pictogram		
Regression line		

Consensus group	ALTM
Method	Onbekend
Analyser	Onbekend
Your factor	0.0 + 1.000.x
Method factor	0.0 + 1.000.x

2018.2 A

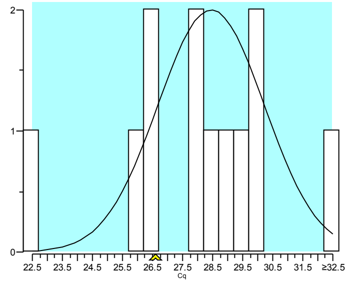

	cons.	meth.	ALTM	lab
mean	28.4	28.4	28.4	26.6
SD	1.8	1.8	1.8	
n	12	12	12	
no	1	1	1	
rec.	94%	94%	94%	

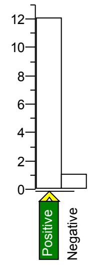

score : 2

2018.2 B

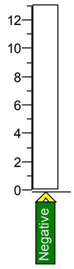

score : 2

2018.2 C

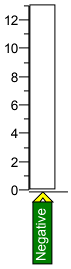

score : 2

2018.2 D

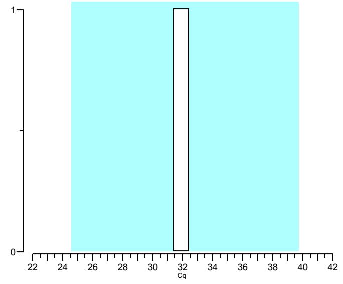

	cons.	meth.	ALTM	lab
mean	32.2	32.2	32.2	
SD				
n	1	1	1	
no	0	0	0	
rec.				

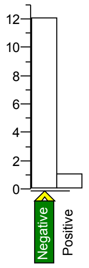

score : 2

Helminth External Mol. Quality Assesment 2018.2

Strongyloides stercoralis PCR

units: Cq

2018.2 E

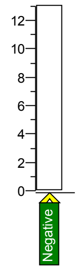

score : 2

2018.2 F

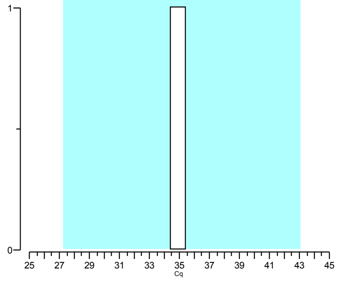

	cons.	meth.	ALTM	lab
mean	35.2	35.2	35.2	
SD				
n	1	1	1	
no	0	0	0	
rec.				

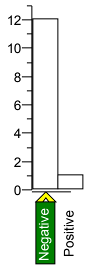

score : 2

2018.2 G

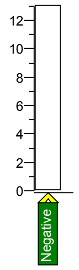

score : 2

2018.2 H

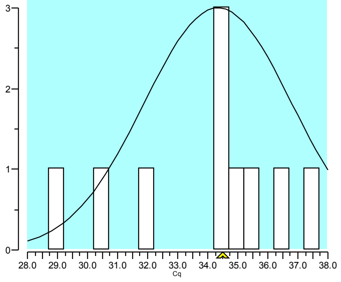

	cons.	meth.	ALTM	lab
mean	34.3	34.3	34.3	34.5
SD	2.5	2.5	2.5	
n	10	10	10	
no	0	0	0	
rec.	100%	100%	100%	

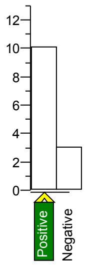

Schistosoma spp PCR

units: Cq

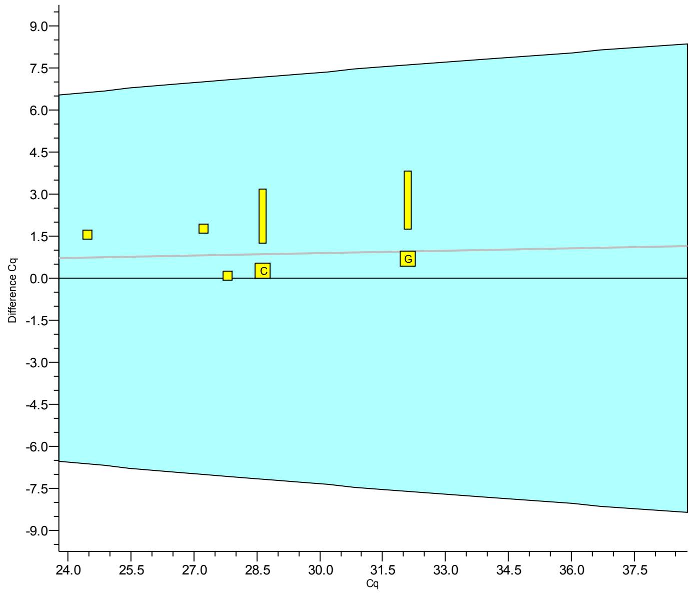

	2018.2	cumulative
Trueness	+1.5%	+3.1%
Precision	0.89%	2.8%
Number	2	5
Outliers	0	0
Sigma-TE		
Sigma-SA	6.0	6.0
Score pictogram	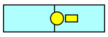	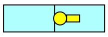
Regression line		0.0 + 1.029.x

Consensus group	ALTM
Method	Onbekend
Analyser	Onbekend
Your factor	0.0 + 1.000.x
Method factor	0.0 + 1.000.x

Helminth External Mol. Quality Assesment 2018.2

Schistosoma spp PCR units: Cq

2018.2 A

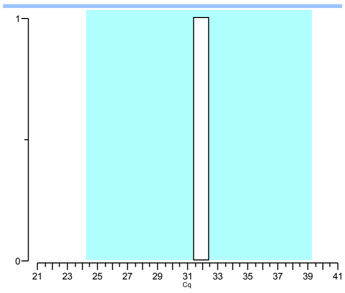

	cons.	meth.	ALTM	lab
mean	31.8	31.8	31.8	
SD				
n	1	1	1	
no	0	0	0	
rec.				

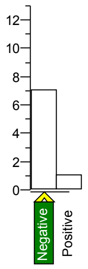

score : 2

2018.2 B

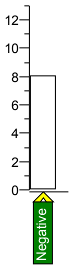

score : 2

2018.2 C

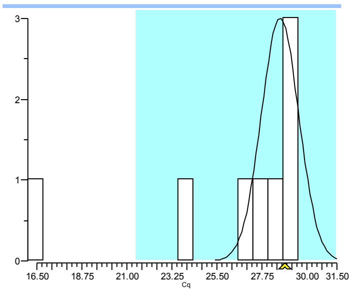

	cons.	meth.	ALTM	lab
mean	28.7	28.7	28.7	28.9
SD	0.9	0.9	0.9	
n	8	8	8	
no	2	2	2	
rec.	101%	101%	101%	

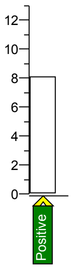

score : 2

2018.2 D

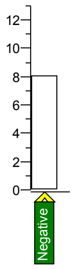

score : 2

2018.2 E

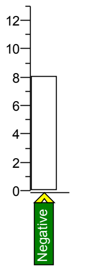

score : 2

2018.2 F

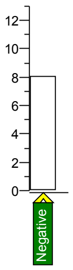

score : 2

2018.2 G

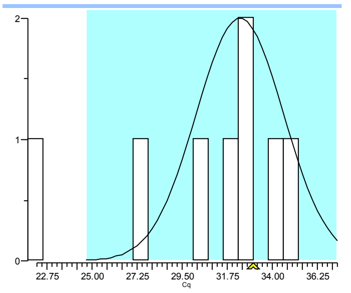

	cons.	meth.	ALTM	lab
mean	32.1	32.1	32.1	32.8
SD	2.2	2.2	2.2	
n	8	8	8	
no	1	1	1	
rec.	102%	102%	102%	

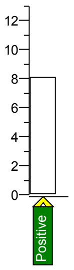

score : 2

2018.2 H

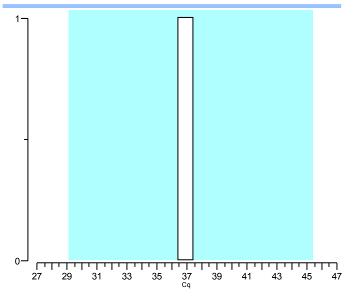

	cons.	meth.	ALTM	lab
mean	37.3	37.3	37.3	
SD				
n	1	1	1	
no	0	0	0	
rec.				

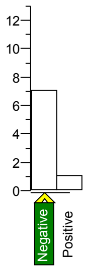

score : 2
